# Quantitative Measurement of Synthetic Repression Curves Reveals Design Challenges for Genetic Circuit Engineering under Growth Arrest

**DOI:** 10.1021/acssynbio.6c00123

**Published:** 2026-05-13

**Authors:** John P. Marken, Mark L. Prator, Bruce A. Hay, Richard M. Murray

**Affiliations:** Division of Biology and Biological Engineering, 6469 California Institute of Technology, 1200 E. California Blvd., Pasadena, California 91125, United States

**Keywords:** synthetic biology, genetic circuits, growth arrest, microbial physiology, gene regulation

## Abstract

Despite the fact that microbes in natural environments spend most of their time in growth arrest, we understand little about how this physiological state affects the performance of engineered genetic circuits. Here, we measure repression curves from a library of genetic NOT gates at single-cell resolution in *Escherichia coli* under both active growth and growth arrest to systematically investigate how growth arrest affects circuit behavior. We find that the impact of growth arrest on circuit performance is almost entirely dominated by a >100-fold reduction in unrepressed expression levels. Growth arrest caused gene expression noise to increase only moderately and had minimal impact on the sensitivity and sharpness of the repression curves. Our work shows both that conventional genetic circuit design paradigms are currently insufficient to develop circuits that can function properly under growth arrest, and that addressing the reduction in just a single performance parameter would be sufficient to resolve this problem. This work expands our understanding of bacterial gene regulation under growth arrest and lays the groundwork for new design paradigms that will be essential to ensure the safe and reliable performance of synthetic biology systems in real-world environments.

## Graphical abstract

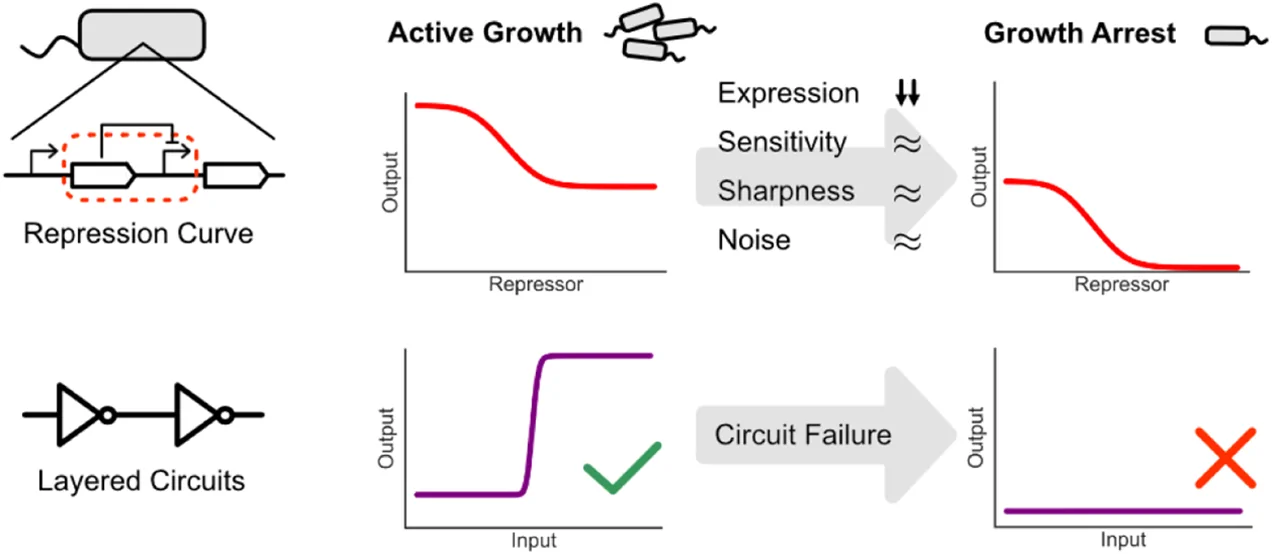

## Introduction

Genetic circuit engineering within living cells must contend with the fact that the physiological state of the host cell will change under different environmental conditions and thereby affect the behavior of the circuit.^1–3^ Characterization experiments have shown that the performance of even individual genetic parts can vary significantly across physiological states.^4–6^ Ensuring predictable and reliable circuit performance across different conditions is, therefore, a central goal of synthetic biology. Such efforts are becoming increasingly important as the field moves toward developing engineered microbes intended for use outside of controlled laboratory environments, such as the gut, soil, and engineered structures.^7,8^

Growth arrest is thought to be one of the most common physiological states for microbes in nature.^9^ A number of global physiological responses associated with growth arrest are known. These include a reduction in the number of ribosomes and RNA polymerases, compaction of the DNA and modifications to supercoiling, and major changes to metabolism.^9–11^ In order for complex genetic circuits to maintain long-term, reliable function in natural environments, it will be important to understand how such cellular responses to growth arrest affect the performance of these circuits’ constituent parts.

Layered repression architectures, where transcriptional repressors are wired together to implement desired dynamic behaviors or logical functions, are integral to current frameworks for genetic circuit design. The first engineered genetic circuits, the toggle switch^12^ and the Repressilator,^13^ were built with layered repression architectures, and the fact that repressors naturally encode a molecular analogue to NOT and NOR logic makes it possible in principle to use them to build genetic circuits encoding logical functions of arbitrary complexity.^14^ Many of the most complex genetic circuits constructed to date have therefore been built using layered repression architectures.^15,16^

The behavior of the individual repressors within a layered repression circuit is characterized by their repression curves, which are typically represented by the Hill relation

1 $$y=\frac{\beta }{1+{\left(x/K\right)}^{n}}+\alpha$$

where the input *x* represents the repressor concentration and the output *y* represents the expression level from the repressed promoter. The parameters β and α determine the unrepressed and maximally repressed expression levels of the output gene, and *K* and *n* represent the sensitivity and sharpness of the response to repressor concentration. We note that in this formulation, we interpret *n* as an intrinsic property of the repressor, *K* as an intrinsic property of the repressor-promoter pair, α as a phenomenological parameter that is determined both by the repressor-promoter pair and by global gene expression capacity, and β as a phenomenological parameter that is determined by the promoter and by global gene expression capacity (but not the repressor).

Proper circuit function requires that layered repression curves are properly aligned. A simple way to represent this condition is to say that the sensitivity of the downstream repressor must lie within the dynamic range of the upstream repressor, i.e., α_1_ < *K*_2_ < β_1_ + α_1_ (Figure 1). Factors like the Hill coefficient *n* or the level of gene expression noise affect the magnitude by which these inequalities need to hold. Characterizing the full repression curve is therefore essential to predicting the performance of the larger circuit.^15,17^ We illustrate the complexities associated with repressor alignment in Figure 1 using a NOT–NOT circuit, which layers two repressors in sequence to buffer signals while maintaining their logical value.

**Figure 1:**
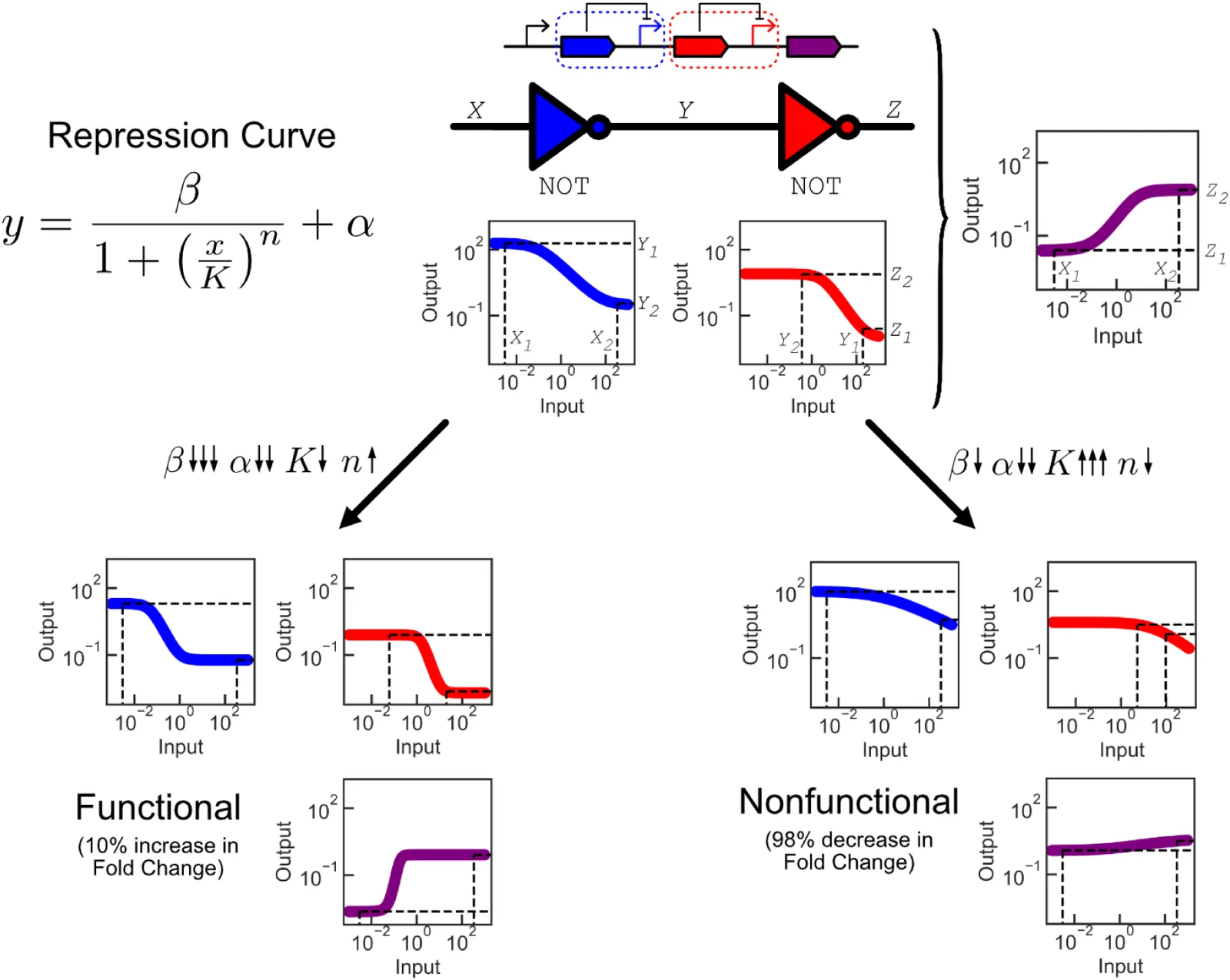
Schematic representation of the layering of repressors into larger circuits, illustrated by a NOT–NOT circuit. Inputs into the first gate (X) are transformed into output values (Y) according to the properties of the repression curve, which then become inputs into the second gate to generate outputs of the overall circuit (Z). Global changes to the parameters of the repression curve can lead to both enhancement (bottom left) and failure (bottom right) of circuit performance. The top circuit has parameters β_1_ = 200, α_1_ = 0.3, *K*_1_ = 0.1, *n*_1_ = 1 and β_2_ = 8, α_2_ = 0.01, *K*_2_ = 3, *n*_2_ = 1.5. The left transition scales all β, α, *K*, and *n* values by 1/10, 1/5, 1/2, and 2, respectively. The right transition scales all β, α, *K*, and *n* values by 1/2, 1/5, 10, and 1/2, respectively. All units are arbitrary.

However, despite the prominence of growth arrest in application environments like the gut or soil, there has been little investigation into its impact on the performance of engineered genetic components, including repressors. As a consequence, it is not clear *a priori* how the cell’s physiological responses to growth arrest would affect any of the parameters governing the repression curve, and whether these impacts would preserve or inhibit the ability of a circuit built from these repressors to function under growth arrest.

In this study, we set out to investigate whether there are any systematic effects associated with growth arrest on the performance of genetic repressors. We used a library of NOT gates based on dCas9-mediated repression of the T7 promoter at differing binding locations, engineered in *Escherichia coli*, as a model system to address this question. We found that NOT gates tended to retain their function under growth arrest with a reduced fold change, but that this occurred at expression levels that were ∼100-fold lower than in active growth. The shape parameters of the repression curves tended to be robust to the change in growth state, with the sensitivity and sharpness of the curves affected only minimally. Similarly, gene expression noise of NOT gate outputs increased only moderately under growth arrest. We further confirmed that these conclusions were consistent with the characterization of additional sets of NOT gates based on the PhlF repressor and a dCas9*:PhlF fusion protein. Within each set of gates, we generally observed that the repression behavior was more similar across gates under growth arrest than in active growth.

These results show that current design paradigms for layered repression-based circuits cannot lead to reliable circuit performance under growth arrest conditions, but that this failure is almost entirely tied to the change in a single parameter, β. As such, our findings reveal directions for future research by which these concrete design challenges could be overcome. Taken together, this work enhances our understanding of how growth arrest affects the quantitative properties of gene regulation and helps to develop a foundation for genetic design principles in natural environments.

## Results

### Design of General NOT Gate Architecture for Measurement in Growth Arrest

Although we do not know *a priori* exactly how growth arrest will affect the behavior of engineered NOT gates, our general understanding of microbial growth arrest suggests that our assay will need to be sensitive enough to detect low levels of gene expression and capture cell-to-cell variability in repression. Both of these points require modifications to the conventional procedures used in the field to measure repression curves. Typically, repression curves are measured by driving the expression of a fluorescent protein from a repressible promoter and expressing its cognate repressor from a separate inducible promoter (Figure 2a). By adding different concentrations of the inducer in separate experiments, one generates different concentrations of the repressor that lead to different expression levels of the output fluorescent protein.

**Figure 2:**
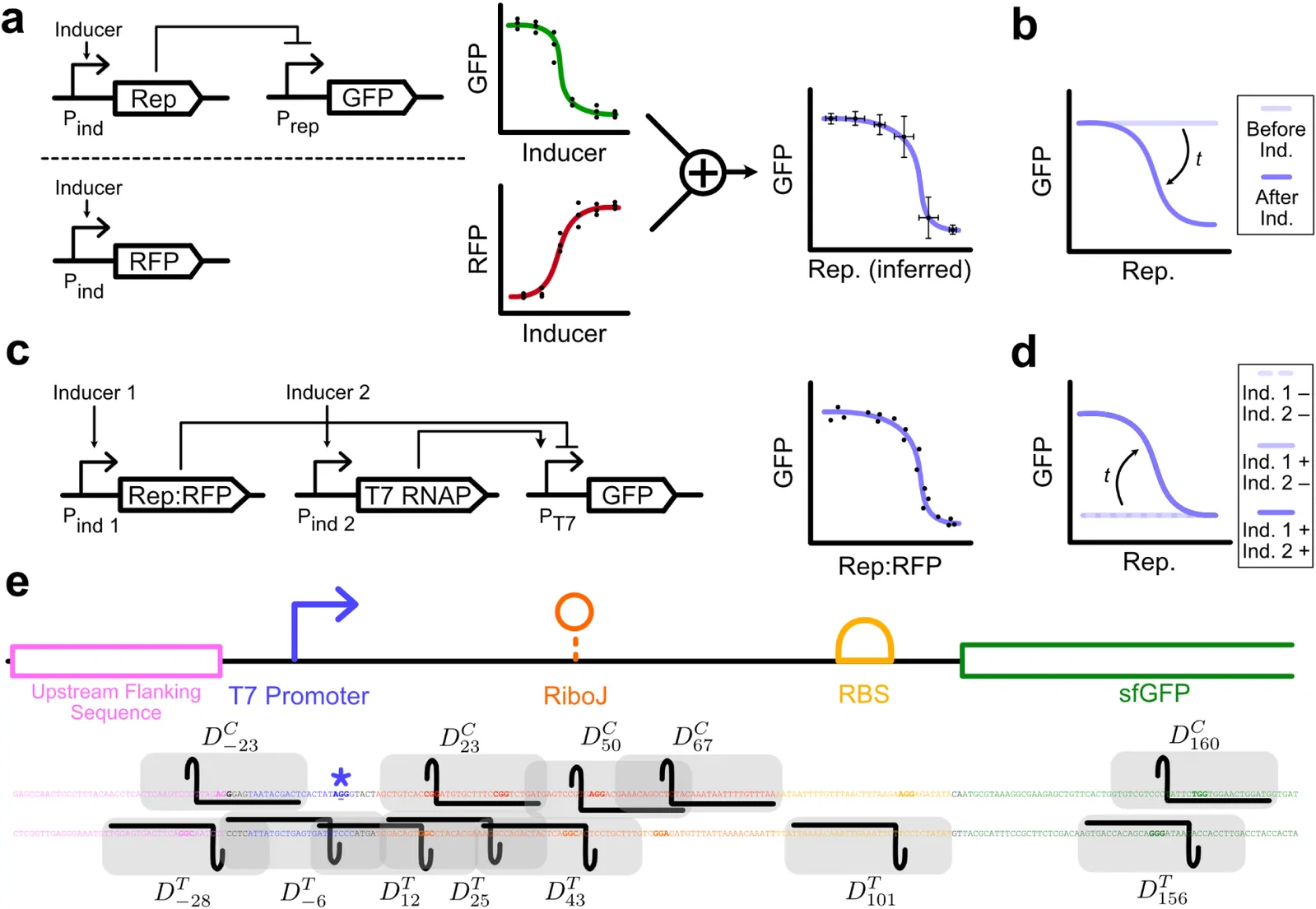
Design of NOT gates compatible with growth arrest measurements. (**a**) Schematic of the conventional procedure for measuring repression curves. The repressor (Rep) is expressed from an inducible promoter (P_ind_) to repress the expression of a fluorescent protein (GFP) from the repressible promoter (P_rep_). In a separate experiment, P_ind_ is used to express another fluorescent protein (RFP) to determine the expression level associated with a particular inducer concentration. These data are used to infer the repressor concentration in the first experiment, yielding a repression curve. (**b**) In the gate architecture in (a), GFP expression starts high in all conditions because P_rep_ is active in the absence of the repressor. The experimenter must wait for GFP levels to fall to their repressed level over time via dilution by cell division or active degradation. (**c**) Design of the T7-based NOT gate architecture used in this work. The repressor of the T7 promoter P_T7_ is fused to RFP, so single-cell measurements of both repressor and GFP levels can be made simultaneously. (**d**) For the gate architecture in (c), the GFP levels remain low until T7 RNAP is induced, ensuring that all observed GFP was expressed when P_T7_ was in a repressed regime. (**e**) Representation of dCas9 binding footprints for the 12 designed NOT gates. PAM sequences are bolded, and the +1 transcriptional start site is underlined and marked with a star.

Importantly, because the repressible promoter is unrepressed prior to the start of the experiment, one must wait for any output protein produced prior to repressor induction to be removed from the cell in order to accurately measure the expression level from the promoter in its repressed state (Figure 2b). This removal relies on either active degradation or dilution of the output protein through cell division, both of which pose challenges for measurements taken under growth arrest. One cannot rely on dilution to remove the initial output proteins because cells rarely divide under nutrient starvation. Meanwhile, applying active degradation to the output protein decreases its concentration associated with a particular level of promoter activity. As global gene expression levels are already predicted to be much lower under growth arrest,^10^ decreasing the signal strength in this way is undesirable.

We resolved these challenges by choosing to base our NOT gates around repression of the T7 promoter (Figure 2c). Because the T7 promoter does not exhibit cross-activation from *E. coli*’s native RNA polymerase, it remains inactive until its cognate RNA polymerase (T7 RNAP) is expressed. As such, we can perform a two-step procedure where we first induce the repressor and afterward induce the T7 RNAP in order to activate expression from the T7 promoter. This ensures that all observed output proteins were expressed when the promoter was under a repressed regime (Figure 2d).

Another challenge with applying conventional repression curve measurement techniques to growth arrest conditions is that the conventional approach does not measure the concentration of the repressor directly. Instead, the repressor concentration is assumed to be equivalent to that of a separate fluorescent protein expressed from the same promoter under the same conditions, in a separate experiment (Figure 2a). This approach does not allow us to measure cell-to-cell variability in repression because one cannot quantify the repressor concentration against the output protein concentration within the same cell.

We resolved this challenge by choosing to fuse our repressor to an orthogonal fluorescent protein, allowing direct single-cell measurements of repressor concentration alongside output protein concentration (Figure 2c). Furthermore, in order not to conflate the effects of cell–cell variability on gene expression with those from within-cell variability in plasmid-borne circuit copy number, we chose to integrate the entire system onto the *E. coli* genome.

### Design of NOT Gate Library

Having created a general gate architecture in which fluorescent protein-fused repressors target the T7 promoter, we next needed to choose the specific set of gates to test. Existing repression-based circuits have typically been constructed either from libraries of genomically mined transcription factors^18^ or from dCas9.^19^ Transcription factors exhibit a large diversity among various properties that affect repression, such as the protein’s size and its tendency for multimerization. They also vary in their actual mechanism of repression, including DNA looping, steric hindrance, or even stabilization of host RNA polymerases.^20,21^ This means that if two gates driven by different repressors behave differently under growth arrest, it will be difficult to disentangle which molecular factors led to these different responses.

We therefore chose to use dCas9-based repression as the basis for our NOT gate library so that all of the gates share the same repressor protein and vary only in where it binds. It is known that dCas9 can repress by inhibiting either transcriptional activation or elongation based on its binding position, and that in the latter case the binding orientation has a large impact on repression strength.^22^ Although to our knowledge there has been no systematic investigation of how dCas9 binding location affects the general properties of the repression curve as a whole, we reasoned that the existing evidence nonetheless suggests that varying the dCas9 binding position should create a library of NOT gates with diverse repression curve profiles.

We designed a construct expressing an sfGFP gene from the T7 promoter, incorporating the RiboJ ribozyme to serve as a genetic insulator by standardizing the 5′ untranslated region of the mRNA.^23^ We identified 12 PAM sites on both strands spanning 30 bp upstream to 160 bp downstream of the transcriptional start site and constructed gRNAs to target dCas9 to these positions, generating a library of 12 different NOT gates (Figure 2e). We label each gate as D^{T/C}^_N_, where the superscript indicates whether dCas9 binds to the Template or Coding strand and the subscript N indicates the location of the midpoint of the predicted 33 bp dCas9 binding footprint with respect to the transcription start site.^24^ We also created a 13th gate with an off-target gRNA, D_0_, as a negative control.

Because measuring the repressor concentration is a critical part of our assay, we fused dCas9 to a red fluorescent protein (mScarlet3) and expressed it from an inducible promoter (P_Tet_), while each gRNA was expressed from a strong constitutive promoter (P_OR1OR2_) to ensure that it is in stoichiometric excess. While dCas9-based NOT gates typically express dCas9 constitutively and titrate the concentration of gRNA, these two approaches are functionally equivalent in that they both lead to the titration of the amount of active repressor (the dCas9:gRNA complex). The T7 RNAP was separately expressed from an orthogonal inducible promoter (P_LacO_). Both inducer molecules, atc and IPTG, are not metabolized by *E. coli*, ensuring that cells remain in growth arrest even upon the addition of inducer.

### Design and Validation of Measurement Assay

Studies in the literature utilize a number of different approaches to induce growth arrest, which can have different implications for the resulting physiological responses. We chose to follow the general approach used by Bergkessel and Delavaine, which allows a culture of cells to naturally reach the stationary phase before washing and diluting them into carbon-limited media.^25^ This approach simulates the gradual entry into starvation that is likely more representative of natural environments while also avoiding potential confounding effects from the high densities associated with stationary-phase cultures.

We then set out to design a measurement procedure that allows the behavior of a genetic circuit in the same population of cells to be compared under two different growth conditions. While synthetic biologists often measure the performance of their circuits in both exponential phase and stationary phase to assess robustness across growth phases, this is typically done by activating the circuit in exponential phase, measuring its output, and then waiting until that same population reaches stationary phase before remeasuring the output.^18,26^ This approach captures how the circuit’s performance persists over time but does not capture its intrinsic behavior under growth arrest, as the circuit is not activated during growth arrest specifically, and outputs from its prior activation during exponential phase could carry over into stationary phase.

We therefore designed a measurement procedure that can independently measure a circuit’s behavior under both growth arrest and active growth (Figure 3a, Methods). An overnight culture of cells engineered with the NOT gate is split into 24 different cultures, half of which are washed and diluted into carbon-limited media alongside the addition of 11 different concentrations of atc to induce dCas9 expression to various levels. 24 h later, 1 mM IPTG is added to 11 of the cultures to induce T7 RNAP expression, with the 12th culture remaining as an uninduced negative control. The same induction procedure is applied to the other 12 cultures from the initial split, except that these are repeatedly diluted into fresh batches of the original growth media to ensure the cells remain actively growing. 24 h after the T7 RNAP induction, all cultures are measured for mScarlet3 and sfGFP expression via flow cytometry.

**Figure 3:**
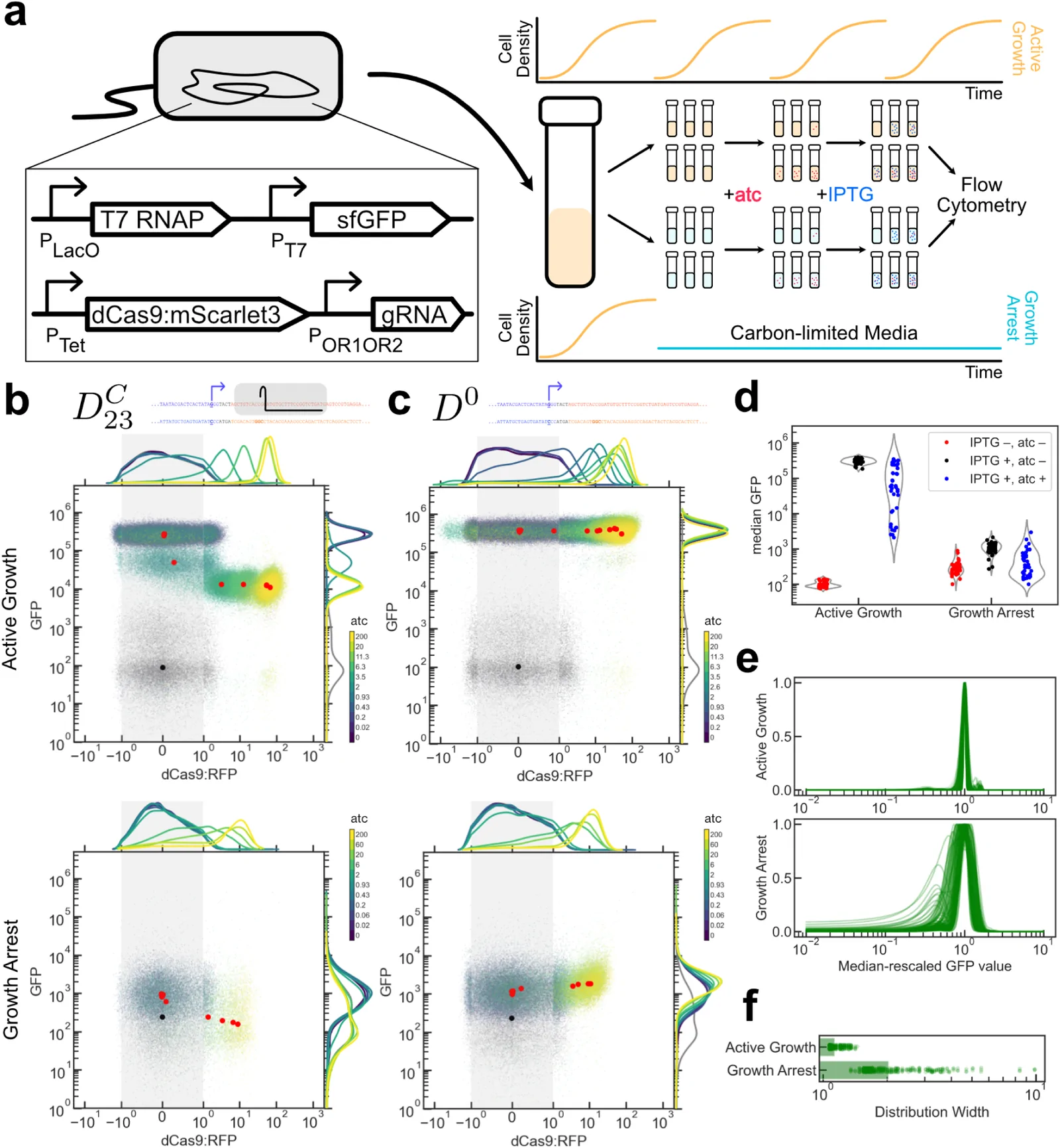
Measurement of NOT gate repression curves under active growth and growth arrest. (**a**) Schematic representation of the measurement assay. (**b**) Representative example of a repression curve in both active growth and growth arrest. Data from Gate D^C^_23_. (**c**) Representative example of the negative control circuit driven by an off-target gRNA (Gate D_0_). Points in (**b**,**c**) are colored according to the atc concentration (in ng/mL as shown in color bars), with gray points from the condition where no IPTG was added. Circles represent the median GFP and RFP values for each condition. RFP values are plotted on a symmetric log scale^27^ where the gray-shaded region is linear. Distributions along the horizontal and vertical axes correspond to the univariate distributions of RFP or GFP values, respectively, for each induction condition. Full plots of each of the three replicates for each gate are in Supplemental Figures 1a–13a. All GFP and RFP values are given in absolute units (MEFL and MEPETR, respectively). (**d**) Median GFP values across all gates at selected induction conditions. IPTG −/+ corresponds to 0 or 1 mM induction of T7 RNAP, and atc −/+ corresponds to 0 or maximal (200 ng/mL) induction of repressor. (**e**) Kernel density estimates (KDEs) of log-transformed GFP values across all experimental conditions where T7 RNAP was induced, rescaled to the median of the distribution, and normalized to a maximal value of 1. (**f**) Width of each KDE in (e), defined by the distance in log space between the two points where the KDE crosses the value of 0.2. Bars show the geometric mean.

Our assay was able to measure repression behavior in both active growth and growth arrest for the NOT gates in our library, with dCas9 concentrations spanning a wide range of values (from 0 to ∼100 MEPETR in active growth and to ∼10 MEPETR in growth arrest) and driving a decrease in GFP levels (Figure 3b, Supplemental Figure 1a–12a). The off-target gate D_0_ did not show a decrease in GFP associated with increasing dCas9 concentration (Figure 3c, Supplemental Figure 13a), indicating that observed decreases in GFP levels are indeed due to the impact of active repression by dCas9, rather than expression burden.

We also saw that the unrepressed GFP level was similar across all gates within a growth condition, suggesting that leaky expression of dCas9 in the absence of induction was not a notable issue (Figure 3d). Additionally, we saw a clear separation in GFP values between conditions where T7 RNAP was induced or uninduced in the absence of a repressor across all gates, suggesting that leaky expression of T7 RNAP was also not an issue (*p* < 10^–6^ for both growth conditions, paired *t* test). However, the overall dynamic range of P_T7_ activation under growth arrest (3.75-fold) was significantly lower than in active growth (3000-fold). This reduction in dynamic range was primarily due to growth arrest imposing a large reduction in P_T7_’s ON state (geometric mean of 300,000 MEFL to 1,000 MEFL), as compared with the smaller increase in P_T7_’s OFF state (geometric mean of 100 MEFL to 275 MEFL) (Figure 3d). Overall, these results showed that our assay is able to reliably measure repression curves from our NOT gate library in both active growth and growth arrest.

### Gene Expression Noise in NOT Gate Outputs Increases Moderately under Growth Arrest

We next investigated how growth arrest impacted the level of noise in the NOT gates’ output. We plotted kernel density estimates of the GFP values from every experimental condition where T7 RNAP was induced and rescaled them to their median value to overlay them against each other (Figure 3e). GFP distributions in active growth were dominated by a tight peak around the median, as expected. GFP distributions in growth arrest, however, tended to be wider, and 24% of them exhibited notable nonunimodality, compared to none in active growth (Methods). Calculating the width of the rescaled distributions found that growth arrest increased the average distribution width by 1.8-fold (Figure 3f). These results show that growth arrest applies a consistent but moderate increase in the noise of NOT gate outputs.

### dCas9 Binding Location Affects Repression Curve Properties during Active Growth

We next analyzed our data to determine whether our library of NOT gates, in which gates differ from each other based on the binding location of dCas9, indeed generated a diversity of repression curves, as predicted. Although prior work has shown that dCas9 binding location affects the extent of overall repression,^22,28,29^ to our knowledge, our dataset is the first to measure full repression curves across different dCas9 binding locations. In order to extract the parameters of the repression curve from our measurements, we performed Bayesian parameter estimation on the data from each gate to obtain a distribution of values for each of the four parameters in the Hill repression function (eq 1) for each growth condition (Figure 4a).

**Figure 4:**
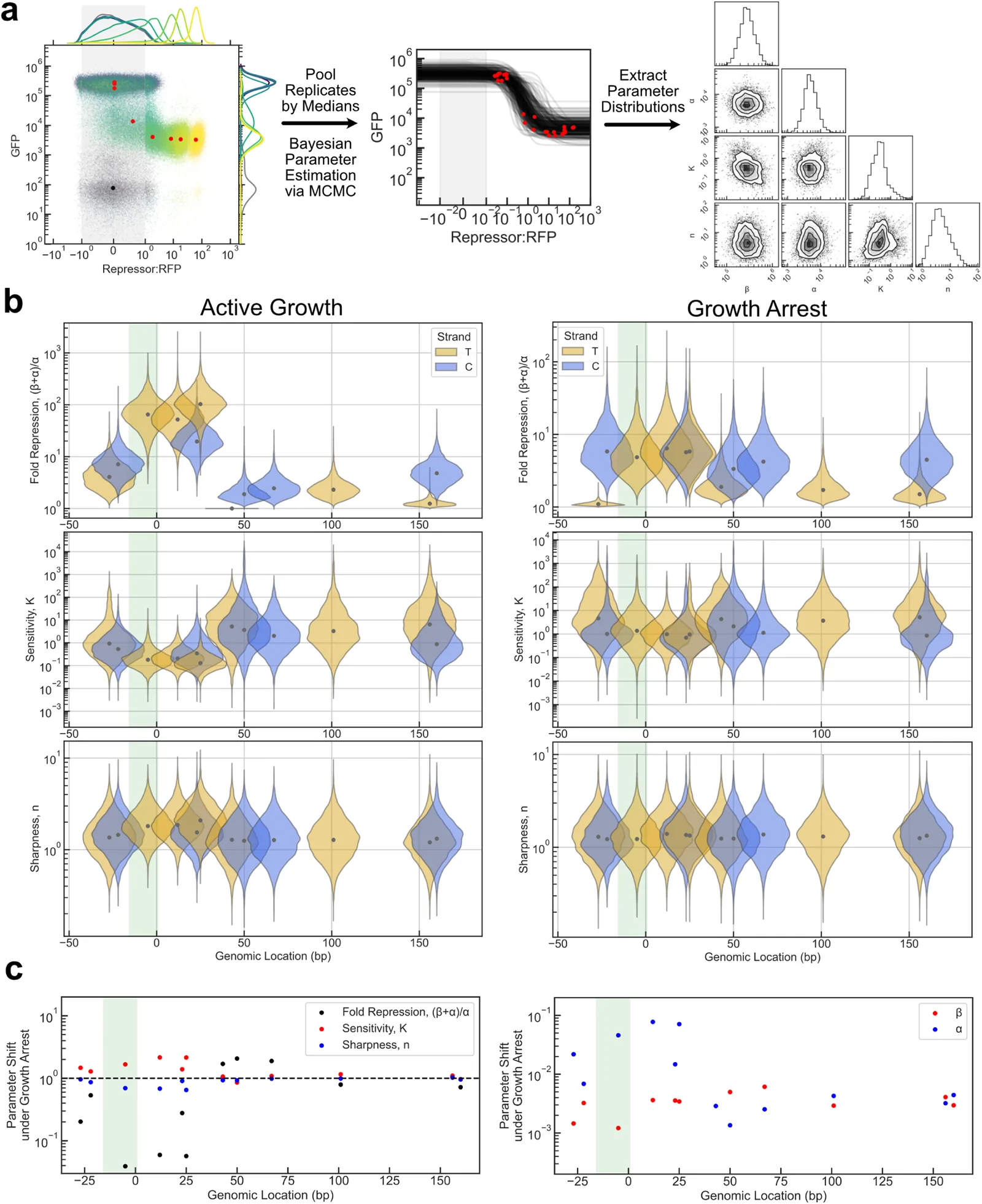
Repression curve behavior of dCas9 NOT gate library. (**a**) Schematic of the Bayesian Parameter Estimation (BPE) procedure for obtaining distributions of parameter values. Median (GFP, RFP) values from each induction condition are pooled across replicates to generate a master repression curve, to which the Hill Repression function (eq 1) is fit using Markov Chain Monte Carlo (MCMC)-based BPE. Full plots of each NOT gate in each growth condition are given in Supplemental Figures 1b–13b. (**b**) Posterior parameter distributions for fold repression (β + α)/α, sensitivity *K*, and sharpness *n* for each NOT gate in active growth and growth arrest, derived from three biological replicates measured on three different days. Dots represent medians, and each distribution is scaled to a constant width that marks the size of the dCas9 binding footprint on the genome. The location of the T7 promoter sequence is marked by the green shaded region. (**c**) Shifts in median parameter values from active growth to growth arrest for all repression curve parameters for all NOT gates, plotted against genomic location as in (**b**). The β shift is omitted for gates with no repression in a growth condition, as β = 0 when the fold repression is 1.

We first checked whether, under active growth conditions, the observed fold repression (β + α)/α followed the trends expected from existing literature (Figure 4b, left). Gates where dCas9 bound upstream of the T7 promoter exhibited low levels of repression, while gates where dCas9 bound on or immediately downstream of the promoter showed strong repression (up to a maximum of 116-fold from Gate D^T^_25_). In contrast, binding positions further downstream showed very low levels of repression, with only Gate D^C^_160_ repressing at a similar level to the upstream-binding gates. This stronger repression from Gate D^C^_160_ is consistent with previous observations that dCas9 blocks transcriptional elongation more strongly when bound to the complement strand.^22^ Interestingly, Gate D^T^_43_ exhibited no detectable repression, despite binding only 18 bp downstream, and on the same strand, from the most repressive gate D^T^_25_ (Supplemental Figure 7).

Overall, our results suggest that dCas9 is a better repressor when blocking transcriptional activation than elongation, which goes against the results of prior work on native *E. coli* promoters^22,28^ but is consistent with prior results on the T7 promoter.^29^ Our data therefore further support the notion that dCas9’s interactions with the T7 RNAP may differ significantly from those with *E. coli*’s native RNAP.

Both the Hill coefficient *n* and the sensitivity *K* were fairly consistent across gates, with median *n* values ranging between 1.2 and 2.1 and median *K* values spanning a 2.6-fold range across the gates. Gates where the repressor blocked transcriptional elongation had a slightly lower sensitivity (i.e., higher *K*) to repressor concentration (Figure 4b, left middle panel). The robustness of the Hill coefficient *n* to changes in the binding position is expected, as *n* is thought to be a protein-intrinsic property. The fact that *K* values are higher for more downstream binding gates, however, suggests that dCas9 may require higher concentrations to repress the T7 promoter via blocking transcriptional elongation than via blocking transcriptional activation.

### Impact of Growth Arrest on Repression Curve Properties

We next characterized the properties of repression curves for these same gates under growth arrest to understand how these properties vary between the two growth conditions (Figure 4b, right). Overall, repression values were much lower under growth arrest, reaching a maximum of 6.6-fold repression. This is likely due in large part to the reduction in the overall possible dynamic range for the gates under growth arrest due to the decrease in P_T7_’s ON level (Figure 3d). Position-dependent effects on repression strength were also homogenized under growth arrest, with upstream-binding and elongation-blocking gates able to reach similar levels of repression to the gates where dCas9 bound on or immediately downstream of the T7 promoter. Values of *K* and *n* also became more similar between gates under growth arrest, with median *K* values now spanning only a 1.4-fold range and median *n* values lying between 1.2 and 1.3.

Interestingly, Gate D^T^_43_ exhibited slight but detectable repression (1.7-fold) in growth arrest, despite exhibiting no detectable repression in active growth (Figure 4b, Supporting Information Figure 7). This result hints at the possibility that growth arrest, even though it decreases the level of maximal achievable repression, may impose physiological changes that broaden the capacity to repress across different repression schemes. One way in which this might occur is that differences in nucleoid organization between growth phases^30^ could cause short-range DNA interactions that prevented dCas9 from binding to the +43 position during active growth to be weakened under growth arrest.

Further analyzing the changes in repression curve properties at the individual gate level, we found that all gates exhibited a significant drop in overall expression level, with β and α decreasing by a geometric mean of 321- and 99-fold, respectively (Figure 4c). Depending on whether α decreased more, or less, than β, however, this led to either an increase or decrease in the overall repression strength, with the six gates binding closest to the promoter experiencing a reduction in repression strength (geometric mean of 7.9-fold reduction) and the remaining six gates experiencing minimal changes that averaged to a slight increase in repression strength (geometric mean of 1.3-fold increase). Changes to *K* and *n* were minimal across all gates, with no gate shifting these parameters more than 2.2-fold (geometric mean increase in median *K* and *n* was 1.3- and 1.2-fold, respectively).

These results, taken together, suggest that the impact of growth arrest on repression curves is almost entirely dominated by a drop in overall expression levels, captured by the decreases in β and α, with a moderate increase in gene expression noise and minimal changes to the sensitivity and sharpness of the curve.

### Generalization of Results to Other Repressors

We next asked whether the previous conclusions were specific to dCas9-mediated repression or whether they might be generalized to NOT gates based on other repressors. To investigate this question, we turned to a previously published system where dCas9 is mutated to reduce its native capacity for DNA binding (generating dCas9*) and then fused to the TetR-family transcriptional repressor PhlF.^31^ The resulting protein requires both a PhlF operator site (PhlO) and a PAM-targeting gRNA in order to bind to the DNA and acts as an intermediate condition between dCas9-mediated repression and PhlF-mediated repression.

We identified 3 candidate positions where a PhlO sequence could be inserted in the regulatory region of our design (before the promoter, before RiboJ, and before the ribosome binding site) near a PAM site and inserted the PhlO sequences in both possible orientations to create a set of 6 repressible constructs. We added spacer sequences as necessary to implement the previously determined optimal 12 bp spacing between the PhlO site and the PAM site^31^ to ensure that these constructs could be repressed by either the dCas9*:PhlF fusion or by PhlF alone, thus creating a new set of 12 NOT gates (Figure 5a). We label the fusion and PhlF gates as F^{T/C}^_N_ and P^{T/C}^_N_, respectively, following the previous convention. For P gates, N indicates the position of the midpoint of the PhlO site, while for F gates, N indicates the midpoint of the 66 bp window that spans both the dCas9 footprint and the PhlO site.

**Figure 5:**
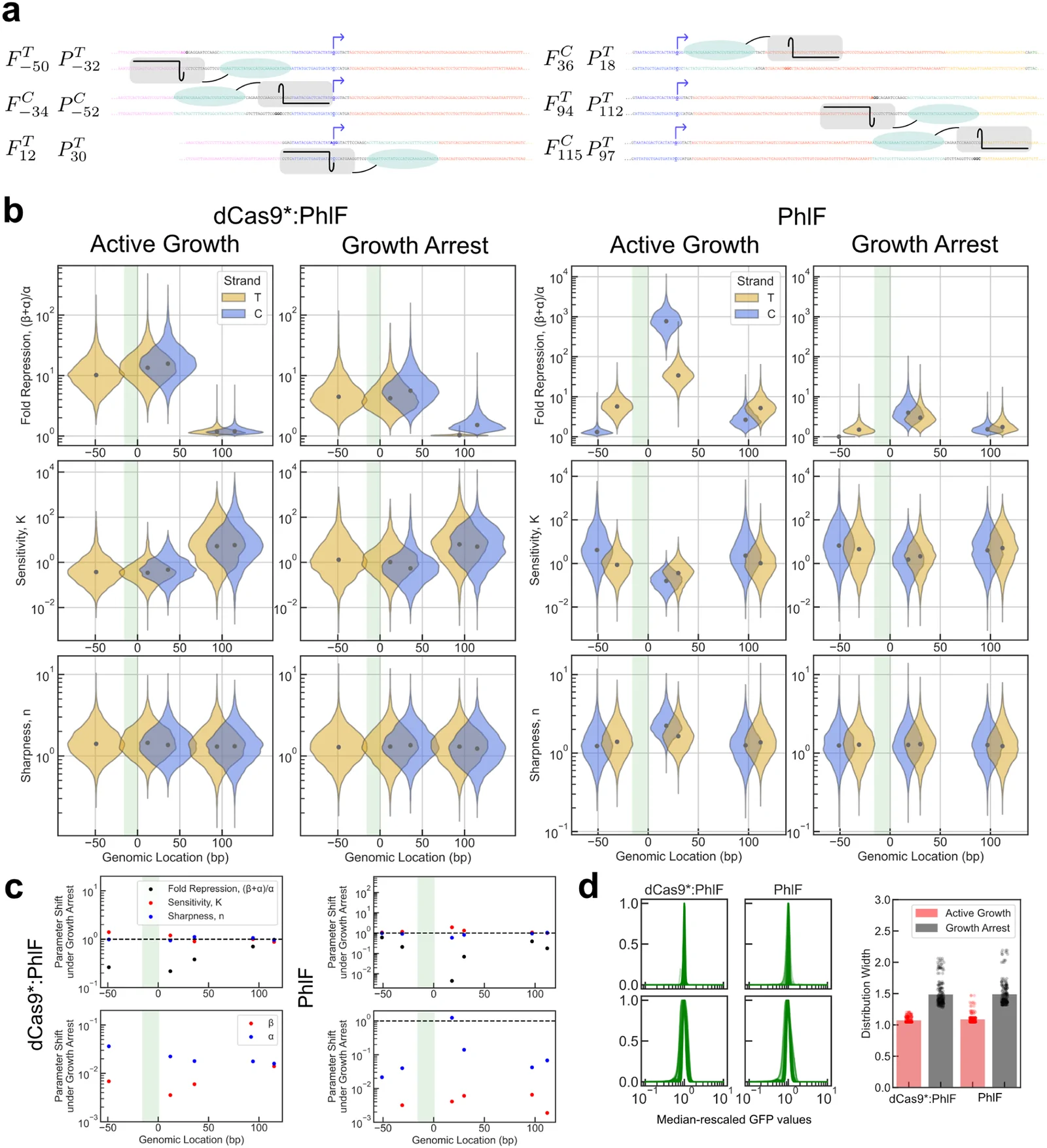
Measurement of repression curves from NOT gates driven by alternative repressors. (**a**) Schematic of repressed constructs. Genetic sequences are colored as in Figure 1e, with the PhlO site added in green. The +1 transcriptional start site is bolded, underlined, and marked with a hooked arrow, and PAM sequences are bolded. Each of the six repressible constructs generates two different NOT gates depending on whether it is repressed by the dCas9*:PhlF fusion or by PhlF alone. (**b**) Posterior distributions for repression curve parameters in active growth and growth arrest for the dCas9*:PhlF and PhlF NOT gates, derived from 3 biological replicates measured on 3 different days. Full repression curve measurements and MCMC outputs are shown in Supplemental Figures 14–24. Distribution widths are scaled to a constant value that indicates the width of the repressor’s genetic footprint. The location of the T7 promoter sequence is indicated by the green shaded region. Dots represent the median of the distribution. (**c**) Shifts in median parameter values from active growth to growth arrest for all repression curve parameters for all gates, plotted against genomic location as in (**b**). The same parameters are plotted across graphs within a row. The β shift is omitted for gates with no repression in a growth condition, as β = 0 when the fold repression is 1. (**d**) Rescaled and normalized kernel density estimates (KDEs) of GFP values across all induced conditions (left) and widths of these KDEs defined by the width of the crossing at a value of 0.2 (right). Bars represent the geometric mean.

We fused mScarlet3 to the C-terminus of dCas9*:PhlF and PhlF and measured their repression curves using the same methodology that we previously applied to the dCas9 gates. Data from gate F^C^_–34_ were thrown out after a later investigation revealed that the gRNA was assembled incorrectly, leaving repression curve data for 11 additional NOT gates.

We first assessed whether these new sets of gates followed the same trends relating the repressor binding location to properties of the repression curve as were observed for the dCas9 gates under active growth (Figure 5b). In both sets of gates, repression was strongest when the repressor bound immediately downstream of the +1 site (15-fold repression for Gate F^T^_12_, 865-fold repression for Gate P^T^_18_), although in the fusion gate library, the gate that bound upstream of the T7 promoter (Gate F^T^_–50_) exhibited comparable levels of repression. We note that, in our hands, dCas9*:PhlF achieved lower levels of repression than were originally reported (15-fold maximum versus ∼50-fold repression in ref 31). This discrepancy may be due to the fact that we used a full-length dCas9* protein in our fusion to keep the repressor identity more similar to our previous dCas9 gates, while the original study replaced dCas9’s HNH domain with a GGGSx2 linker.

The sensitivity, *K*, varied across only a 1.6- to 2.1-fold range for the fusion and PhlF gates, while the Hill coefficient *n* only ranged between 1.2 and 1.4 for the fusion gates and 1.3 and 2.1 for the PhlF gates. Overall, we concluded that the general relationship between the repressor binding location and NOT gate behavior was similar in these gates as it was for the dCas9-based gates.

We then assessed whether the repression curves from these gates also shifted in response to growth arrest in a similar way to the dCas9 gates (Figure 5c). As before, both the fusion and PhlF gates experienced a large decrease in overall expression levels, with median β values decreasing by a geometric mean of 149- and 253-fold and median α values decreasing by a geometric mean of 46- and 8.7-fold for the fusion and PhlF gates, respectively. *K* and *n*, as during active growth, exhibited minimal changes during growth arrest, with median *K* values increasing 1.1- and 1.3-fold and median *n* values increasing 1.0- and 1.2-fold across the fusion and PhlF gates, respectively. Interestingly, none of the tested gates exhibited notable multimodality in either growth condition, although the average widths of the GFP distributions under growth arrest were still wider than under active growth for both sets of gates by 1.4-fold (Figure 5d).

Taken together, these trends are broadly consistent with those that we observed in the dCas9-based NOT gate library, suggesting that the impacts of growth arrest on the repression curves observed in this work may generalize to other repressors with different molecular implementations.

## Discussion

We have presented a systematic characterization of engineered genetic circuit components that directly compares their performance under active growth and growth arrest conditions. Doing so required the development of specialized NOT gate architectures and novel measurement approaches to quantitatively characterize repression curves in a way that is compatible with low gene expression levels and cell-to-cell variability. After measuring the performance of 23 different NOT gates based on three different repressors, we find that the impact of growth arrest on repression curves is dominated by a single effect, the significant decrease in output expression levels by 2 orders of magnitude. The consistent nature of this effect across all tested repressors suggests that it is likely driven by a globally reduced gene expression capacity under growth arrest. All other curve parameters, meanwhile, either experienced minor changes (such as the moderate increase in gene expression noise) or were essentially unaffected (as were the sensitivity and sharpness of the curves).

Because the decrease in expression strength tended to occur asymmetrically, with unrepressed expression levels (driven by β) decreasing more than fully repressed expression levels (driven by α), most NOT gates experienced a decrease in overall repression strength under growth arrest. However, in several cases, we found that individual gates were able to maintain similar fold repression values between the two growth conditions, despite the fact that their absolute expression levels still decreased in growth arrest. For example, even though Gate D^C^_–23_ experienced a 309-fold reduction in β and a 146-fold reduction in α, its overall fold change only decreased by 1.3-fold (going from 8.5-fold repression in active growth to 6.3-fold repression under growth arrest), while its sensitivity and sharpness experienced similarly small changes (1.3-fold and 1.2-fold increases in *K* and *n*) (Figure 4b,c). This means that the repression curve of this gate almost completely preserved its shape and simply shifted downward on the input/output plot as a consequence of growth arrest.

While our assay provides a general approach to quantitatively measuring the repression curve and its associated parameters for various repressors, we note that some features of our assay limit its direct generalizability to broader studies of transcriptional repression. First, the fact that we introduce a fluorescent protein fusion to our repressor means that specific parameter values measured through this approach may not necessarily hold for the native versions of these proteins. Fluorescent protein fusions have been known to affect functional properties of proteins, such as subcellular localization.^32^ However, it is nonetheless the case that the use of C-terminal fluorescent fusions to transcription factors is widespread and has provided useful insights into transcriptional regulation.^33^ Second, our assay samples points along the repression curve by setting repressor concentrations at values determined by the activity of an inducible promoter driven by externally supplied concentrations of the inducer. This inducible promoter is itself affected by the global physiological changes associated with different growth conditions, meaning that it is challenging to set the repressor concentration to precisely defined values that would more accurately elucidate the specific repression curve parameters. Third, our assay provides only phenomenological observations that do not directly reveal any mechanistic explanations. We can speculate, for example, that the consistently observed increase in gene expression noise through the widening of the GFP distribution might be due to changes in protein degradation dynamics under growth arrest, but without targeted follow-up work, we cannot obtain any further insights on this point. Despite these limitations, however, our study shows that our approach provides a viable framework for quantitatively investigating transcriptional repression dynamics under different growth conditions.

### Implications for Genetic Circuit Design

Having shown that growth arrest leads to a dramatic reduction in the expression level of repression curves, we can now ask what the implications of these results are for the engineering of larger, more complex genetic circuits that can function under growth arrest. Recall that layered repression circuits require their component repression curves to align, which means that the sensitivity of the downstream repressor must lie within the dynamic range of the upstream repressor (α_1_ < *K*_2_ < β_1_ + α_1_) and that our data show that *K* values change minimally, while β and α values can decrease significantly under growth arrest. This means that growth arrest introduces a general mechanism for layered circuit failure by causing the β value of the upstream repressor to drop below the value of *K* for the downstream repressor. Even the strongest individual repressors lead to only ∼200-fold repression in a genetic circuit context,^18^ while the average reduction in β across all NOT gates measured in our study was around 285. The magnitude of the expression reduction under growth arrest is therefore of a greater magnitude than the dynamic range of an individual repressor, meaning that any two repression curves will likely be misaligned under growth arrest. We illustrate this in Figure 6 with a NOT–NOT circuit composed of the two strongest NOT gates from a library of 73 genomically mined repressors.^18^ We can see that the reduction in expression level is strong enough to fully negate the predicted responsiveness of the circuit under growth arrest. Under conditions where the circuit responds to endogenous signals, which are also likely to have reduced magnitudes under growth arrest, this effect would only be further exacerbated if the initial input values do not capture the full dynamic range of the first repression curve.

**Figure 6:**
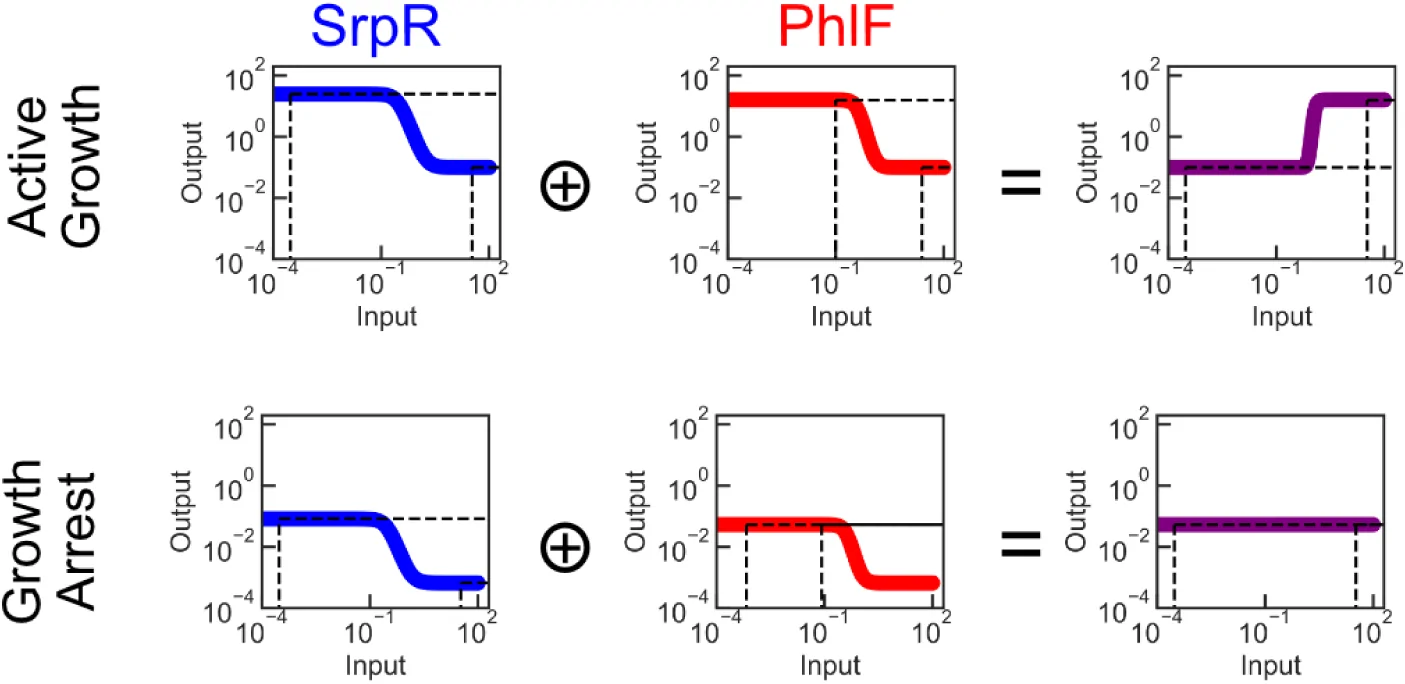
Illustration of the predicted impacts of growth arrest on circuit function. Predicted performance of a NOT–NOT circuit built from the TetR family repressors SrpR and PhlF. Although the circuit is in an always-ON failure mode under growth arrest, the decrease in overall expression levels means that its performance is nearly identical to that of an always-OFF failure mode in active growth. Repression curve parameters for active growth were obtained from ref. 18, and growth arrest curves were generated by scaling the active growth β and α values by 1/300 and 1/150, respectively, following the behavior of Gate D^C^_–23_. All values are in Relative Expression Units (REUs).

Despite this conclusion, however, it is nonetheless the case that genetic circuits with nontrivial complexity have been validated to function in natural environments where growth arrest is expected to occur, like the gut or the soil.^34–36^ Our results provide a possible explanation for this discrepancy. In the NOT–NOT circuit described above, the misalignment of the repression curves under growth arrest leads to an always ON behavior in the full circuit. However, because the level of the ON state under growth arrest is over 100-fold lower than it is in active growth, this state is functionally indistinguishable from an OFF state in active growth. This serendipitous alignment of effects means that growth-arrested cells would appear as false negatives for circuit function and not impede the measured signal from the subset of cells in the population that are actively growing and exhibiting proper circuit function. Indeed, single-cell measurements of genetic circuit performance in natural environments have shown a high proportion of nonresponsive cells even when genetic analyses confirm that they contain the circuit.^37^

Taken together, these results show that individual repression curves still maintain the ability to repress under growth arrest and sometimes even to levels similar to those in active growth. However, the mismatch between the absolute expression levels of the repressor and the sensitivity of its cognate promoter means that repression curves will almost always be misaligned, and so genetic circuits built using layered repression architectures will fail to function. Nonetheless, in environments where cells transition between active growth and growth arrest, actively growing cells would still be able to transiently experience repression curve alignment and therefore exhibit proper circuit function. This means that population-level measurements will likely show a functional circuit response, albeit only appearing in a subset of the cells. However, in applications where cells must consistently perform circuit computations specifically under growth-arrested conditions, such as in long-term biomonitoring or embedded structural materials, new genetic design paradigms will be required.

These new design paradigms may not necessarily require a full rethinking of the fundamental architectural principles of genetic circuits, however. By implementing auxiliary strategies that realign layered repression curves, either by increasing expression levels or by increasing sensitivity, it may be possible to use the same architectures to achieve similar performance between active growth and growth arrest. In some cases, only a moderate correction may be needed to reattain circuit function. In the NOT–NOT circuit proposed above, for example, mitigating the reduction in expression level by only 8-fold (bringing the growth arrest-associated reductions in β and α to 38-fold and 19-fold, respectively) would allow the overall circuit to exhibit a 10-fold response range under growth arrest (Supplemental Figure 25).

One example of a potential strategy would be to make use of the differential copy number of the genes in the circuit. Increasing the copy number of a repressed module would increase its output levels, but it would also decrease its sensitivity by requiring a higher total repressor concentration to fully repress the gene. For linear circuits like logic circuits, having the upstream repressors at a higher copy number than downstream repressors might therefore favor curve alignment. While this strategy may not scale to large circuits and would likely not apply to feedback circuits like memory switches or oscillators, it would still be a promising area of research to investigate how growth arrest modulates the impact of copy number on repression curve properties.

Another more general approach would be to extend the expected time scale of circuit function so that output proteins from each circuit layer are given a longer time to accumulate. A previous study has shown that exogenously induced proteins can accumulate linearly in stationary phase *E. coli* cells over a period of at least 24 h.^5^ If this effect persists over multiple days, then enough protein might accumulate to mitigate the reduction in β, allowing gates to be wired together and retain their expected performance even under growth arrest. Under such a scheme, however, new strategies to resolve the challenges associated with circuit computation over weeklong time scales would need to be developed, like solutions for increased leak and alternative mechanisms of protein removal.

Finally, we note that natural systems may also provide a valuable source of inspiration for new design strategies for engineering genetic circuits under growth arrest. Bacteria still engage in gene regulation under growth arrest,^38^ and looking further into the gene regulatory architectures that are involved in such pathways may reveal new insights about how they have evolved to maintain layered regulation under these conditions, or whether they have adapted fundamentally different regulatory architectures for these needs.

Synthetic biology holds great promise for transforming the physical world in ways that can advance both human and ecological welfare, but doing so will require the development of new sets of design principles to allow the safe and reliable performance of engineered biosolutions in real-world environments. This work takes a first step toward this goal by characterizing the impacts of microbial growth arrest on genetic circuit behavior to reveal which performance parameters are most affected, revealing new avenues for future research to address these challenges.

## Methods

### Construction of Genetic Circuits

All circuits were constructed using 3G Assembly^39^ using parts from the CIDAR MoClo Extension Part Kit,^40^ with the exception of custom gRNAs and fusion proteins created for this work (see the following section). NOT gates were assembled in two cassettes: one contained P_LacO_-driven T7 RNA polymerase and P_T7_-driven sfGFP and the other contained P_Tet_-driven repressor:mScarlet3 fusion protein and P_OR1OR2_-driven gRNA. The two cassettes were integrated into the Lambda and P21 integration sites, respectively, of the *E. coli* genome using the pOSIP clonetegration system plasmids KL and CT.^41^ The resulting strains are kanamycin- and chloramphenicol-resistant. The background strain for all experiments was *E. coli* Marionette MG1655,^26^ which natively expresses the LacI and TetR repressors that regulate P_LacO_ and P_Tet_, respectively. The existing chloramphenicol resistance gene in the Marionette strain was excised prior to circuit insertion using the temperature-sensitive plasmid pE-FLP,^41^ which was then cured by repeated passaging of the strain at 37 °C. Sequences of all circuit components are provided in Supporting Information Table 1.

For gates where the PhlO sequence was inserted between genetic parts of the P_T7_-driven sfGFP cassette in various locations and orientations, the relevant portion of the cassette was commercially synthesized as a dsDNA fragment flanked by BsaI cut sites and used in lieu of the relevant part plasmids in the Golden Gate step of 3G assembly. All subsequent construction steps proceeded, as described above.

### Construction of gRNAs and Fusion Proteins

gRNAs were constructed as part of plasmids in the CIDAR MoClo format^42^ with a Hammerhead ribozyme upstream of the gRNA sequence to standardize the 5′ sequence of the resulting RNA. Different gRNA variants were constructed by PCR amplifying the gRNA part plasmid into two segments that exclude the 20 bp variable sequence using custom primers containing the new 20 bp variable sequence as overhangs and religating the plasmid using Gibson assembly.

Repressor:mScarlet3 fusion proteins were constructed as part of plasmids in the CIDAR MoClo format by using Gibson assembly to combine PCR amplicons of the mScarlet3 part plasmid (without the start codon) and the dCas9 or PhlF part plasmids (without the stop codon) with a 2x GGGS linker as the overhang. mScarlet3 was always attached to the C-terminus of the repressor.

The dCas9*:PhlF fusion protein was created by commercially synthesizing a dsDNA fragment containing the modified region of dCas9 with overhangs to PCR amplicons of the dCas9 and PhlF:mScarlet3 part plasmids and using Gibson assembly to combine the fragments into a full dCas9*:PhlF:mScarlet3 part plasmid in the CIDAR MoClo format.

### Growth Media Design

Carbon-limited media was created by first preparing a stock of freshwater base (100g NaCl, 40 g MgCl_2_·6H_2_O, 10g CaCl_2_·2H_2_O, 50 g KCl in 1 L water ), a stock of 0.5 M NH_4_Cl to act as a nitrogen source, a stock of 1 M Na_2_SO_4_ to act as a sulfur source, and a stock of 100 mM KH_2_PO_4_ at pH 7.2 to act as a phosphorus source. To create 1 L of carbon-limited media, 10 mL of freshwater base, 10 mL of the nitrogen source, 250 μL of the sulfur source, and 1 mL of the phosphorus source were combined with one 10 mL vial of Trace Mineral Supplement (ATCC MD-TMS) and added to Milli-Q water to a total volume of 1 L. The resulting mixture was then filter-sterilized.

For active growth conditions, commercial M9CA minimal media (Teknova M8010) was used, which contains 1% glucose, 0.1% casamino acids, 0.5 μg/mL thiamine, 0.2 mM magnesium sulfate, and 0.1 mM calcium chloride at pH 7.0.

### Strain Growth and Induction Conditions

Glycerol stocks of engineered strains were streaked onto LB agar plates with appropriate selection markers and grown overnight at 37 °C. One colony from each strain was then picked and inoculated into 3 mL M9CA media with antibiotic selection (25 μg/mL kanamycin and 17 μg/mL chloramphenicol) and grown for 24 h in a 15 mL tube in a shaking incubator at 30 °C and 250 rpm to generate a stationary phase culture, which we will hereafter refer to as the “overnight culture”. The optical density (OD600) values of the overnight culture for each strain were then measured with a spectrophotometer. At this point, each overnight culture is split into two parallel protocols, which are handled simultaneously, but we will describe each one to completion separately.

In the first protocol, which maintains the cells in active growth, fresh batches of M9CA media with selective antibiotics were prepared in 96-well deep-well culture plates (NEST prod. no. 502062) with 12 wells of 500 μL per strain. Eleven of these wells were used to titrate anhydrotetracycline (atc; Sigma-Aldrich, cat. no. 37919) concentrations to induce P_Tet_-driven expression of the repressor via final concentrations spanning 0 to 200 ng/mL (see Supplemental Figures 1–24 for specific concentrations used for each experimental condition). The overnight cultures were diluted 1:10,000 into these wells, which were then returned to the shaking incubator covered with a Breathe-Easier Sealing Film (Diversified Biotech BERM-2000).

From this point, in order to maintain active growth, each well was diluted 1:1,000 every 12 h by directly transferring 0.5 μL of culture into 499.5 μL of fresh M9CA media containing antibiotic and inducer concentrations at the same values as in the original well. On the second such dilution (i.e., 24 h after the initial dilution of the overnight culture), 1 mM IPTG (Sigma-Aldrich, cat. no. 420322) was added to the 11 atc-induced wells. Subsequent dilutions therefore also included 1 mM IPTG in the fresh media for these wells to maintain inducer concentration. 24 h after IPTG induction (i.e., 48 h after the initial dilution of the overnight culture), the M9CA cultures were transferred into 96-well polystyrene microplates (Corning prod. no. 3370) via a 1:400 dilution into 200 μL of fresh M9CA media with no antibiotics or inducers, and all cultures were immediately measured via flow cytometry.

We now describe the second parallel protocol, which maintains cells in growth arrest. To minimize the carryover of residual nutrients from the original growth media, 1 mL of the overnight culture for each strain was transferred into 1.5 mL Eppendorf tubes and spun down at 1,377 g on a tabletop centrifuge for 10 min. The supernatant was removed and resuspended in 1 mL of carbon-limited media, and the spin procedure was repeated. The supernatant was again removed and replaced with 1 mL of fresh carbon-limited media. The resulting solutions were then rediluted into 7 mL of carbon-limited media to bring the final density of each culture to an OD600 of 0.0025. The dilute cultures were then split into 12 200 μL aliquots per strain in 96-well polystyrene microplates, and 11 of these wells were induced with varying concentrations of atc. Antibiotics were not added to the carbon-limited media to avoid adding potential additional stressors to the cells. The plate was then returned to the shaking incubator under the same conditions as above. All cultures were grown for 24 h, after which IPTG was directly added to the 11 atc-induced wells to a final concentration of 1 mM, and the cultures were returned to the shaking incubator. 24 h after the IPTG induction (i.e., 48 h after the initial wash of the overnight culture into carbon-limited media), the culture plates were removed from the shaking incubator and directly measured by flow cytometry.

We note that the two parallel protocols finish at the same time, meaning that the flow cytometry data for the two growth conditions were obtained in the same measurement session.

### Flow Cytometry

Cells were measured on a Cytoflex S flow cytometer. Forward and side scatter thresholds to detect bacteria, as opposed to spurious debris or instrument noise, were determined manually based on comparisons to readings from samples containing only media. sfGFP expression was measured using 488 nm excitation and a 525/40 nm bandpass filter, while mScarlet3 expression was measured using 561 nm excitation and a 610/20 nm bandpass filter. At the beginning of each measurement session, calibration beads (Spherotech RCP-30–5A) were measured to allow conversion of arbitrary fluorescent units into absolute fluorescence units (Molecules of Equivalent Fluorescein (MEFL) for sfGFP and Molecules of Equivalent PE-TexasRed (MEPETR) for mScarlet3) by fitting to an 8-point standard curve. M9CA cultures were measured until 50,000 putative bacterial events were observed, while carbon-limited cultures were measured for 1 min at the maximal standard flow rate of 60 μL/min, which typically yielded 5–10,000 putative bacterial events per sample.

### Analysis of Flow Cytometry Data

Flow cytometry data were analyzed using the Python package FlowCal.^43^ Fluorescence values were converted into absolute units using the appropriate bead data from each run using FlowCal’s built-in calibration functions. We then performed density gating on each set of measurements to keep only the events that were in the densest region of the forward scatter/side scatter plot in order to better exclude nonsinglet events from our analysis. We used a density threshold of 0.4, meaning we discarded 60% of the measured events from each sample.

### Noise Analysis and Determination of Nonunimodality

In order to compare the distributions of sfGFP fluorescence values from each sample across different conditions, we performed the following normalization procedure. For a given sample, we extracted the measured GFP values, log-transformed them, and divided each resulting value by the median of the distribution. We then generated a kernel density estimate (KDE) from these values using the scipy.stats.gaussian_kde­() function^44^ with default parameters and then standardized the height of the KDE by normalizing it to a height of 1. This procedure allowed us to overlay all GFP distributions from all conditions on top of each other to analyze the shape and width of the distributions. Distribution widths were calculated by finding the first and last points at which the KDE intersects a value of 0.2 and dividing these points to obtain the width of this distance in logarithmic space.

Distributions were classified as “notably nonunimodal” if their derivative was negative at any point below the median (which is rescaled to be one). Buffer values of 0.1 below the median and 0.005 below zero for the derivatives were applied to accommodate noise in the data.

### Bayesian Parameter Estimation

Markov Chain Monte Carlo (MCMC) was implemented using the Python package emcee.^45^ Priors for each of the parameters in the Hill repression function (eq 1) were set as log-normal distributions with scale and shape parameters μ_α_ = *y*_low_, σ_α_ = 1, μ_β_ = *y*_high_ – *y*_low_, σ_β_ =0.5, μ_K_ = 0.1, σ_K_ = 1, μ_*n*_ = 1, σ_*n*_ = 0.5, where *y*_high_ is the geometric mean of the median unrepressed GFP values from each replicate and *y*_low_ is the geometric mean of the median maximally repressed GFP values from each replicate. Median (RFP, GFP) fluorescence values from each measured sample were extracted, and the values from all induction conditions and replicates were pooled together for each gate to generate 11 × 3 = 33 data points along the repression curve for each gate. MCMC was run with 32 walkers for 10,000 iterations, fitting the data against the Hill repression function. Autocorrelation analysis was used to assess convergence by confirming that the maximal autocorrelation value τ_max_ was less than the number of iterations divided by 50.

## Supplementary Material



## Data Availability

All raw data from this manuscript are uploaded to the Zenodo repository accessible at doi: 10.5281/zenodo.18415279 and code to reproduce all analyses and graphs in this work is available at https://github.com/jpmarken/GrowthArrestRepression

## Notes

Supplemental figures and genetic construct sequences (PDF)

## #

Section of Microbiology, University of Copenhagen, Universitetsparken 15, DK-2100, Copenhagen, Denmark
