# Supplementary material for: Quantitative Measurement of Synthetic Repression Curves Reveals Design Challenges for Genetic Circuit Engineering under Growth Arrest
Source: ACS Synth Biol. 2026 May 13;15(8):3235–48. doi: 10.1021/acssynbio.6c00123 (PMC13505334; doi:10.1021/acssynbio.6c00123)
Supplement: Supplementary file 1 [file sb6c00123_si_001.pdf]

Supporting Information for:

Quantitative measurement of synthetic repression curves reveals design challenges for genetic circuit engineering under growth arrest

John P. Marken[†]*, Mark L. Prator, Bruce A. Hay, Richard M. Murray

Division of Biology and Biological Engineering, California Institute of Technology,
1200 E California Blvd, Pasadena, CA, USA 91125

[†] Present address: Section of Microbiology, University of Copenhagen,
Universitetsparken 15, DK-2100, Copenhagen, Denmark

* Corresponding author: john.marken@bio.ku.dk

Contents

Supplemental Figures 2

Genetic Construct Sequences 27

Supplemental Figures

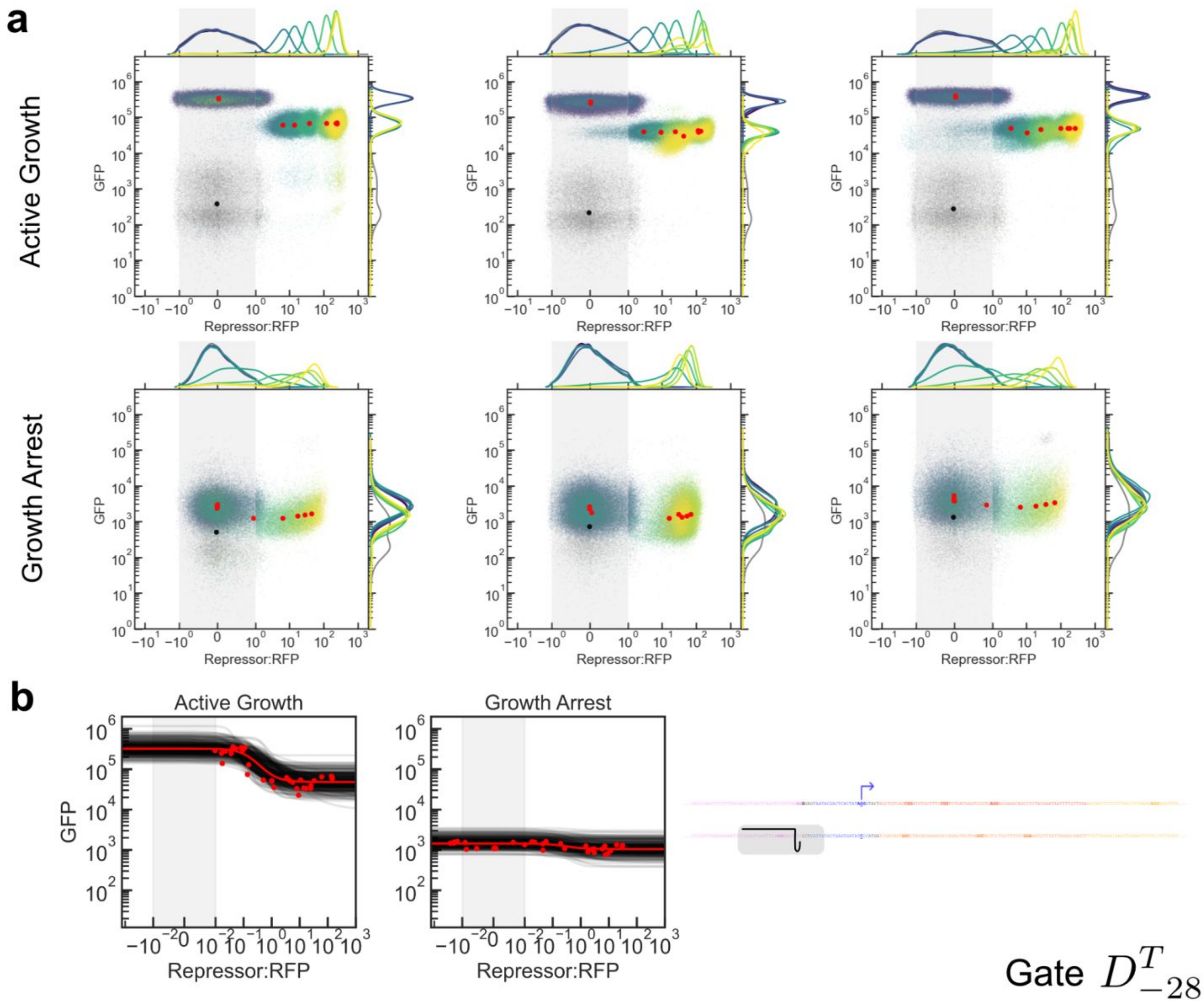

Supplemental Fig. 1: *Repression curves for gate DT-28. (**a**) Flow cytometry data. Three biological replicates (columns) were measured on three different days. Dots represent medians of distributions for each induction condition, and colors represent atc concentrations. Induction conditions for atc were: Active Growth, Replicate 1: 0, 0.02, 0.06, 0.2, 0.43, 0.93, 2, 6, 20, 60, 200 ng/mL. Active Growth, Replicate 2: 0, 0.02, 0.2, 0.43, 0.93, 2, 3.5, 6.3, 11.3, 20, 200 ng/mL. Active Growth, Replicate 3: 0, 0.2, 0.43, 0.93, 2, 2.6, 3.5, 6.3, 11.3, 20, 200 ng/mL. Growth Arrest, all replicates: 0, 0.02, 0.06, 0.2, 0.43, 0.93, 2, 6, 20, 60, 200 ng/mL. Grey points indicate cultures that were not induced with either IPTG or atc. Data are plotted on a symmetric log scale where the grey shaded region is linear. (**b**) 300 trajectories taken from the end of the MCMC process overlaid against the median (RFP,GFP) values from all three replicates.*

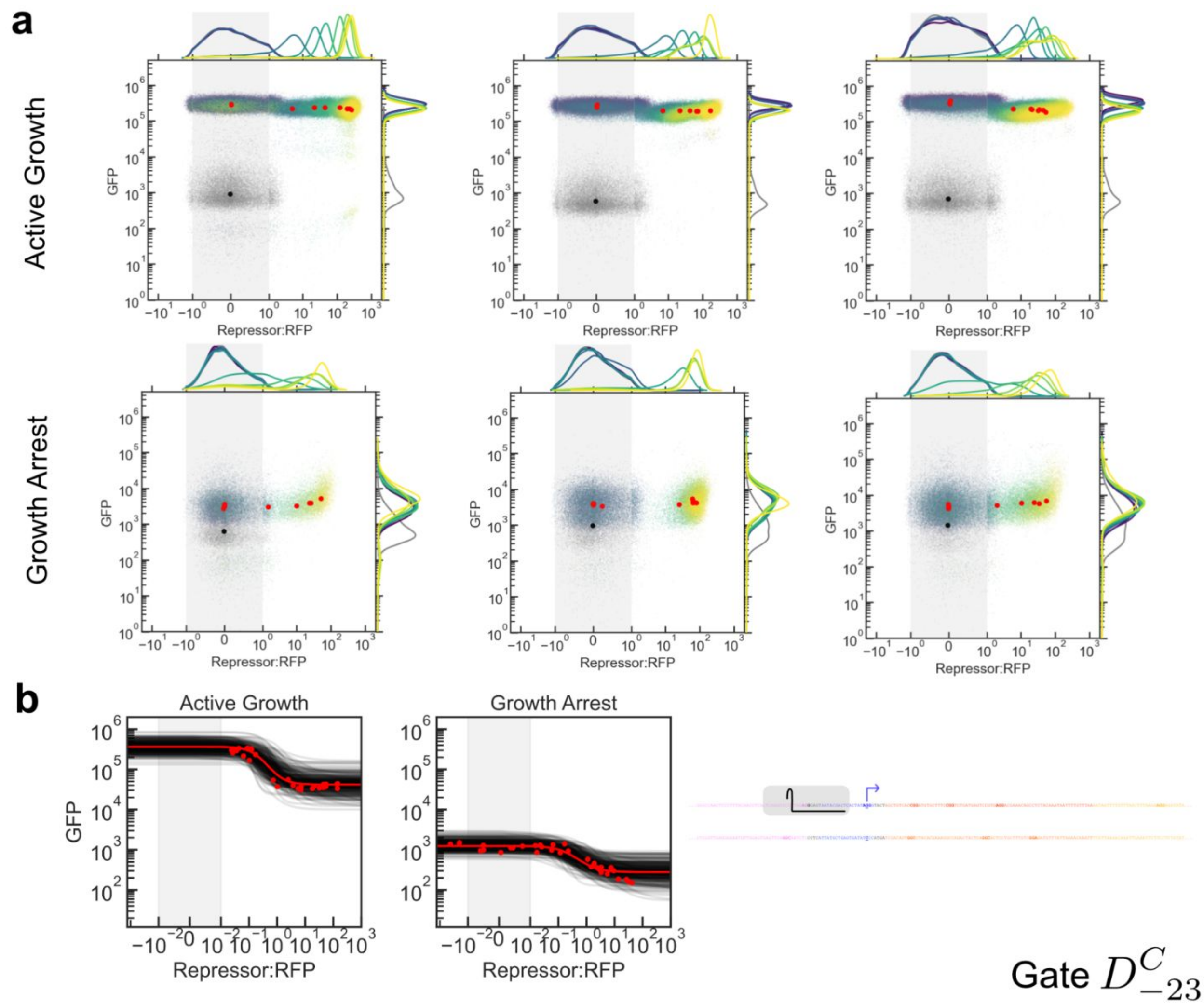

Supplemental Fig. 2: *Repression curves for gate DC-23. (**a**) Flow cytometry data. Three biological replicates (columns) were measured on three different days. Dots represent medians of distributions for each induction condition, and colors represent atc concentrations. Induction conditions for atc were: Active Growth, Replicate 1: 0, 0.02, 0.06, 0.2, 0.43, 0.93, 2, 6, 20, 60, 200 ng/mL. Active Growth, Replicate 2: 0, 0.02, 0.2, 0.43, 0.93, 2, 3.5, 6.3, 11.3, 20, 200 ng/mL. Active Growth, Replicate 3: 0, 0.2, 0.43, 0.93, 2, 2.6, 3.5, 6.3, 11.3, 20, 200 ng/mL. Growth Arrest, all replicates: 0, 0.02, 0.06, 0.2, 0.43, 0.93, 2, 6, 20, 60, 200 ng/mL. Grey points indicate cultures that were not induced with either IPTG or atc. Data are plotted on a symmetric log scale where the grey shaded region is linear. (**b**) 300 trajectories taken from the end of the MCMC process overlaid against the median (RFP,GFP) values from all three replicates.*

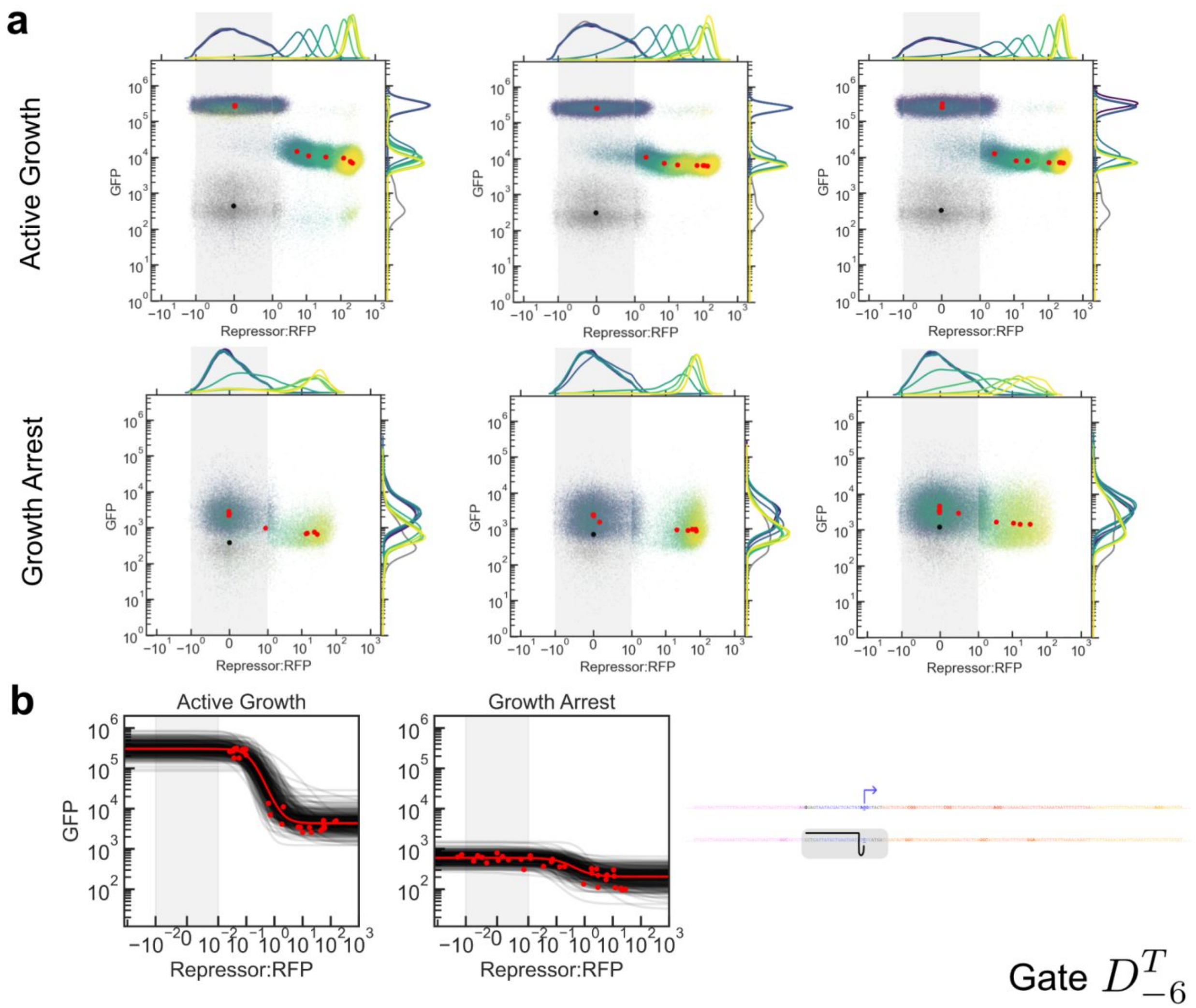

Supplemental Fig. 3: *Repression curves for gate D^{T}_{-6}. (**a**) Flow cytometry data. Three biological replicates (columns) were measured on three different days. Dots represent medians of distributions for each induction condition, and colors represent atc concentrations. Induction conditions for atc were: Active Growth, Replicate 1: 0, 0.02, 0.06, 0.2, 0.43, 0.93, 2, 6, 20, 60, 200 ng/mL. Active Growth, Replicate 2: 0, 0.02, 0.2, 0.43, 0.93, 2, 3.5, 6.3, 11.3, 20, 200 ng/mL. Active Growth, Replicate 3: 0, 0.2, 0.43, 0.93, 2, 2.6, 3.5, 6.3, 11.3, 20, 200 ng/mL. Growth Arrest, all replicates: 0, 0.02, 0.06, 0.2, 0.43, 0.93, 2, 6, 20, 60, 200 ng/mL. Grey points indicate cultures that were not induced with either IPTG or atc. Data are plotted on a symmetric log scale where the grey shaded region is linear. (**b**) 300 trajectories taken from the end of the MCMC process overlaid against the median (RFP,GFP) values from all three replicates.*

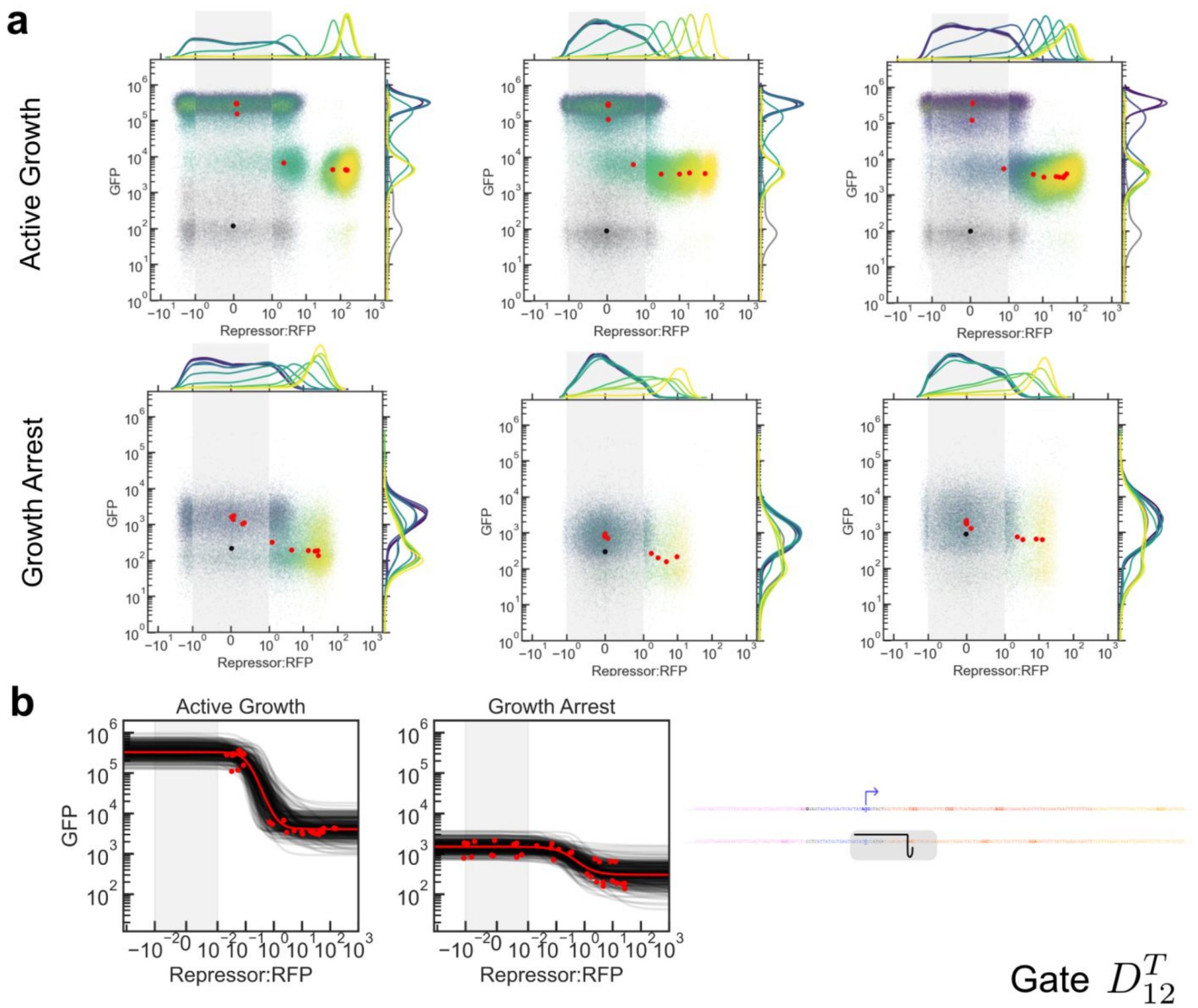

Supplemental Fig. 4: *Repression curves for gate D^T_{12}. (**a**) Flow cytometry data. Three biological replicates (columns) were measured on three different days. Dots represent medians of distributions for each induction condition, and colors represent atc concentrations. Induction conditions for atc were: Active Growth, Replicate 1: 0, 0.02, 0.06, 0.2, 0.43, 0.93, 2, 6, 20, 60, 200 ng/mL. Active Growth, Replicate 2: 0, 0.02, 0.2, 0.43, 0.93, 2, 3.5, 6.3, 11.3, 20, 200 ng/mL. Active Growth, Replicate 3: 0, 0.2, 0.43, 0.93, 2, 2.6, 3.5, 6.3, 11.3, 20, 200 ng/mL. Growth Arrest, all replicates: 0, 0.02, 0.06, 0.2, 0.43, 0.93, 2, 6, 20, 60, 200 ng/mL. Grey points indicate cultures that were not induced with either IPTG or atc. Data are plotted on a symmetric log scale where the grey shaded region is linear. (**b**) 300 trajectories taken from the end of the MCMC process overlaid against the median (RFP,GFP) values from all three replicates.*

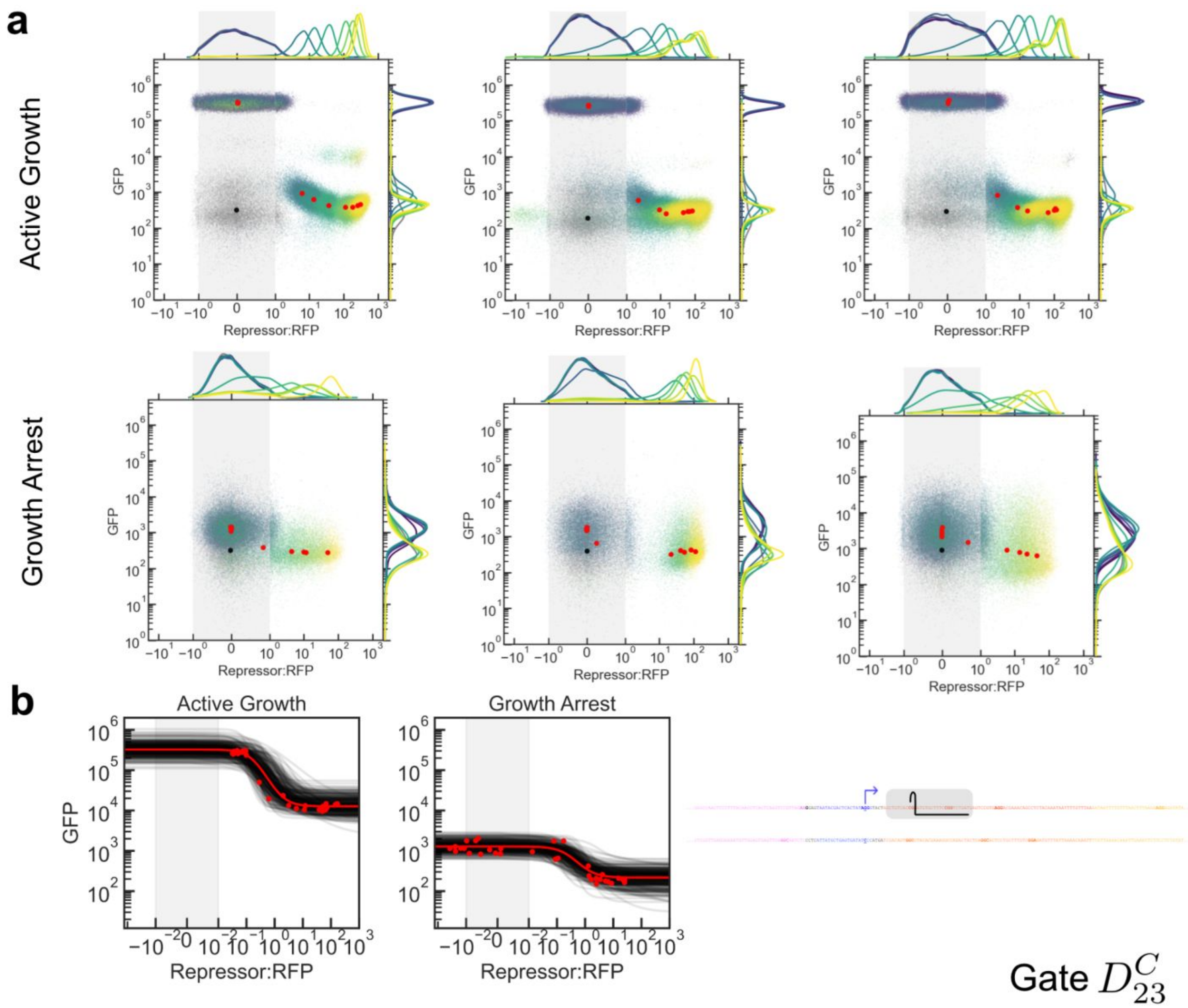

Supplemental Fig. 5: *Repression curves for gate D^{C}_{23}. (**a**) Flow cytometry data. Three biological replicates (columns) were measured on three different days. Dots represent medians of distributions for each induction condition, and colors represent atc concentrations. Induction conditions for atc were: Active Growth, Replicate 1: 0, 0.02, 0.06, 0.2, 0.43, 0.93, 2, 6, 20, 60, 200 ng/mL. Active Growth, Replicate 2: 0, 0.02, 0.2, 0.43, 0.93, 2, 3.5, 6.3, 11.3, 20, 200 ng/mL. Active Growth, Replicate 3: 0, 0.2, 0.43, 0.93, 2, 2.6, 3.5, 6.3, 11.3, 20, 200 ng/mL. Growth Arrest, all replicates: 0, 0.02, 0.06, 0.2, 0.43, 0.93, 2, 6, 20, 60, 200 ng/mL. Grey points indicate cultures that were not induced with either IPTG or atc. Data are plotted on a symmetric log scale where the grey shaded region is linear. (**b**) 300 trajectories taken from the end of the MCMC process overlaid against the median (RFP,GFP) values from all three replicates.*

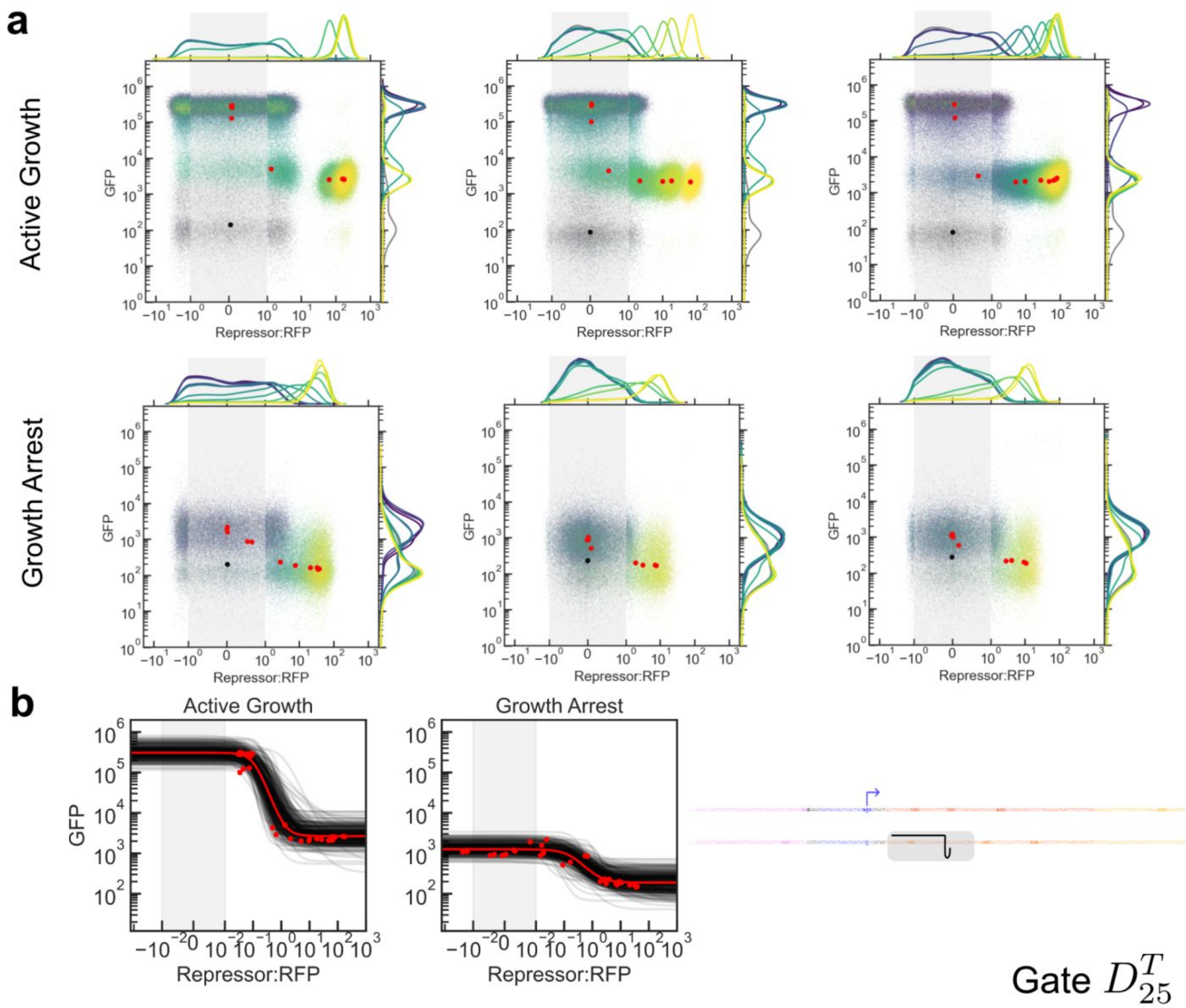

Supplemental Fig. 6: *Repression curves for gate $D^T{}_{25}$. (**a**) Flow cytometry data. Three biological replicates (columns) were measured on three different days. Dots represent medians of distributions for each induction condition, and colors represent atc concentrations. Induction conditions for atc were: Active Growth, Replicate 1: 0, 0.02, 0.06, 0.2, 0.43, 0.93, 2, 6, 20, 60, 200 ng/mL. Active Growth, Replicate 2: 0, 0.02, 0.2, 0.43, 0.93, 2, 3.5, 6.3, 11.3, 20, 200 ng/mL. Active Growth, Replicate 3: 0, 0.2, 0.43, 0.93, 2, 2.6, 3.5, 6.3, 11.3, 20, 200 ng/mL. Growth Arrest, all replicates: 0, 0.02, 0.06, 0.2, 0.43, 0.93, 2, 6, 20, 60, 200 ng/mL. Grey points indicate cultures that were not induced with either IPTG or atc. Data are plotted on a symmetric log scale where the grey shaded region is linear. (**b**) 300 trajectories taken from the end of the MCMC process overlaid against the median (RFP,GFP) values from all three replicates.*

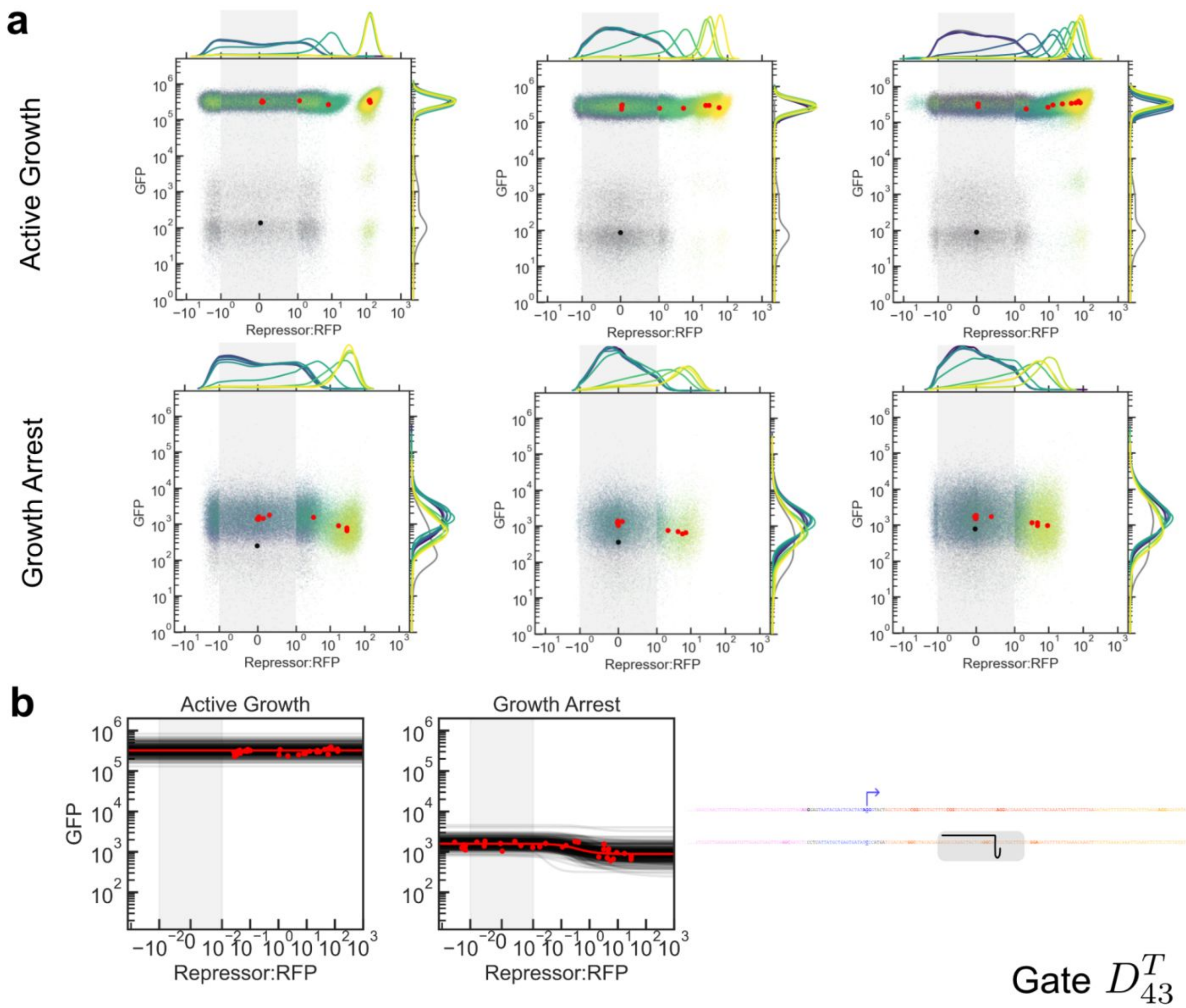

Supplemental Fig. 7: *Repression curves for gate $D^T{}_{43}$. (**a**) Flow cytometry data. Three biological replicates (columns) were measured on three different days. Dots represent medians of distributions for each induction condition, and colors represent atc concentrations. Induction conditions for atc were: Active Growth, Replicate 1: 0, 0.02, 0.06, 0.2, 0.43, 0.93, 2, 6, 20, 60, 200 ng/mL. Active Growth, Replicate 2: 0, 0.02, 0.2, 0.43, 0.93, 2, 3.5, 6.3, 11.3, 20, 200 ng/mL. Active Growth, Replicate 3: 0, 0.2, 0.43, 0.93, 2, 2.6, 3.5, 6.3, 11.3, 20, 200 ng/mL. Growth Arrest, all replicates: 0, 0.02, 0.06, 0.2, 0.43, 0.93, 2, 6, 20, 60, 200 ng/mL. Grey points indicate cultures that were not induced with either IPTG or atc. Data are plotted on a symmetric log scale where the grey shaded region is linear. (**b**) 300 trajectories taken from the end of the MCMC process overlaid against the median (RFP,GFP) values from all three replicates.*

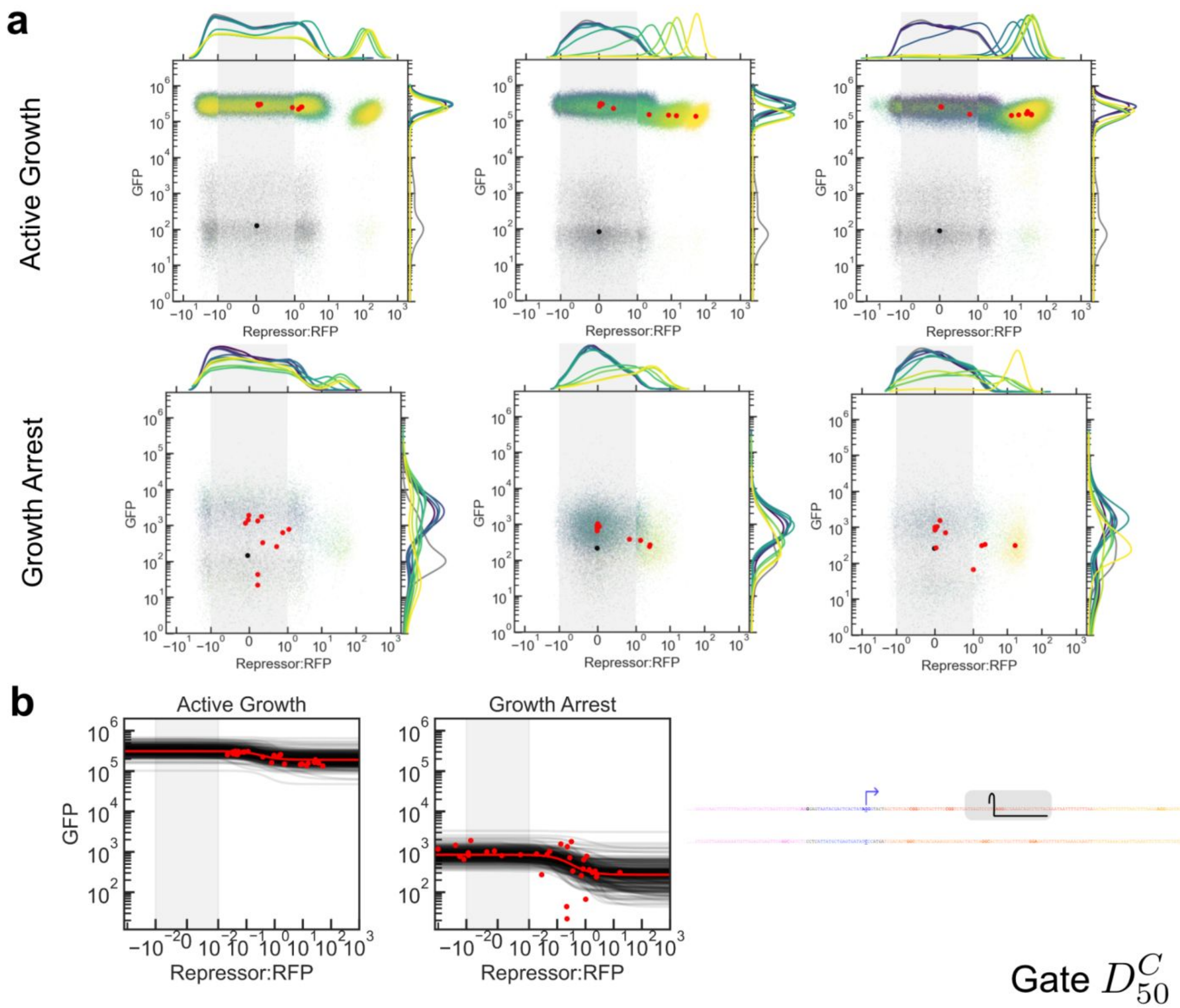

Supplemental Fig. 8: *Repression curves for gate $D^C{}_{50}$. (**a**) Flow cytometry data. Three biological replicates (columns) were measured on three different days. Dots represent medians of distributions for each induction condition, and colors represent atc concentrations. Induction conditions for atc were: Active Growth, Replicate 1: 0, 0.02, 0.06, 0.2, 0.43, 0.93, 2, 6, 20, 60, 200 ng/mL. Active Growth, Replicate 2: 0, 0.02, 0.2, 0.43, 0.93, 2, 3.5, 6.3, 11.3, 20, 200 ng/mL. Active Growth, Replicate 3: 0, 0.2, 0.43, 0.93, 2, 2.6, 3.5, 6.3, 11.3, 20, 200 ng/mL. Growth Arrest, all replicates: 0, 0.02, 0.06, 0.2, 0.43, 0.93, 2, 6, 20, 60, 200 ng/mL. Grey points indicate cultures that were not induced with either IPTG or atc. Data are plotted on a symmetric log scale where the grey shaded region is linear. (**b**) 300 trajectories taken from the end of the MCMC process overlaid against the median (RFP,GFP) values from all three replicates.*

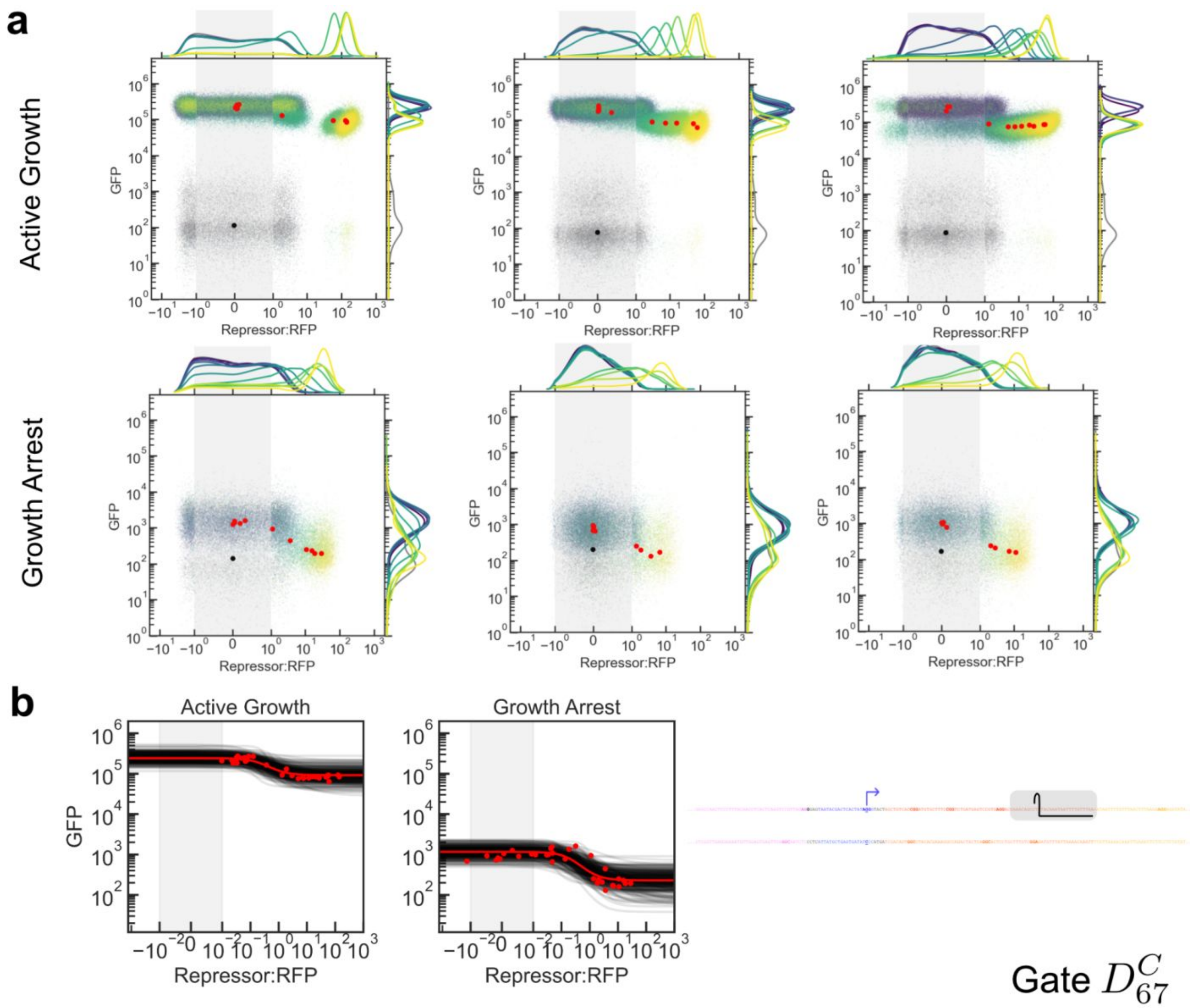

Supplemental Fig. 9: *Repression curves for gate D^{C}_{67}. (**a**) Flow cytometry data. Three biological replicates (columns) were measured on three different days. Dots represent medians of distributions for each induction condition, and colors represent atc concentrations. Induction conditions for atc were: Active Growth, Replicate 1: 0, 0.02, 0.06, 0.2, 0.43, 0.93, 2, 6, 20, 60, 200 ng/mL. Active Growth, Replicate 2: 0, 0.02, 0.2, 0.43, 0.93, 2, 3.5, 6.3, 11.3, 20, 200 ng/mL. Active Growth, Replicate 3: 0, 0.2, 0.43, 0.93, 2, 2.6, 3.5, 6.3, 11.3, 20, 200 ng/mL. Growth Arrest, all replicates: 0, 0.02, 0.06, 0.2, 0.43, 0.93, 2, 6, 20, 60, 200 ng/mL. Grey points indicate cultures that were not induced with either IPTG or atc. Data are plotted on a symmetric log scale where the grey shaded region is linear. (**b**) 300 trajectories taken from the end of the MCMC process overlaid against the median (RFP,GFP) values from all three replicates.*

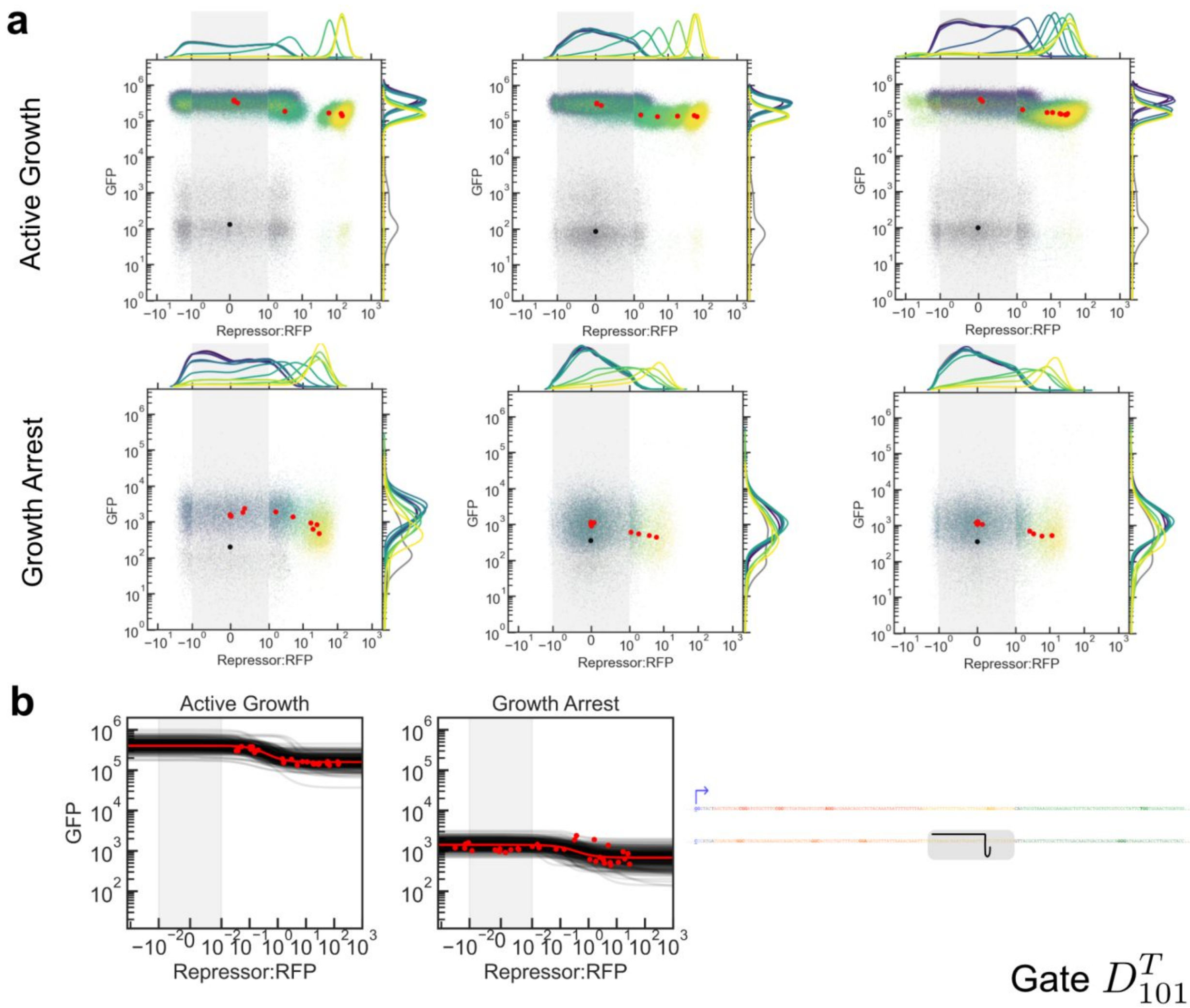

Supplemental Fig. 10: *Repression curves for gate D^{T}_{101}. (**a**) Flow cytometry data. Three biological replicates (columns) were measured on three different days. Dots represent medians of distributions for each induction condition, and colors represent atc concentrations. Induction conditions for atc were: Active Growth, Replicate 1: 0, 0.02, 0.06, 0.2, 0.43, 0.93, 2, 6, 20, 60, 200 ng/mL. Active Growth, Replicate 2: 0, 0.02, 0.2, 0.43, 0.93, 2, 3.5, 6.3, 11.3, 20, 200 ng/mL. Active Growth, Replicate 3: 0, 0.2, 0.43, 0.93, 2, 2.6, 3.5, 6.3, 11.3, 20, 200 ng/mL. Growth Arrest, all replicates: 0, 0.02, 0.06, 0.2, 0.43, 0.93, 2, 6, 20, 60, 200 ng/mL. Grey points indicate cultures that were not induced with either IPTG or atc. Data are plotted on a symmetric log scale where the grey shaded region is linear. (**b**) 300 trajectories taken from the end of the MCMC process overlaid against the median (RFP,GFP) values from all three replicates.*

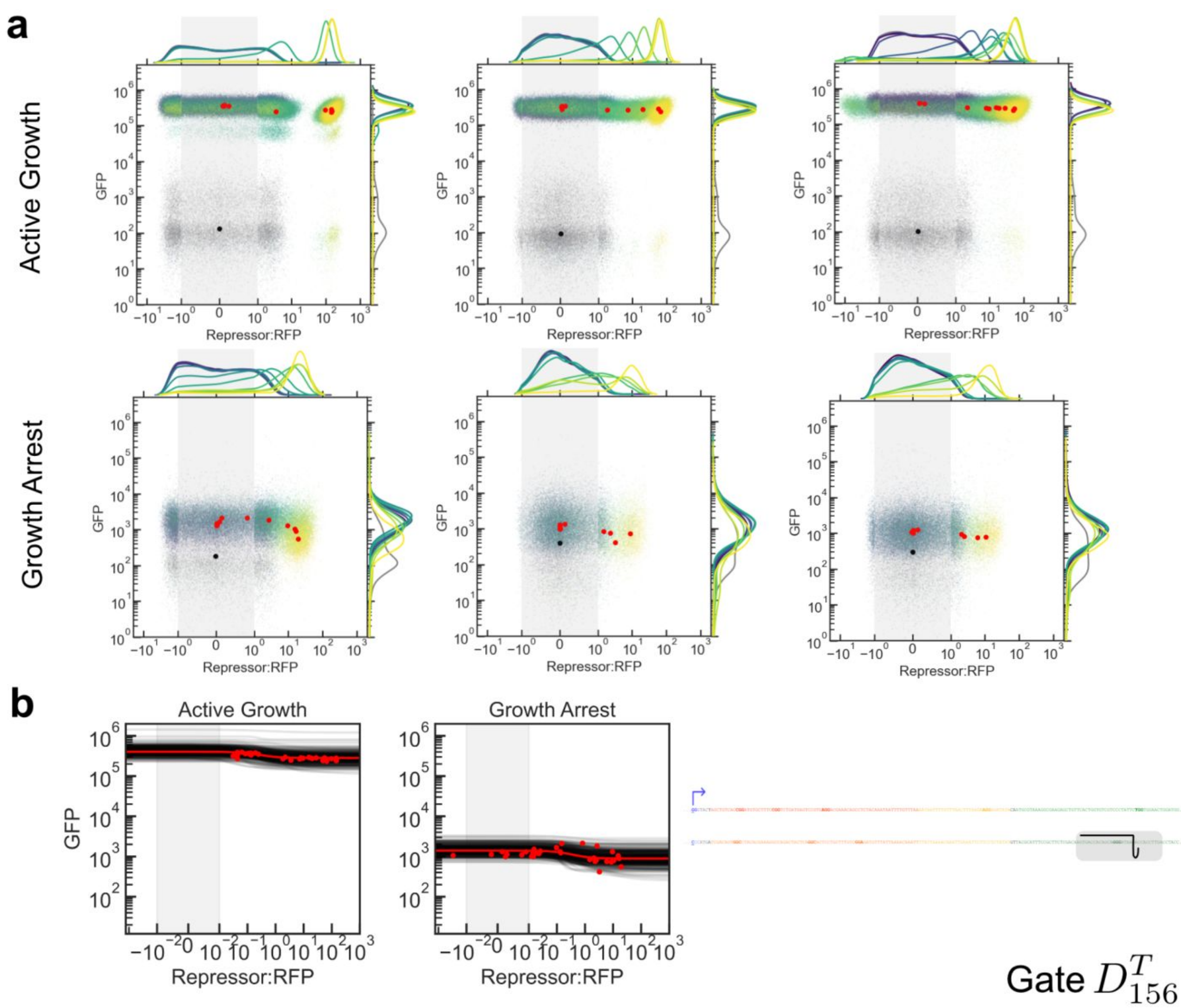

Supplemental Fig. 11: *Repression curves for gate D^T_{156}. (**a**) Flow cytometry data. Three biological replicates (columns) were measured on three different days. Dots represent medians of distributions for each induction condition, and colors represent atc concentrations. Induction conditions for atc were: Active Growth, Replicate 1: 0, 0.02, 0.06, 0.2, 0.43, 0.93, 2, 6, 20, 60, 200 ng/mL. Active Growth, Replicate 2: 0, 0.02, 0.2, 0.43, 0.93, 2, 3.5, 6.3, 11.3, 20, 200 ng/mL. Active Growth, Replicate 3: 0, 0.2, 0.43, 0.93, 2, 2.6, 3.5, 6.3, 11.3, 20, 200 ng/mL. Growth Arrest, all replicates: 0, 0.02, 0.06, 0.2, 0.43, 0.93, 2, 6, 20, 60, 200 ng/mL. Grey points indicate cultures that were not induced with either IPTG or atc. Data are plotted on a symmetric log scale where the grey shaded region is linear. (**b**) 300 trajectories taken from the end of the MCMC process overlaid against the median (RFP,GFP) values from all three replicates.*

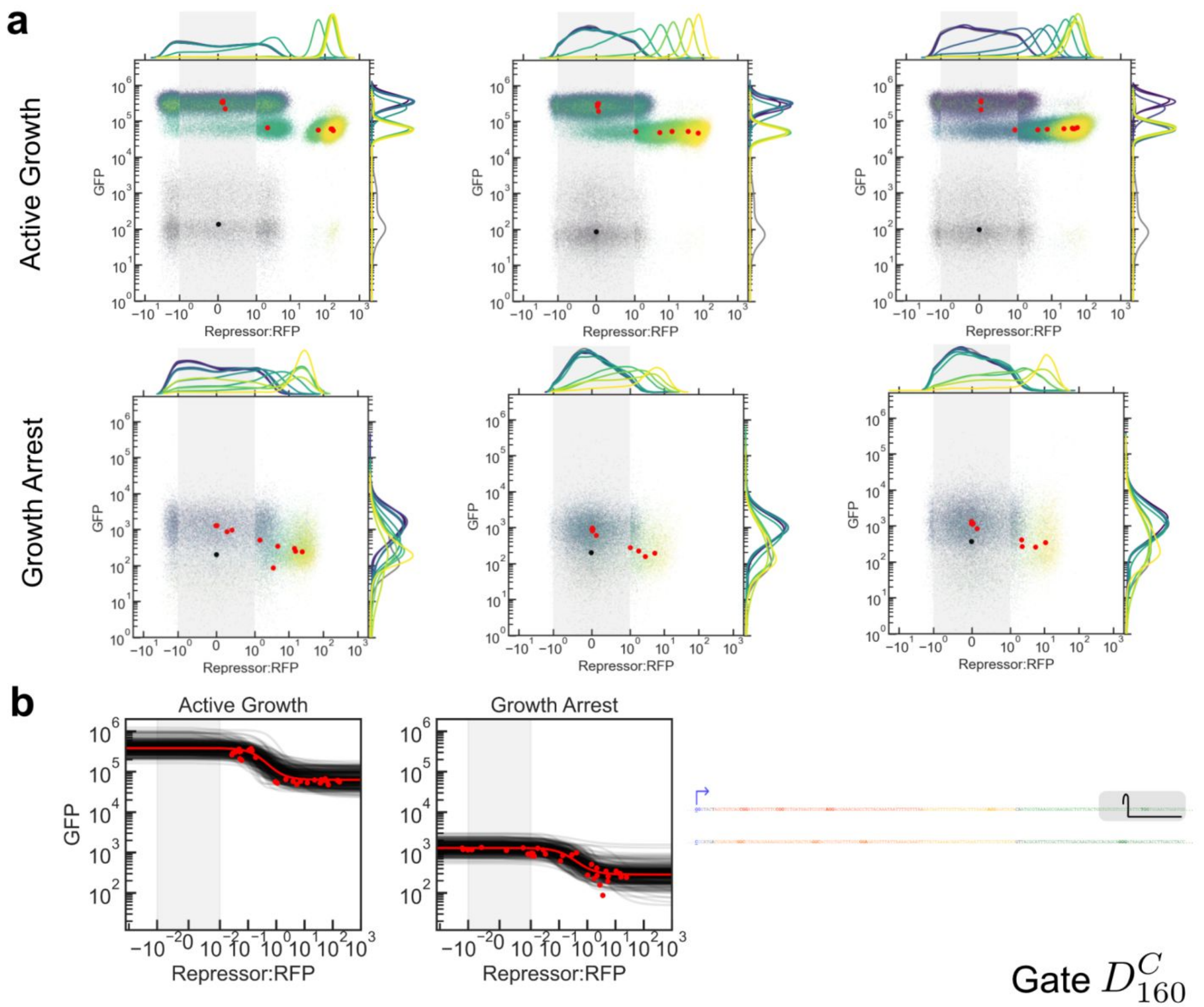

Supplemental Fig. 12: *Repression curves for gate D^{C}_{160}. (**a**) Flow cytometry data. Three biological replicates (columns) were measured on three different days. Dots represent medians of distributions for each induction condition, and colors represent atc concentrations. Induction conditions for atc were: Active Growth, Replicate 1: 0, 0.02, 0.06, 0.2, 0.43, 0.93, 2, 6, 20, 60, 200 ng/mL. Active Growth, Replicate 2: 0, 0.02, 0.2, 0.43, 0.93, 2, 3.5, 6.3, 11.3, 20, 200 ng/mL. Active Growth, Replicate 3: 0, 0.2, 0.43, 0.93, 2, 2.6, 3.5, 6.3, 11.3, 20, 200 ng/mL. Growth Arrest, all replicates: 0, 0.02, 0.06, 0.2, 0.43, 0.93, 2, 6, 20, 60, 200 ng/mL. Grey points indicate cultures that were not induced with either IPTG or atc. Data are plotted on a symmetric log scale where the grey shaded region is linear. (**b**) 300 trajectories taken from the end of the MCMC process overlaid against the median (RFP,GFP) values from all three replicates.*

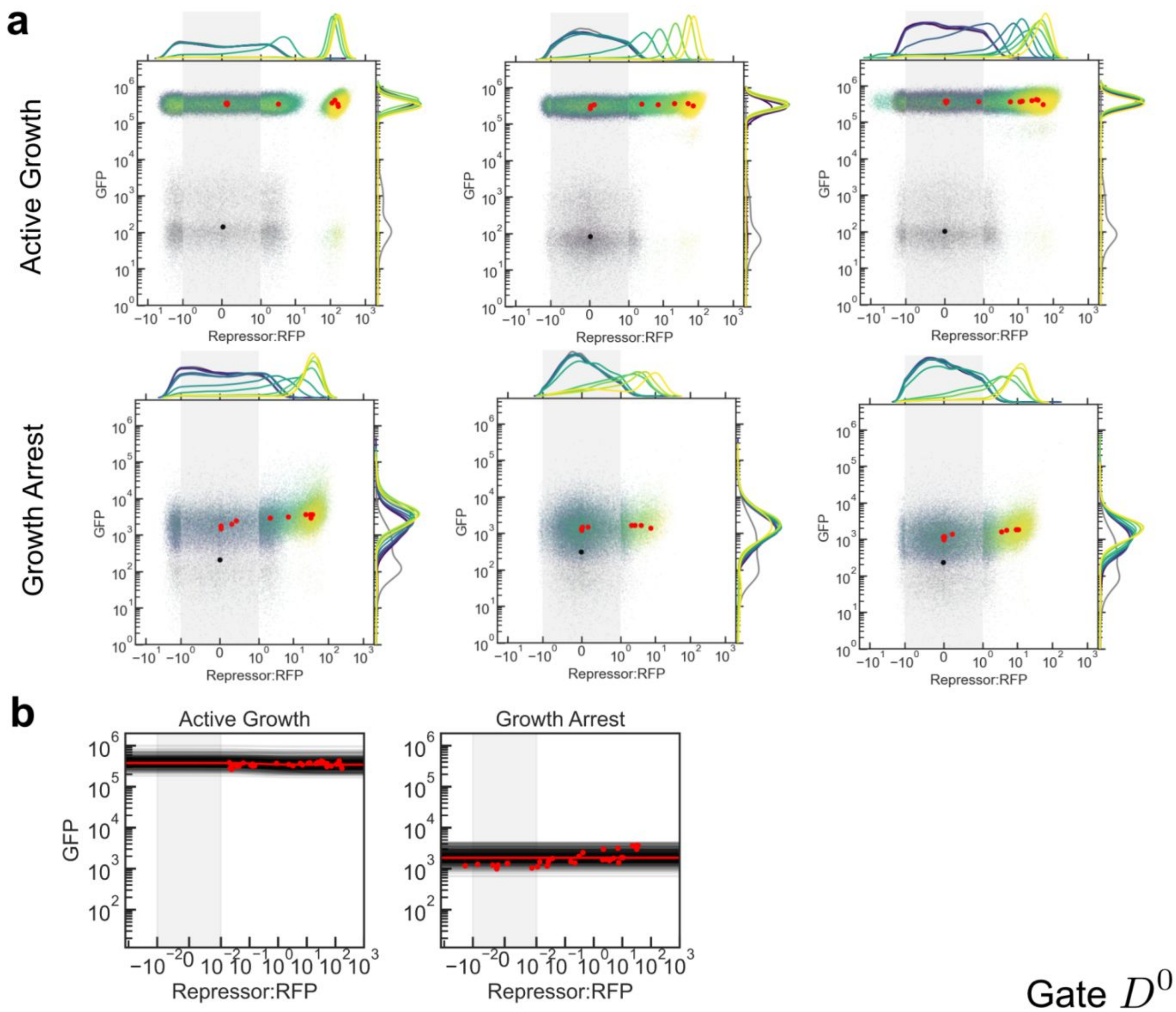

Supplemental Fig. 13: *Repression curves for the negative control gate D^0. (**a**) Flow cytometry data. Three biological replicates (columns) were measured on three different days. Dots represent medians of distributions for each induction condition, and colors represent atc concentrations. Induction conditions for atc were: Active Growth, Replicate 1: 0, 0.02, 0.06, 0.2, 0.43, 0.93, 2, 6, 20, 60, 200 ng/mL. Active Growth, Replicate 2: 0, 0.02, 0.2, 0.43, 0.93, 2, 3.5, 6.3, 11.3, 20, 200 ng/mL. Active Growth, Replicate 3: 0, 0.2, 0.43, 0.93, 2, 2.6, 3.5, 6.3, 11.3, 20, 200 ng/mL. Growth Arrest, all replicates: 0, 0.02, 0.06, 0.2, 0.43, 0.93, 2, 6, 20, 60, 200 ng/mL. Grey points indicate cultures that were not induced with either IPTG or atc. Data are plotted on a symmetric log scale where the grey shaded region is linear. (**b**) 300 trajectories taken from the end of the MCMC process overlaid against the median (RFP,GFP) values from all three replicates.*

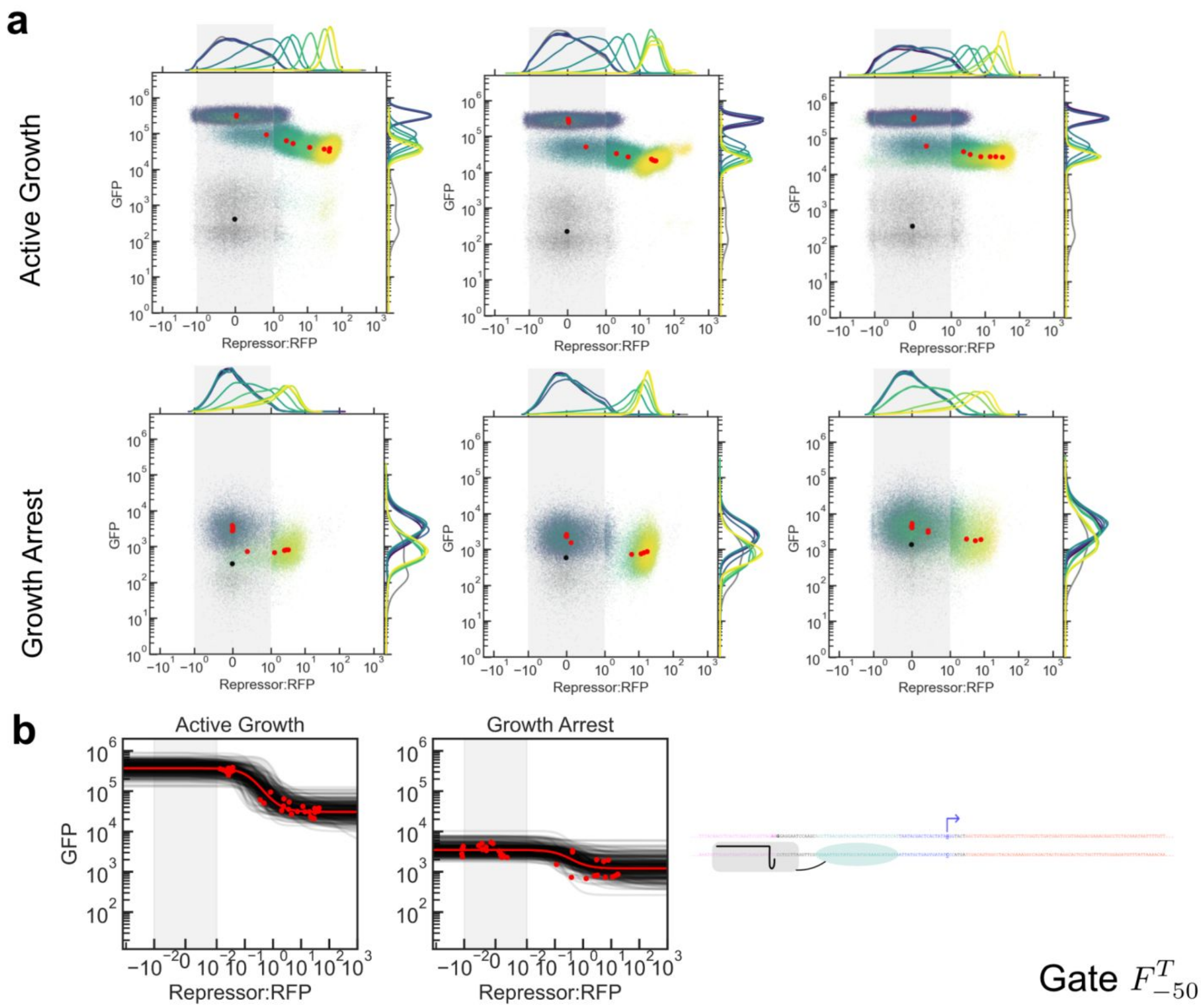

Supplemental Fig. 14: *Repression curves for gate F^{T}_{-50}. (**a**) Flow cytometry data. Three biological replicates (columns) were measured on three different days. Dots represent medians of distributions for each induction condition, and colors represent atc concentrations. Induction conditions for atc were: Active Growth, all replicates: 0, 0.2, 0.43, 0.93, 2, 2.6, 3.5, 6.3, 11.3, 20, 100 ng/mL. Growth Arrest, all replicates: 0, 0.02, 0.06, 0.2, 0.43, 0.93, 2, 6, 20, 60, 200 ng/mL. Grey points indicate cultures that were not induced with either IPTG or atc. Data are plotted on a symmetric log scale where the grey shaded region is linear. (**b**) 300 trajectories taken from the end of the MCMC process overlaid against the median (RFP,GFP) values from all three replicates.*

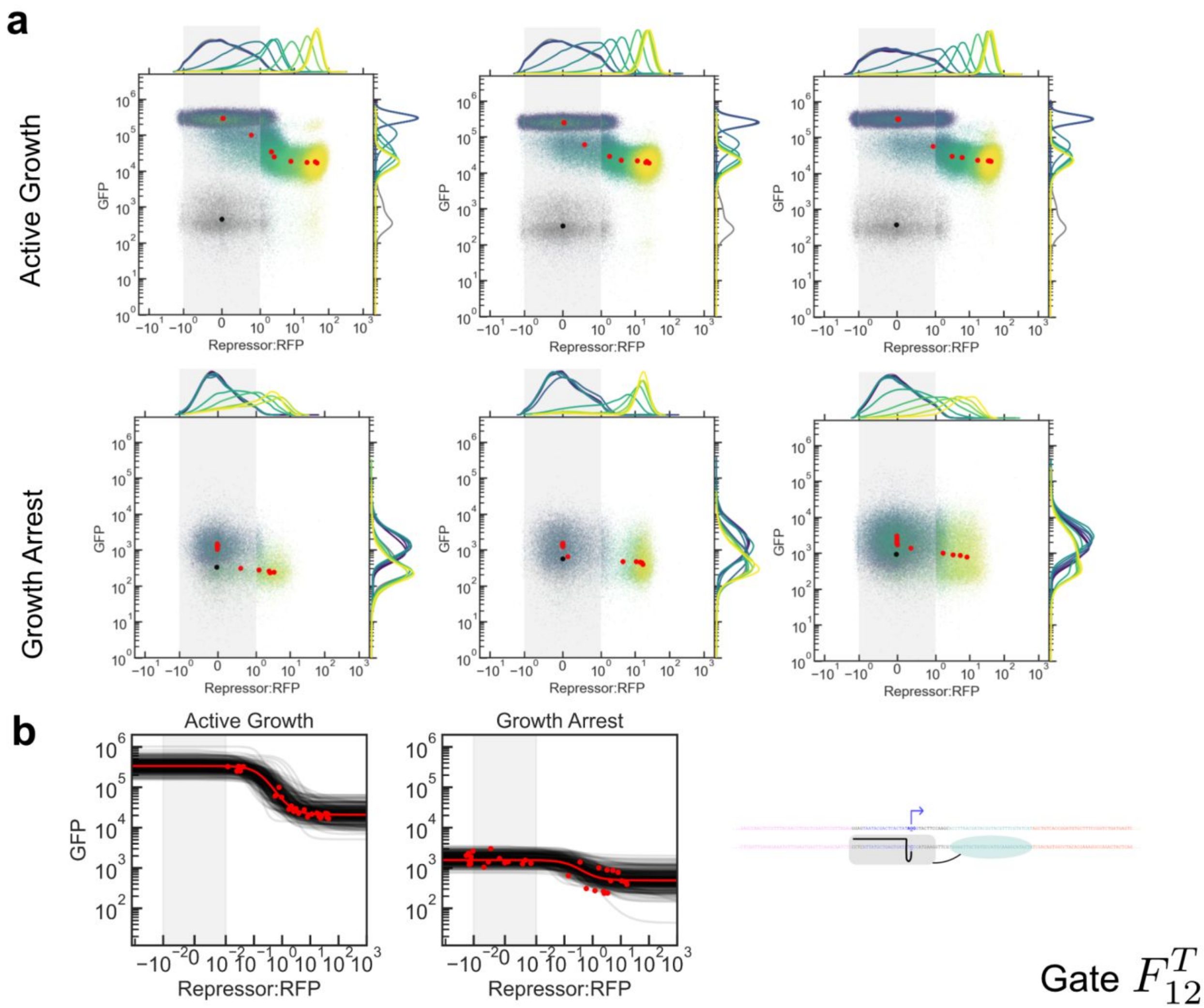

Supplemental Fig. 15: *Repression curves for gate F^{T}_{12}. (**a**) Flow cytometry data. Three biological replicates (columns) were measured on three different days. Dots represent medians of distributions for each induction condition, and colors represent atc concentrations. Induction conditions for atc were: Active Growth, all replicates: 0, 0.2, 0.43, 0.93, 2, 2.6, 3.5, 6.3, 11.3, 20, 100 ng/mL. Growth Arrest, all replicates: 0, 0.02, 0.06, 0.2, 0.43, 0.93, 2, 6, 20, 60, 200 ng/mL. Grey points indicate cultures that were not induced with either IPTG or atc. Data are plotted on a symmetric log scale where the grey shaded region is linear. (**b**) 300 trajectories taken from the end of the MCMC process overlaid against the median (RFP,GFP) values from all three replicates.*

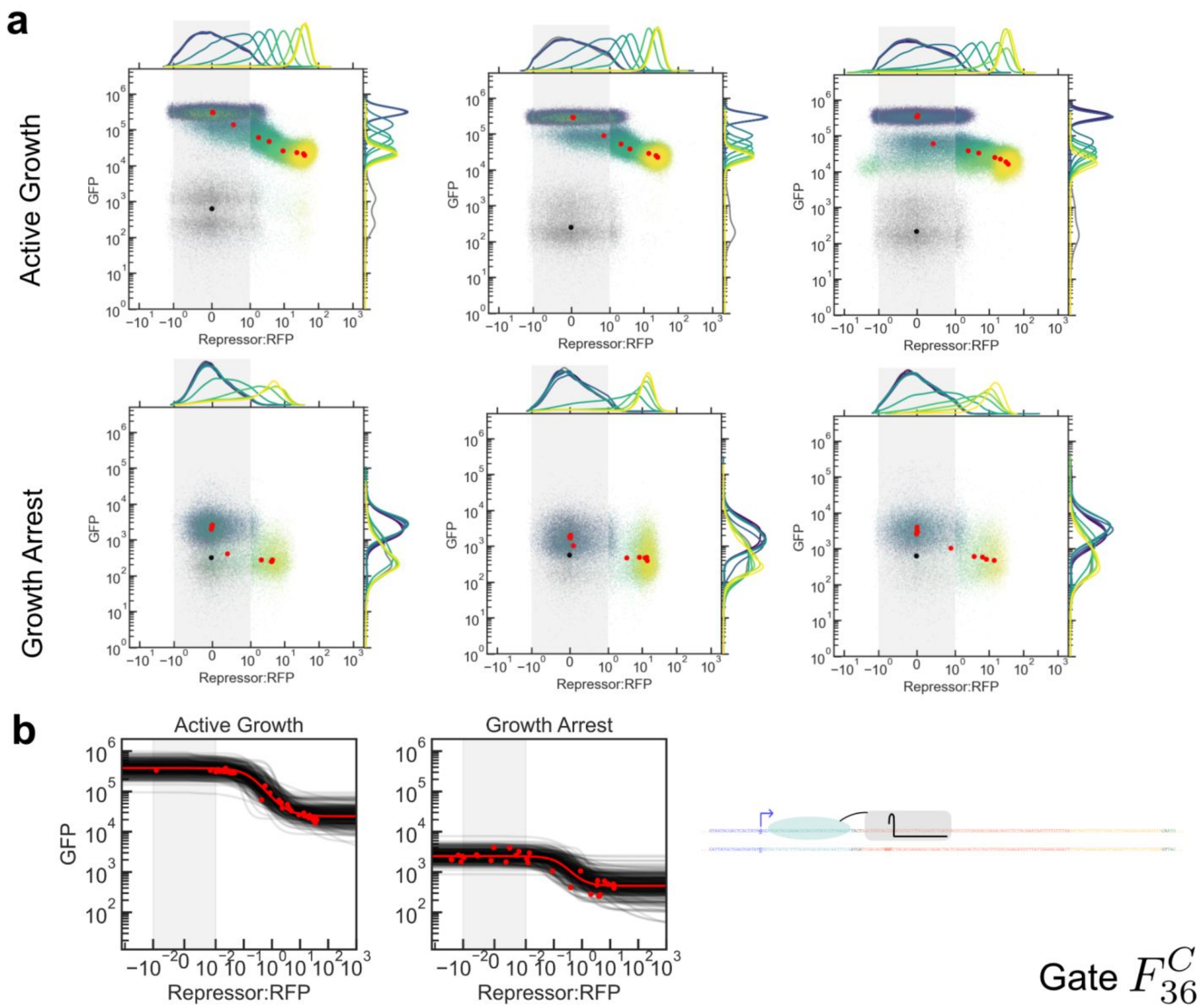

Supplemental Fig. 16: *Repression curves for gate F^{C}_{36}. (**a**) Flow cytometry data. Three biological replicates (columns) were measured on three different days. Dots represent medians of distributions for each induction condition, and colors represent atc concentrations. Induction conditions for atc were: Active Growth, all replicates: 0, 0.2, 0.43, 0.93, 2, 2.6, 3.5, 6.3, 11.3, 20, 100 ng/mL. Growth Arrest, all replicates: 0, 0.02, 0.06, 0.2, 0.43, 0.93, 2, 6, 20, 60, 200 ng/mL. Grey points indicate cultures that were not induced with either IPTG or atc. Data are plotted on a symmetric log scale where the grey shaded region is linear. (**b**) 300 trajectories taken from the end of the MCMC process overlaid against the median (RFP,GFP) values from all three replicates.*

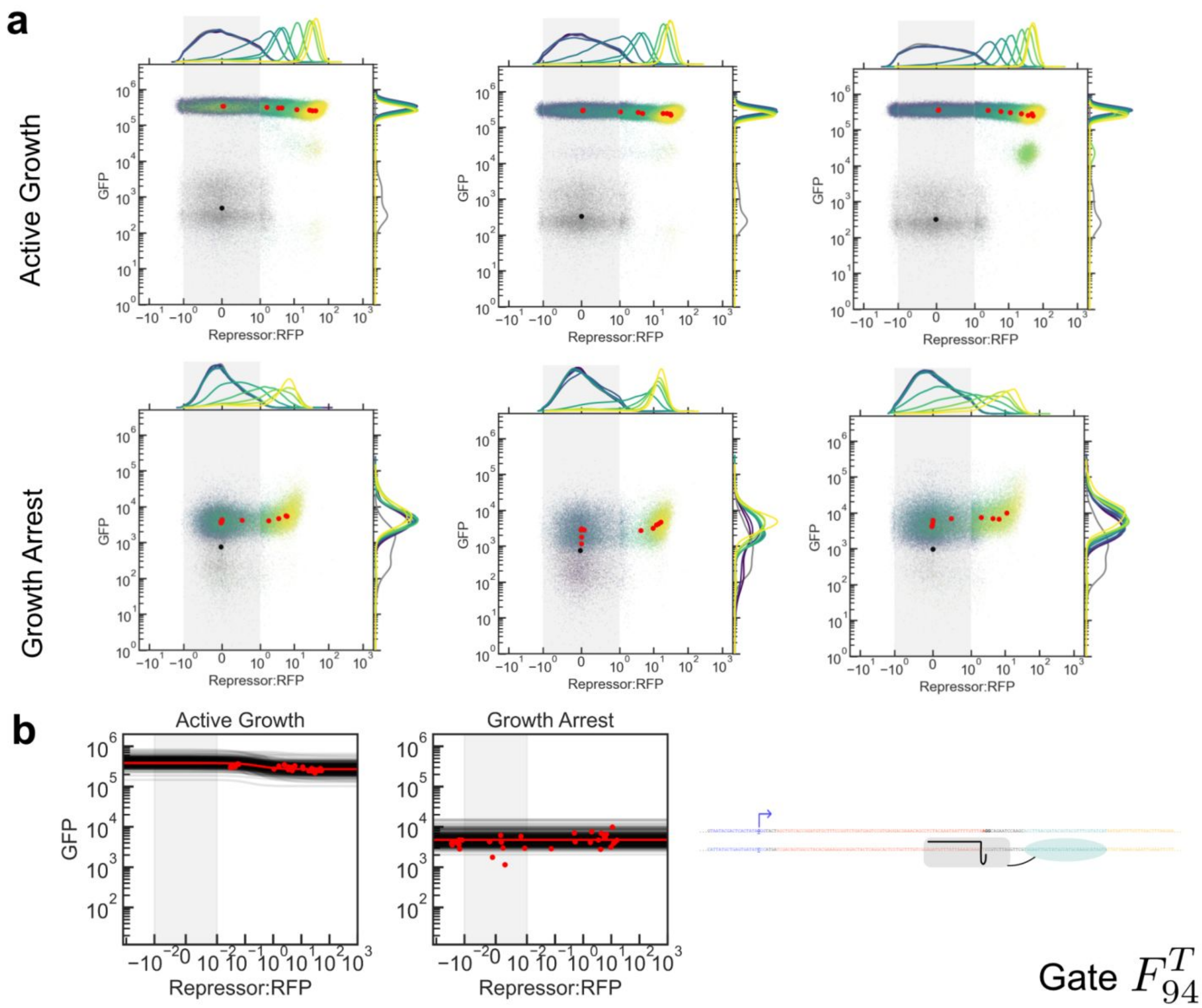

Supplemental Fig. 17: *Repression curves for gate F^{T}_{94}. (**a**) Flow cytometry data. Three biological replicates (columns) were measured on three different days. Dots represent medians of distributions for each induction condition, and colors represent atc concentrations. Induction conditions for atc were: Active Growth, all replicates: 0, 0.2, 0.43, 0.93, 2, 2.6, 3.5, 6.3, 11.3, 20, 100 ng/mL. Growth Arrest, all replicates: 0, 0.02, 0.06, 0.2, 0.43, 0.93, 2, 6, 20, 60, 200 ng/mL. Grey points indicate cultures that were not induced with either IPTG or atc. Data are plotted on a symmetric log scale where the grey shaded region is linear. (**b**) 300 trajectories taken from the end of the MCMC process overlaid against the median (RFP,GFP) values from all three replicates.*

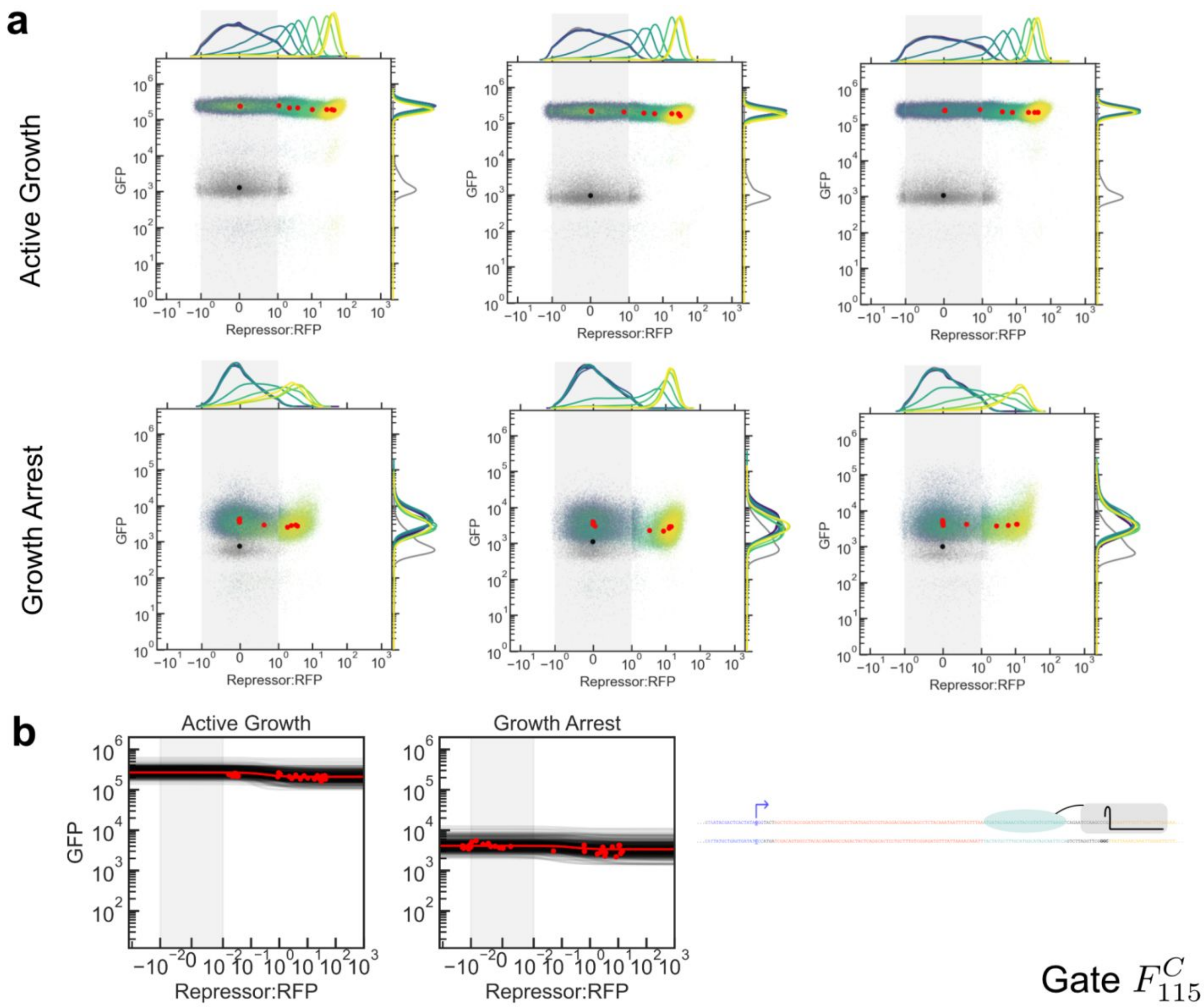

Supplemental Fig. 18: *Repression curves for gate F^{C}_{115}. (**a**) Flow cytometry data. Three biological replicates (columns) were measured on three different days. Dots represent medians of distributions for each induction condition, and colors represent atc concentrations. Induction conditions for atc were: Active Growth, all replicates: 0, 0.2, 0.43, 0.93, 2, 2.6, 3.5, 6.3, 11.3, 20, 100 ng/mL. Growth Arrest, all replicates: 0, 0.02, 0.06, 0.2, 0.43, 0.93, 2, 6, 20, 60, 200 ng/mL. Grey points indicate cultures that were not induced with either IPTG or atc. Data are plotted on a symmetric log scale where the grey shaded region is linear. (**b**) 300 trajectories taken from the end of the MCMC process overlaid against the median (RFP,GFP) values from all three replicates.*

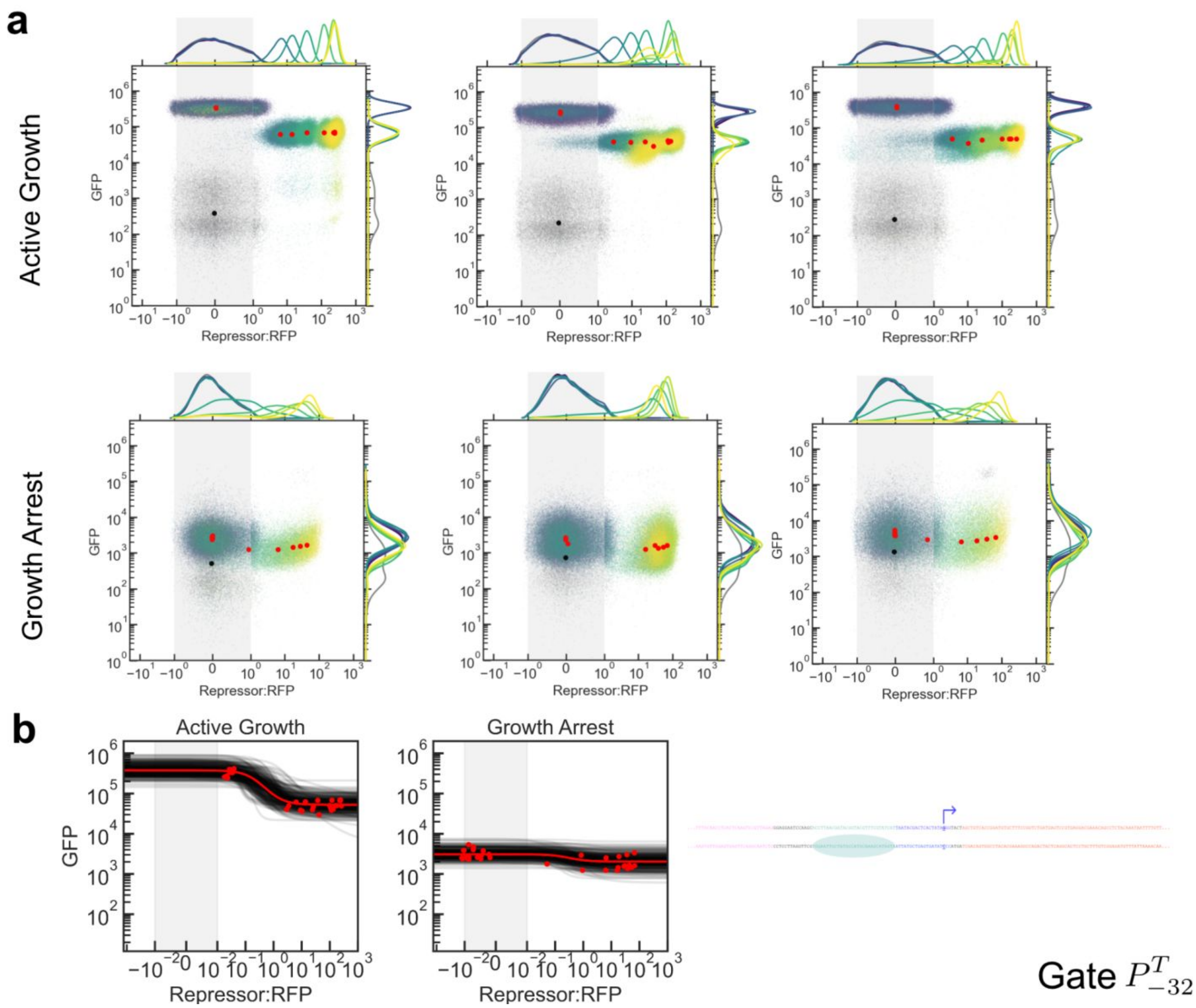

Supplemental Fig. 19: *Repression curves for gate P^T_{-32}. (**a**) Flow cytometry data. Three biological replicates (columns) were measured on three different days. Dots represent medians of distributions for each induction condition, and colors represent atc concentrations. Induction conditions for atc were: Active Growth, all replicates: 0, 0.2, 0.43, 0.93, 2, 2.6, 3.5, 6.3, 11.3, 20, 100 ng/mL. Growth Arrest, all replicates: 0, 0.02, 0.06, 0.2, 0.43, 0.93, 2, 6, 20, 60, 200 ng/mL. Grey points indicate cultures that were not induced with either IPTG or atc. Data are plotted on a symmetric log scale where the grey shaded region is linear. (**b**) 300 trajectories taken from the end of the MCMC process overlaid against the median (RFP,GFP) values from all three replicates.*

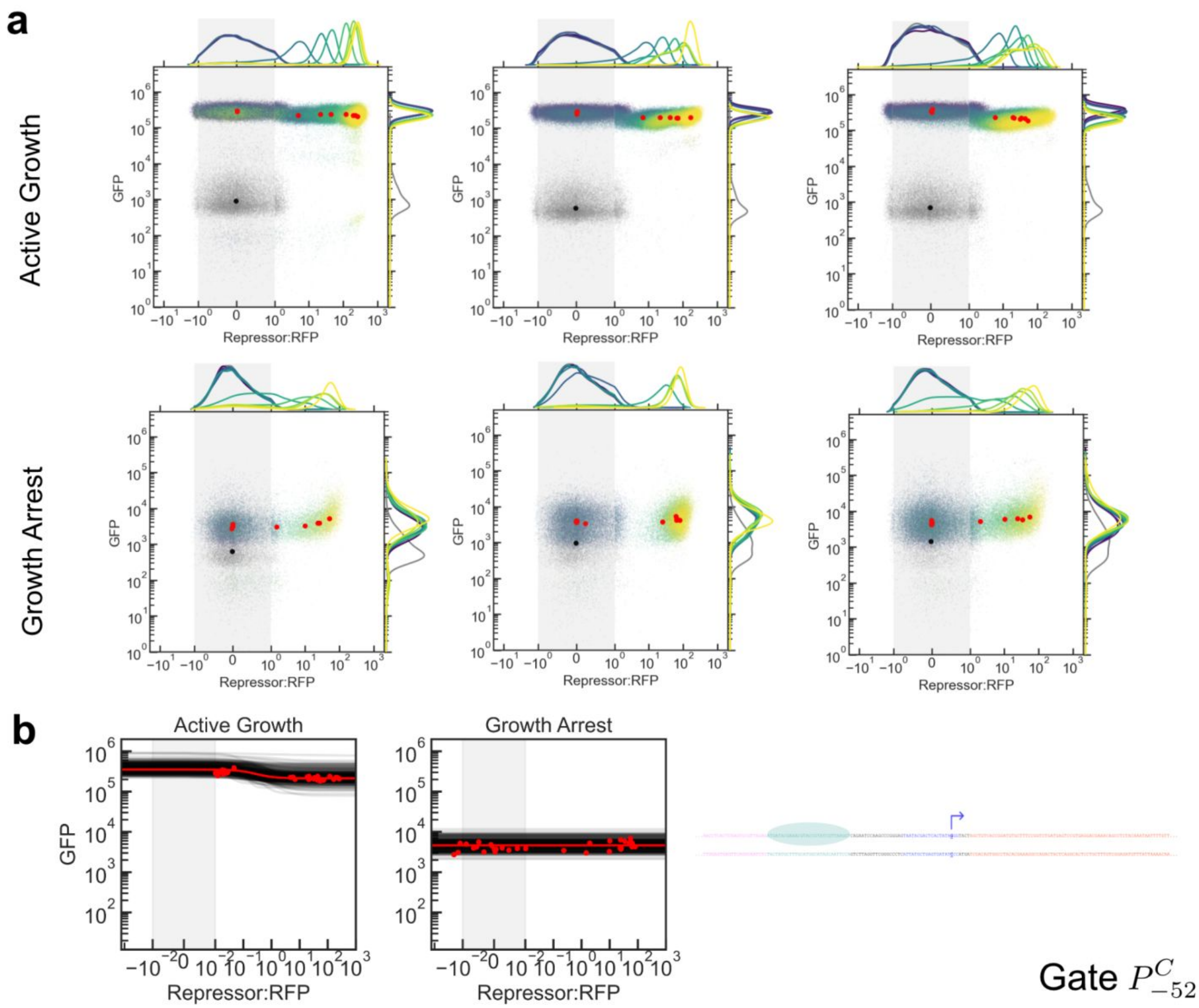

Supplemental Fig. 20: *Repression curves for gate P^{C}_{-52}. (**a**) Flow cytometry data. Three biological replicates (columns) were measured on three different days. Dots represent medians of distributions for each induction condition, and colors represent atc concentrations. Induction conditions for atc were: Active Growth, all replicates: 0, 0.2, 0.43, 0.93, 2, 2.6, 3.5, 6.3, 11.3, 20, 100 ng/mL. Growth Arrest, all replicates: 0, 0.02, 0.06, 0.2, 0.43, 0.93, 2, 6, 20, 60, 200 ng/mL. Grey points indicate cultures that were not induced with either IPTG or atc. Data are plotted on a symmetric log scale where the grey shaded region is linear. (**b**) 300 trajectories taken from the end of the MCMC process overlaid against the median (RFP,GFP) values from all three replicates.*

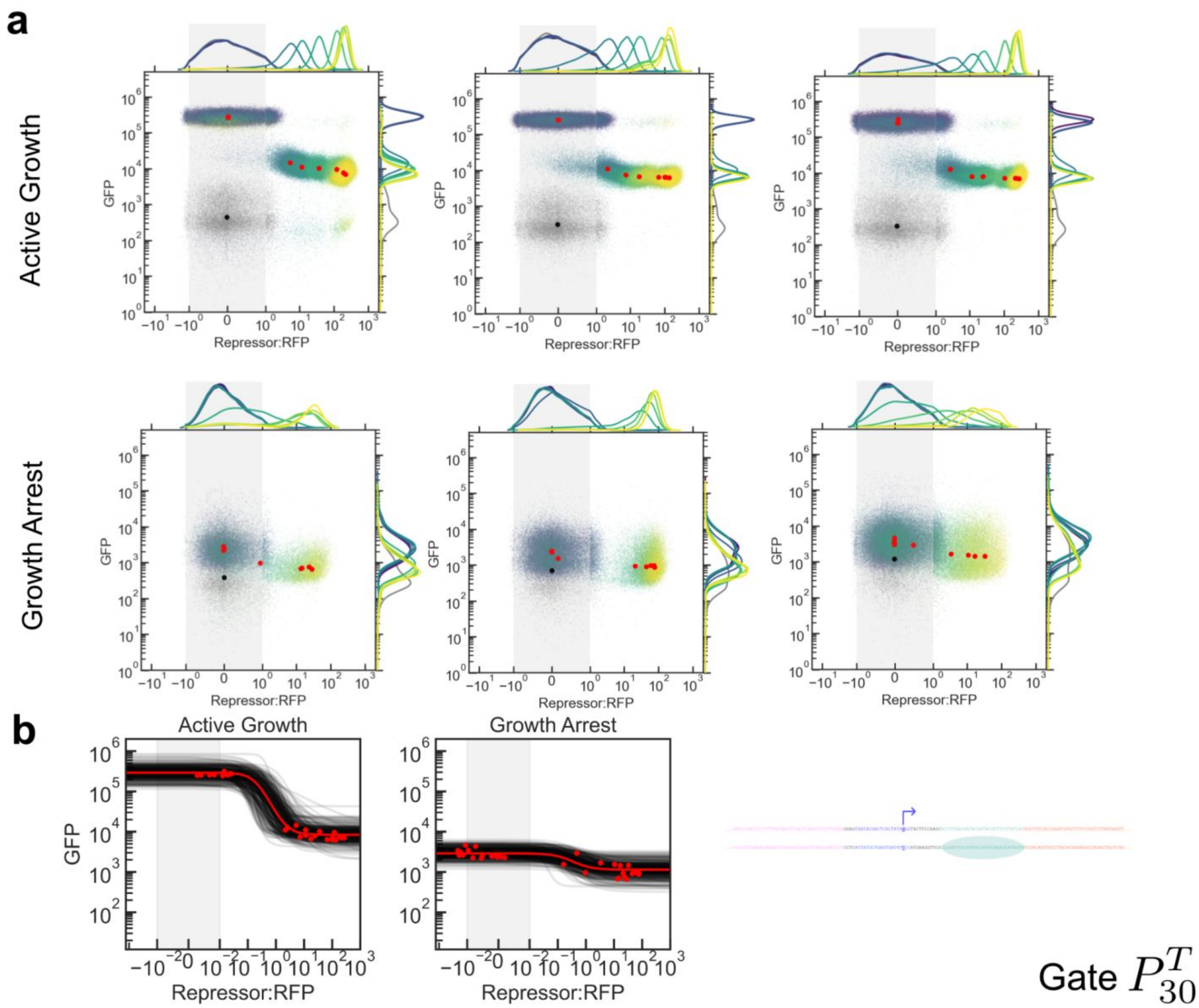

Supplemental Fig. 21: *Repression curves for gate P^{T}_{30}. (**a**) Flow cytometry data. Three biological replicates (columns) were measured on three different days. Dots represent medians of distributions for each induction condition, and colors represent atc concentrations. Induction conditions for atc were: Active Growth, all replicates: 0, 0.2, 0.43, 0.93, 2, 2.6, 3.5, 6.3, 11.3, 20, 100 ng/mL. Growth Arrest, all replicates: 0, 0.02, 0.06, 0.2, 0.43, 0.93, 2, 6, 20, 60, 200 ng/mL. Grey points indicate cultures that were not induced with either IPTG or atc. Data are plotted on a symmetric log scale where the grey shaded region is linear. (**b**) 300 trajectories taken from the end of the MCMC process overlaid against the median (RFP,GFP) values from all three replicates.*

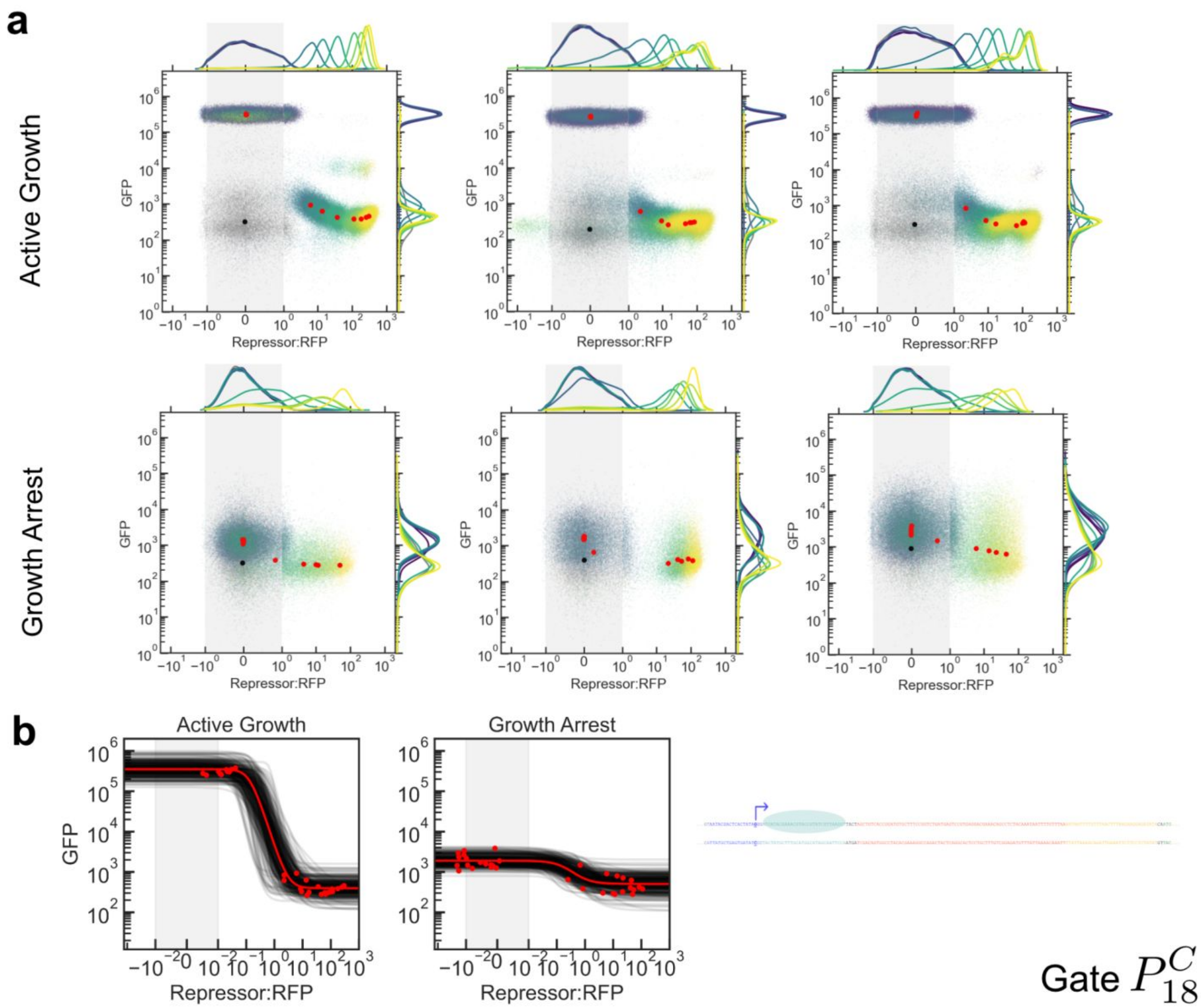

Supplemental Fig. 22: *Repression curves for gate $P^C{}_{18}$. (**a**) Flow cytometry data. Three biological replicates (columns) were measured on three different days. Dots represent medians of distributions for each induction condition, and colors represent atc concentrations. Induction conditions for atc were: Active Growth, all replicates: 0, 0.2, 0.43, 0.93, 2, 2.6, 3.5, 6.3, 11.3, 20, 100 ng/mL. Growth Arrest, all replicates: 0, 0.02, 0.06, 0.2, 0.43, 0.93, 2, 6, 20, 60, 200 ng/mL. Grey points indicate cultures that were not induced with either IPTG or atc. Data are plotted on a symmetric log scale where the grey shaded region is linear. (**b**) 300 trajectories taken from the end of the MCMC process overlaid against the median (RFP,GFP) values from all three replicates.*

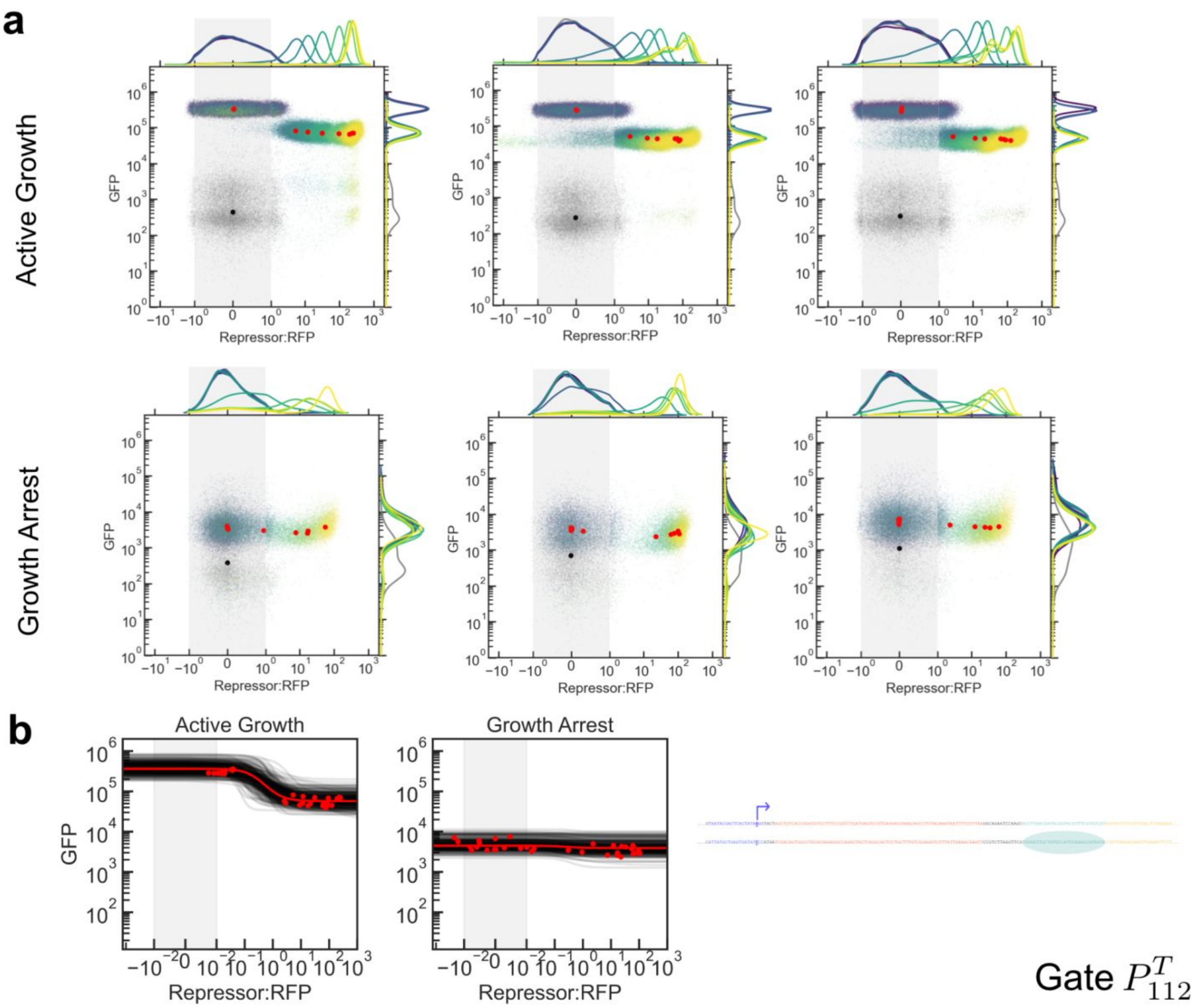

Supplemental Fig. 23: *Repression curves for gate P^{T}_{112}. (**a**) Flow cytometry data. Three biological replicates (columns) were measured on three different days. Dots represent medians of distributions for each induction condition, and colors represent atc concentrations. Induction conditions for atc were: Active Growth, all replicates: 0, 0.2, 0.43, 0.93, 2, 2.6, 3.5, 6.3, 11.3, 20, 100 ng/mL. Growth Arrest, all replicates: 0, 0.02, 0.06, 0.2, 0.43, 0.93, 2, 6, 20, 60, 200 ng/mL. Grey points indicate cultures that were not induced with either IPTG or atc. Data are plotted on a symmetric log scale where the grey shaded region is linear. (**b**) 300 trajectories taken from the end of the MCMC process overlaid against the median (RFP,GFP) values from all three replicates.*

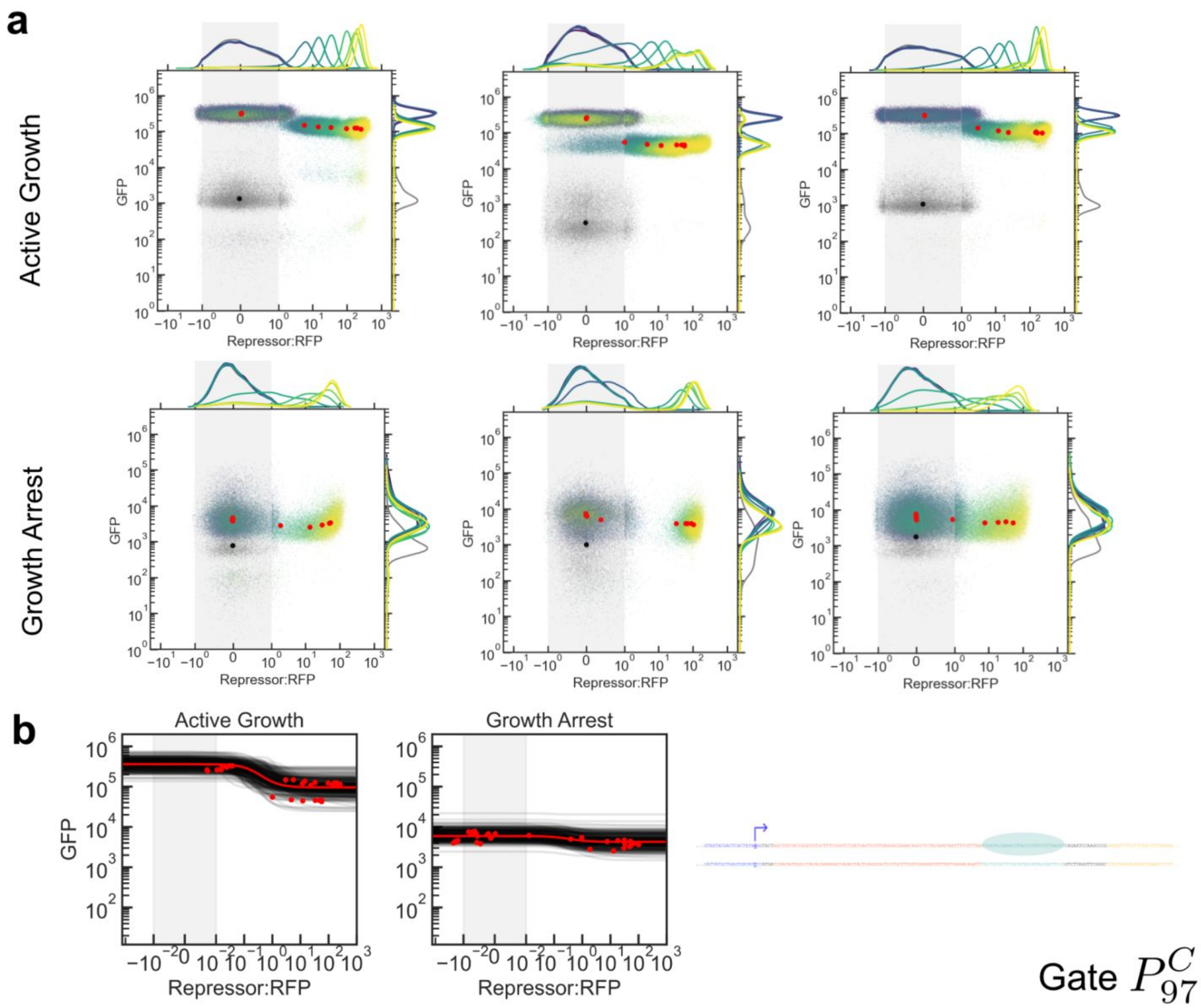

Supplemental Fig. 24: *Repression curves for gate P^{C}_{97}. (**a**) Flow cytometry data. Three biological replicates (columns) were measured on three different days. Dots represent medians of distributions for each induction condition, and colors represent atc concentrations. Induction conditions for atc were: Active Growth, all replicates: 0, 0.2, 0.43, 0.93, 2, 2.6, 3.5, 6.3, 11.3, 20, 100 ng/mL. Growth Arrest, all replicates: 0, 0.02, 0.06, 0.2, 0.43, 0.93, 2, 6, 20, 60, 200 ng/mL. Grey points indicate cultures that were not induced with either IPTG or atc. Data are plotted on a symmetric log scale where the grey shaded region is linear. (**b**) 300 trajectories taken from the end of the MCMC process overlaid against the median (RFP,GFP) values from all three replicates.*

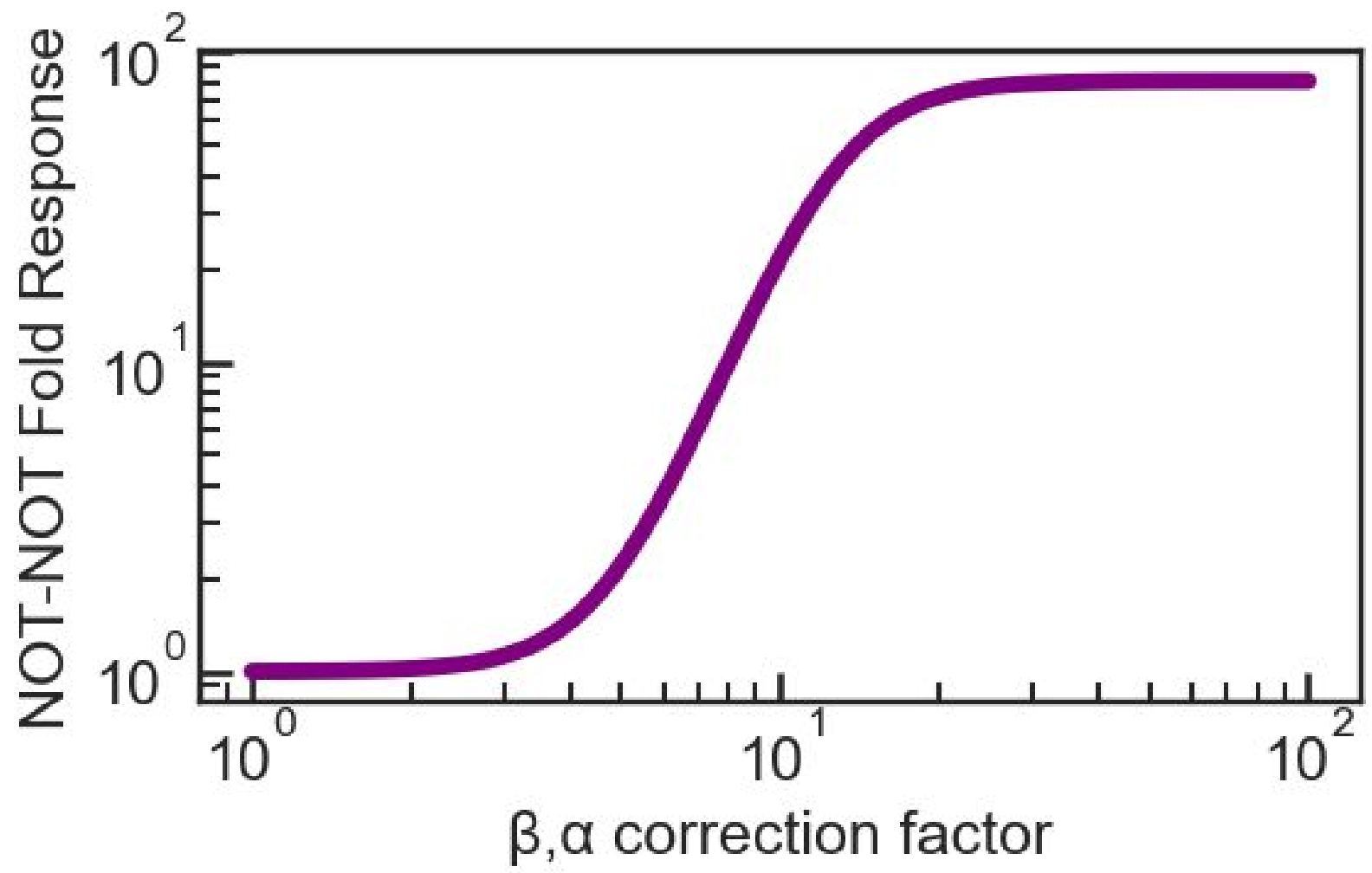

Supplemental Fig. 25: *Predicted impact of mitigating the growth arrest-associated reduction in β, α on circuit peformance under growth arrest for the NOT-NOT circuit in Fig. 6. Fold response is calculated as the maximal possible OFF to ON fold change achievable by the NOT-NOT circuit, which is $H_2(\alpha_1)/H_2(\beta_1+\alpha_1)$ where $H_2(x)$ is the Hill function of the second repression curve (PhlF) and β_1, α_1 are parameters from the first repression curve (SrpR). The β, α correction factor is globally multiplied against the growth-arrested values of β and α in Fig. 6, so a correction factor of 1 (i.e., no correction) leads to the circuit performance shown in Fig. 6.*

Supplemental Data

Cassette	Sequence
T7, GFP cassette (Expression cassette for all D gates)<br><br>UNS 1 - PLacO - B0033 - T7 RNAP - T12m - UNS 5 - PT7 - RiboJ - UTR1 - sfGFP - T7 Terminator - UNS X	CATTACTCGCATCCATTCTCAGGCTGTCTCGTCTCGTCTCGGAGAATTGTGAGCGGATAACAATTGACATTGTGAGCGGA<br>TAACAAGATACTGAGCACATCAGCAGGACGCACTGACCTACTAGAGTCACACAGGACTACTAAATGAACACGATTAACAT<br>CGCTAAGAACGACTTCTCTGACATCGAACTGGCTGCTATCCCGTTCAACACTCTGGCTGACCATTACGGTGAGCGTTTAG<br>CTCGCGAACAGTTGGCCCTTGAGCATGAGTCTTACGAGATGGGTGAAGCACGCTTCCGCAAGATGTTTGAGCGTCAACTT<br>AAAGCTGGTGAGGTTGCGGATAACGCTGCCGCCAAGCCTCTCATCACTACCCTACTCCCTAAGATGATTGCACGCATCAA<br>CGACTGGTTTGAGGAAGTGAAAGCTAAGCGCGGCAAGCGCCCGACAGCCTTCCAGTTCCTGCAAGAAATCAAGCCGGAAG<br>CCGTAGCGTACATCACCATTAAGACCACTCTGGCTTGCCTAACCAGTGCTGACAATACAACCGTTCAGGCTGTAGCAAGC<br>GCAATCGGTCGGGCCATTGAGGACGAGGCTCGCTTCGGTCGTATCCGTGACCTTGAAGCTAAGCACTTCAAGAAAAACGT<br>TGAGGAACAACTCAACAAGCGCGTAGGGCACGTCTACAAGAAAGCATTTATGCAAGTTGTCGAGGCTGACATGCTCTCTA<br>AGGGTCTACTCGGTGGCGAGGCGTGGTCTTCGTGGCATAAGGAAGACTCTATTCATGTAGGAGTACGCTGCATCGAGATG<br>CTCATTGAGTCAACCGGAATGGTTAGCTTACACCGCCAAAATGCTGGCGTAGTAGGTCAAGACTCTGAGACTATCGAACT<br>CGCACCTGAATACGCTGAGGCTATCGCAACCCGTGCAGGTGCGCTGGCTGGCATCTCTCCGATGTTCCAACCTTGCGTAG<br>TTCCTCCTAAGCCGTGGACTGGCATTACTGGTGGTGGCTATTGGGCTAACGGTCGTCGTCCTCTGGCGCTGGTGCGTACT<br>CACAGTAAGAAAGCACTGATGCGCTACGAAGACGTTTACATGCCTGAGGTGTACAAAGCGATTAACATTGCGCAAAACAC<br>CGCATGGAAAATCAACAAGAAAGTCCTAGCGGTCGCCAACGTAATCACCAAGTGGAAGCATTGTCCGGTCGAGGACATCC<br>CTGCGATTGAGCGTGAAGAACTCCCGATGAAACCGGAAGACATCGACATGAATCCTGAGGCTCTCACCGCGTGGAAACGT<br>GCTGCCGCTGCTGTGTACCGCAAGGACAAGGCTCGCAAGTCTCGCCGTATCAGCCTTGAGTTCATGCTTGAGCAAGCCAA<br>TAAGTTTGCTAACCATAAGGCCATCTGGTTCCCTTACAACATGGACTGGCGCGGTCGTGTTTACGCTGTGTCAATGTTCA<br>ACCCGCAAGGTAACGATATGACCAAAGGACTGCTTACGCTGGCGAAAGGTAAACCAATCGGTAAGGAAGGTTACTACTGG<br>CTGAAAATCCACGGTGCAAACTGTGCGGGTGTCGATAAGGTTCCGTTCCCTGAGCGCATCAAGTTCATTGAGGAAAACCA<br>CGAGAACATCATGGCTTGCGCTAAGTCTCCACTGGAGAACACTTGGTGGGCTGAGCAAGATTCTCCGTTCTGCTTCCTTG<br>CGTTCTGCTTTGAGTACGCTGGGGTACAGCACCACGGCCTGAGCTATAACTGCTCCCTTCCGCTGGCGTTTGACGGGTCT<br>TGCTCTGGCATCCAGCACTTCTCCGCGATGCTCCGAGATGAGGTAGGTGGTCGCGCGGTTAACTTGCTTCCTAGTGAAAC<br>CGTTCAGGACATCTACGGGATTGTTGCTAAGAAAGTCAACGAGATTCTACAAGCAGACGCAATCAATGGGACCGATAACG<br>AAGTAGTTACCGTGACCGATGAGAACACTGGTGAAATCTCTGAGAAAGTCAAGCTGGGCACTAAGGCACTGGCTGGTCAA<br>TGGCTGGCTTACGGTGTTACTCGCAGTGTGACTAAGCGTTCAGTCATGACGCTGGCTTACGGGTCCAAAGAGTTCGGCTT<br>CCGTCAACAAGTGCTGGAAGATACCATTCAGCCAGCTATTGATTCCGGCAAGGGTCTGATGTTCACTCAGCCGAATCAGG<br>CTGCTGGATACATGGCTAAGCTGATTTGGGAATCTGTGAGCGTGACGGTGGTAGCTGCGGTTGAAGCAATGAACTGGCTT<br>AAGTCTGCTGCTAAGCTGCTGGCTGCTGAGGTCAAAGATAAGAAGACTGGAGAGATTCTTCGCAAGCGTTGCGCTGTGCA<br>TTGGGTAACTCCTGATGGTTTCCCTGTGTGGCAGGAATACAAGAAGCCTATTCAGACGCGCTTGAACCTGATGTTCCTCG<br>GTCAGTTCCGCTTACAGCCTACCATTAACACCAACAAAGATAGCGAGATTGATGCACACAAACAGGAGTCTGGTATCGCT<br>CCTAACTTTGTACACAGCCAAGACGGTAGCCACCTTCGTAAGACTGTAGTGTGGGCACACGAGAAGTACGGAATCGAATC<br>TTTTGCACTGATTCACGACTCCTTCGGTACCATTCCGGCTGACGCTGCGAACCTGTTCAAAGCAGTGCGCGAAACTATGG<br>TTGACACATATGAGTCTTGTGATGTACTGGCTGATTTCTACGACCAGTTCGCTGACCAGTTGCACGAGTCTCAATTGGAC<br>AAAATGCCAGCACTTCCGGCTAAAGGTAACTTGAACCTCCGTGACATCTTAGAGTCGGACTTCGCGTTCGCGTAAAGGTT<br>TCAGCCAAAAAACTTAAGACCGCCGGTCTTGTCCACTACCTTGCAGTAATGCGGTGGACAGGATCGGCGGTTTTCTTTTC<br>TCTTCTCAAGCTTGAGCCAACTCCCTTTACAACCTCACTCAAGTCCGTTAGAGGGAGTAATACGACTCACTATAGGGTAC<br>TAGCTGTCACCGGATGTGCTTTCCGGTCTGATGAGTCCGTGAGGACGAAACAGCCTCTACAAATAATTTTGTTTAAAATA<br>ATTTTGTTTAACTTTAAGAAGGAGATATACAATGCGTAAAGGCGAAGAGCTGTTCACTGGTGTCGTCCCTATTCTGGTGG<br>AACTGGATGGTGATGTCAACGGTCATAAGTTTTCCGTGCGTGGCGAGGGTGAAGGTGACGCAACTAATGGTAAACTGACG<br>CTGAAGTTCATCTGTACTACTGGTAAACTGCCGGTACCTTGGCCGACTCTGGTAACGACGCTGACTTATGGTGTTCAGTG<br>CTTTGCTCGTTATCCGGACCATATGAAGCAGCATGACTTCTTCAAGTCCGCCATGCCGGAAGGCTATGTGCAGGAACGCA<br>CGATTTCCTTTAAGGATGACGGCACGTACAAAACGCGTGCGGAAGTGAAATTTGAAGGCGATACCCTGGTAAACCGCATT<br>GAGCTGAAAGGCATTGACTTTAAAGAAGATGGCAATATCCTGGGCCATAAGCTGGAATACAATTTTAACAGCCACAATGT<br>TTACATCACCGCCGATAAACAAAAAAATGGCATTAAAGCGAATTTTAAAATTCGCCACAACGTGGAGGATGGCAGCGTGC<br>AGCTGGCTGATCACTACCAGCAAAACACTCCAATCGGTGATGGTCCTGTTCTGCTGCCAGACAATCACTATCTGAGCACG<br>CAAAGCGTTCTGTCTAAAGATCCGAACGAGAAACGCGATCATATGGTTCTGCTGGAGTTCGTAACCGCAGCGGGCATCAC<br>GCATGGTATGGATGAACTGTACAAATGATGAAGGTGCTGCTGCCACCGCTGAGCAATAACTAGCATAACCCCTTGGGGCC<br>TCTAAACGGGTCTTGAGGGGTTTTTTGCTGAAAGGAGGAACTATAGCTTCCAGGATACATAGATTACCACAACTCCGAGC<br>CCTTCCACC
dCas9, gRNA cassette (Repressor cassette for all D gates)<br><br>UNS 1 - PTet - B0034 - dCas9:GGGSx2 Linker:mScarlet3 - T13m - UNS 6 - POR1OR2 - Hammerhead Ribozyme - gRNA (with variable sequence) - T14m - UNS X	CATTACTCGCATCCATTCTCAGGCTGTCTCGTCTCGTCTCGGAGTCCCTATCAGTGATAGAGATTGACATCCCTATCAGT<br>GATAGAGATACTGAGCACTACTAGAGAAAGAGGAGAAATACTAAATGGATAAGAAATACTCAATAGGCTTAGCTATCGGC<br>ACAAATAGCGTCGGATGGGCGGTGATCACTGATGAATATAAGGTTCCGTCTAAAAAGTTCAAGGTTCTGGGAAATACAGA<br>CCGCCACAGTATCAAAAAAAATCTTATAGGGGCTCTTTTATTTGACAGTGGAGAGACAGCGGAAGCGACTCGTCTCAAAC<br>GGACAGCTCGTAGAAGGTATACACGTCGGAAGAATCGTATTTGTTATCTACAGGAGATTTTTTCAAATGAGATGGCGAAA<br>GTAGATGATAGTTTCTTTCATCGACTTGAAGAGTCTTTTTTGGTGGAAGAAGACAAGAAGCATGAACGTCATCCTATTTT<br>TGGAAATATAGTAGATGAAGTTGCTTATCATGAGAAATATCCAACTATCTATCATCTGCGAAAAAAATTGGTAGATTCTA<br>CTGATAAAGCGGATTTGCGCTTAATCTATTTGGCCTTAGCGCATATGATTAAGTTTCGTGGTCATTTTTTGATTGAGGGA<br>GATTTAAATCCTGATAATAGTGATGTGGACAAACTATTTATCCAGTTGGTACAAACCTACAATCAATTATTTGAAGAAAA<br>CCCTATTAACGCAAGTGGAGTAGATGCTAAAGCGATTCTTTCTGCACGATTGAGTAAATCAAGACGATTAGAAAATCTCA<br>TTGCTCAGCTCCCCGGTGAGAAGAAAAATGGCTTATTTGGGAATCTCATTGCTTTGTCATTGGGTTTGACCCCTAATTTT<br>AAATCAAATTTTGATTTGGCAGAAGATGCTAAATTACAGCTTTCAAAAGATACTTACGATGATGATTTAGATAATTTATT<br>GGCGCAAATTGGAGATCAATATGCTGATTTGTTTTTGGCAGCTAAGAATTTATCAGATGCTATTTTACTTTCAGATATCC<br>TAAGAGTAAATACTGAAATAACTAAGGCTCCCCTATCAGCTTCAATGATTAAACGCTACGATGAACATCATCAAGACTTG<br>ACTCTTTTAAAAGCTTTAGTTCGACAACAACTTCCAGAAAAGTATAAAGAAATCTTTTTTGATCAATCAAAAAACGGATA<br>TGCAGGTTATATTGATGGGGGAGCTAGCCAAGAAGAATTTTATAAATTTATCAAACCAATTTTAGAAAAAATGGATGGTA<br>CTGAGGAATTATTGGTGAAACTAAATCGTGAAGATTTGCTGCGCAAGCAACGGACCTTTGACAACGGCTCTATTCCCCAT<br>CAAATTCACTTGGGTGAGCTGCATGCTATTTTGAGAAGACAAGAAGACTTTTATCCATTTTTAAAAGACAATCGTGAGAA<br>GATTGAAAAAATCTTGACTTTTCGAATTCCTTATTATGTTGGTCCATTGGCGCGTGGCAATAGTCGTTTTGCATGGATGA<br>CTCGGAAGTCTGAAGAAACAATTACCCCATGGAATTTTGAAGAAGTTGTCGATAAAGGTGCTTCAGCTCAATCATTTATT<br>GAACGCATGACAACTTTGATAAAAATCTTCCAAATGAAAAAGTACTACCAAAACATAGTTTGCTTTATGAGTATTTTAC

	GGTTTATAACGAATTGACAAAGGTCAAATATGTTACTGAAGGAATGCGAAAACCAGCATTTCTTTCAGGTGAACAGAAGA<br>AAGCCATTGTTGATTTACTCTTCAAAACAAATCGAAAAGTAACCGTTAAGCAATTAAAAGAAGATTATTTCAAAAAAATA<br>GAATGTTTTGATAGTGTTGAAATTTCAGGAGTTGAAGATAGATTTAATGCTTCATTAGGTACCTACCATGATTTGCTAAA<br>AATTATTAAAGATAAAGATTTTTTGGATAATGAAGAAAATGAAGATATCTTAGAGGATATTGTTTTAACATTGACCTTAT<br>TTGAAGATAGGGAGATGATTGAGGAAAGACTTAAAACATATGCTCACCTCTTTGATGATAAGGTGATGAAACAGCTTAAA<br>CGTCGCCGTTATACTGGTTGGGGACGTTTGTCTCGAAAATTGATTAATGGTATTAGGGATAAGCAATCTGGCAAAACAAT<br>ATTAGATTTTTTGAAATCAGATGGTTTTGCCAATCGCAATTTTATGCAGCTGATCCATGATGATAGTTTGACATTTAAAG<br>AAGACATTCAAAAAGCACAAGTGTCTGGACAAGGCGATAGTTTACATGAACATATTGCAAATTTAGCTGGTAGCCCTGCT<br>ATTAAAAAAGGTATTTTACAGACTGTAAAAGTTGTTGATGAATTGGTCAAAGTAATGGGGCGGCATAAGCCAGAAAATAT<br>CGTTATTGAAATGGCACGTGAAAATCAGACAACTCAAAAGGGCCAGAAAAATTCGCGAGAGCGTATGAAACGAATCGAAG<br>AAGGTATCAAAGAATTAGGAAGTCAGATTCTTAAAGAGCATCCTGTTGAAAATACTCAATTGCAAAATGAAAAGCTCTAT<br>CTCTATTATCTCCAAAATGGAAGAGACATGTATGTGGACCAAGAATTAGATATTAATCGTTTAAGTGATTATGATGTCGA<br>TGCCATTGTTCCACAAAGTTTCCTTAAAGACGATTCAATAGACAATAAGGTCTTAACGCGTTCTGATAAAAATCGTGGTA<br>AATCGGATAACGTTCCAAGTGAAGAAGTAGTCAAAAAGATGAAAAACTATTGGAGACAACTTCTAAACGCCAAGTTAATC<br>ACTCAACGTAAGTTTGATAATTTAACGAAAGCTGAACGTGGAGGTTTGAGTGAACTTGATAAAGCTGGTTTTATCAAACG<br>CCAATTGGTTGAAACTCGCCAAATCACTAAGCATGTGGCACAAATTTTGGATAGTCGCATGAATACTAAATACGATGAAA<br>ATGATAAACTTATTCGAGAGGTTAAAGTGATTACCTTAAAATCTAAATTAGTTTCTGACTTCCGAAAAGATTTCCAATTC<br>TATAAAGTACGTGAGATTAACAATTACCATCATGCCCATGATGCGTATCTAAATGCCGTCGTTGGAACTGCTTTGATTAA<br>GAAATATCCAAAACTTGAATCGGAGTTTGTCTATGGTGATTATAAAGTTTATGATGTTCGTAAAATGATTGCTAAGTCTG<br>AGCAAGAAATAGGCAAAGCAACCGCAAAATATTTCTTTTACTCTAATATCATGAACTTCTTCAAAACAGAAATTACACTT<br>GCAAATGGAGAGATTCGCAAACGCCCTCTAATCGAAACTAATGGGGAAACTGGAGAAATTGTCTGGGATAAAGGGCGAGA<br>TTTTGCCACAGTGCGCAAAGTATTGTCCATGCCCCAAGTCAATATTGTCAAGAAAACAGAAGTACAGACAGGCGGATTCT<br>CCAAGGAGTCAATTTTACCAAAAAGAAATTCGGACAAGCTTATTGCTCGTAAAAAAGACTGGGATCCAAAAAAATATGGT<br>GGTTTTGATAGTCCAACGGTAGCTTATTCAGTCCTAGTGGTTGCTAAGGTGGAAAAAGGGAAATCGAAGAAGTTAAAATC<br>CGTTAAAGAGTTACTAGGGATCACAATTATGGAAAGAAGTTCCTTTGAAAAAAATCCGATTGACTTTTTAGAAGCTAAAG<br>GATATAAGGAAGTTAAAAAAGACTTAATCATTAAACTACCTAAATATAGTCTTTTTGAGTTAGAAAACGGTCGTAAACGG<br>ATGCTGGCTAGTGCCGGAGAATTACAAAAAGGAAATGAGCTGGCTCTGCCAAGCAAATATGTGAATTTTTTATATTTAGC<br>TAGTCATTATGAAAAGTTGAAGGGTAGTCCAGAAGATAACGAACAAAAACAATTGTTTGTGGAGCAGCATAAGCATTATT<br>TAGATGAGATTATTGAGCAAATCAGTGAATTTTCTAAGCGTGTTATTTTAGCAGATGCCAATTTAGATAAAGTTCTTAGT<br>GCATATAACAAACATAGAGACAAACCAATACGTGAACAAGCAGAAAATATTATTCATTTATTTACGTTGACGAATCTTGG<br>AGCTCCCGCTGCTTTTAAATATTTTGATACAACAATTGATCGTAAACGATATACGTCTACAAAAGAAGTTTTAGATGCCA<br>CTCTTATCCATCAATCCATCACTGGTCTTTATGAAACACGCATTGATTTGAGTCAGCTAGGAGGTGACGGTGGTGGTAGC<br>GGTGGTGGTTCAGACTCAACCGAAGCGGTAATCAAGGAGTTTATGCGCTTCAAGGTGCACATGGAGGGGTCAATGAATGG<br>GCACGAGTTCGAGATCGAAGGAGAAGGTGAGGGTCGTCCATACGAAGGCACTCAGACCGCTAAGTTACGTGTCACCAAAG<br>GAGGCCCTTTGCCCTTCTCATGGGATATTTTATCACCACAATTTATGTACGGATCACGTGCATTTACAAAGCATCCGGCT<br>GACATTCCTGATTATTGGAAGCAATCGTTCCCCGAGGGTTTTAAATGGGAGCGTGTGATGAACTTTGAAGATGGGGGCGC<br>CGTCTCAGTTGCTCAGGACACCTCTCTGGAGGATGGTACCCTTATTTATAAGGTAAAGCTGCGTGGAACAAACTTCCCTC<br>CTGACGGCCCTGTCATGCAAAAAAAAACCATGGGGTGGGAAGCGAGTACAGAGCGTTTATATCCCGAGGATGTTGTACTG<br>AAAGGGGACATCAAAATGGCTTTACGTTTGAAGGATGGAGGTCGCTACCTGGCGGACTTTAAGACAACATACCGCGCGAA<br>AAAACCTGTCCAAATGCCCGGCGCTTTTAACATTGACCGTAAGTTGGACATCACAAGTCATAATGAAGATTACACGGTCG<br>TTGAGCAGTACGAACGTAGCGTCGCTCGCCATTCCACCGGCGGAAGTGGGGGTAGTTAAAGGTGGAAACACAGAAAAAAG<br>CCCGCACCTGACAGTGCGGGCTTTTTTTTTCGACCAAAGGGCTTCTCGTTCGCTGCCACCTAAGAATACTCTACGGTCAC<br>ATACGGAGTGCTGTTCCGCTGGGCATGCTGAGCTAACACCGTGCGTGTTGACAATTTACCTCTGGCGGTGATAATGGTT<br>GCAGCTACTAGTAGCCTGATGAGTCCGTGAGGACGAAACGAGTAAGCTCGTCGCTACTNNNNNNNNNNNNNNNNNNNNGT<br>TTTAGAGCTAGAAATAGCAAGTTAAAATAAGGCTAGTCCGTTATCAACTTGAAAAAGTGGCACCGAGTCGGTGCTTTTTT<br>TAGGTGACGAACAATAAGGCCTCCCTAACGGGGGGCCTTTTTTATTGATAACAAAAGCTTCCAGGATACATAGATTACCA<br>CAACTCCGAGCCCTTCCACC
D^{0} gRNA	AACAAACGCAGAATCCAAGC
D^{T}_{-28} / F^{T}_{-50} / P^{T}_{-32} gRNA	CCTCACTCAAGTCCGTTAGA
D^{C}_{-23} / F^{C}_{-34} / P^{C}_{-52} gRNA*	AGTCGTATTACTCCCTCTAA
D^{T}_{-6} / F^{T}_{12} / P^{T}_{30} gRNA	GGAGTAATACGACTCACTAT
D^{T}_{12} gRNA	TATAGGGTACTAGCTGTCAC
D^{C}_{23} / F^{C}_{36} / P^{C}_{18} gRNA	ATCAGACCGGAAAGCACATC
D^{T}_{25} gRNA	CTGTCACCGGATGTGCTTTC
D^{T}_{43} gRNA	TCCGGTCTGATGAGTCCGTG
D^{C}_{50} gRNA	TAGAGGCTGTTTCGTCCTCA
D^{C}_{67} gRNA	TTAAACAAAATTATTTGTAG
D^{T}_{101} gRNA	AATTTTGTTTAACTTTAAGA

D^{T}_{156} gRNA	CACTGGTGTCGTCCCTATTC
D^{C}_{160} gRNA	CATCCAGTTCCACCAGAATA
F^{T}_{94} / P^{T}_{112} gRNA	TCTACAAATAATTTTGTTTA
F^{C}_{117} / P^{C}_{97} gRNA	TAAAGTTAAACAAAATTATT
T7, GFP cassette for F^{T}_{-50} and P^{T}_{-32}<br><br>UNS 1 - PLacO - B0033 - T7 RNAP - T12m - UNS 5 - spacer - PhlO (rev. comp.) - PT7 - RiboJ - UTR1 - sfGFP - T7 Terminator - UNS X	[Identical sequence to PLacO T7 RNAP expression cassette for D gates]GAGCCAACTCCCTTTACAACCTCACTCAAGTCCGTTAGAGGGAGGAATCCAAGCACCTTAACGATACGGTAC<br>GTTTCGTATCATTAATACGACTCACTATAGGGTACTAGCTGTCACCGGATGTGCTTTCCGGTCTGATGAGTCCGTGAGGA<br>CGAAACAGCCTCTACAAATAATTTTGTTTAAAATAATTTTGTTTAACTTTAAGAAGGAGATATACAATGCGTAAAGGCGA<br>AGAGCTGTTCACTGGTGTCGTCCCTATTCTGGTGGAACTGGATGGTGATGTCAACGGTCATAAGTTTTCCGTGCGTGGCG<br>AGGGTGAAGGTGACGCAACTAATGGTAAACTGACGCTGAAGTTCATCTGTACTACTGGTAAACTGCCGGTACCTTGGCCG<br>ACTCTGGTAACGACGCTGACTTATGGTGTTCAGTGCTTTGCTCGTTATCCGGACCATATGAAGCAGCATGACTTCTTCAA<br>GTCCGCCATGCCGGAAGGCTATGTGCAGGAACGCACGATTTCCTTTAAGGATGACGGCACGTACAAAACGCGTGCGGAAG<br>TGAAATTTGAAGGCGATACCCTGGTAAACCGCATTGAGCTGAAAGGCATTGACTTTAAAGAAGATGGCAATATCCTGGGC<br>CATAAGCTGGAATACAATTTTAACAGCCACAATGTTTACATCACCGCCGATAAACAAAAAAATGGCATTAAAGCGAATTT<br>TAAAATTCGCCACAACGTGGAGGATGGCAGCGTGCAGCTGGCTGATCACTACCAGCAAAACACTCCAATCGGTGATGGTC<br>CTGTTCTGCTGCCAGACAATCACTATCTGAGCACGCAAAGCGTTCTGTCTAAAGATCCGAACGAGAAACGCGATCATATG<br>GTTCTGCTGGAGTTCGTAACCGCAGCGGGCATCACGCATGGTATGGATGAACTGTACAAATGATGAAGGTGCTGCTGCCA<br>CCGCTGAGCAATAACTAGCATAACCCCTTGGGGCCTCTAAACGGGTCTTGAGGGGTTTTTTGCTGAAAGGAGGAACTATA<br>GCTTCCAGGATACATAGATTACCACAACTCCGAGCCCTTCCACC
T7, GFP cassette for F^{C}_{-34} and P^{C}_{-52}<br><br>UNS 1 - PLacO - B0033 - T7 RNAP - T12m - UNS 5 - PhlO - - spacer - PT7 - RiboJ - UTR1 - sfGFP - T7 Terminator - UNS X	[Identical sequence to PLacO T7 RNAP expression cassette for D gates]GAGCCAACTCCCTTTACAACCTCACTCAAGTCCGTTAGAGATGATACGAAACGTACCGTATCGTTAAGGTCA<br>GAATCCAAGCCCGTAATACGACTCACTATAGGGTACTAGCTGTCACCGGATGTGCTTTCCGGTCTGATGAGTCCGTGAGG<br>ACGAAACAGCCTCTACAAATAATTTTGTTTAAAATAATTTTGTTTAACTTTAAGAAGGAGATATACAATGCGTAAAGGCG<br>AAGAGCTGTTCACTGGTGTCGTCCCTATTCTGGTGGAACTGGATGGTGATGTCAACGGTCATAAGTTTTCCGTGCGTGGC<br>GAGGGTGAAGGTGACGCAACTAATGGTAAACTGACGCTGAAGTTCATCTGTACTACTGGTAAACTGCCGGTACCTTGGCC<br>GACTCTGGTAACGACGCTGACTTATGGTGTTCAGTGCTTTGCTCGTTATCCGGACCATATGAAGCAGCATGACTTCTTCA<br>AGTCCGCCATGCCGGAAGGCTATGTGCAGGAACGCACGATTTCCTTTAAGGATGACGGCACGTACAAAACGCGTGCGGAA<br>GTGAAATTTGAAGGCGATACCCTGGTAAACCGCATTGAGCTGAAAGGCATTGACTTTAAAGAAGATGGCAATATCCTGGG<br>CCATAAGCTGGAATACAATTTTAACAGCCACAATGTTTACATCACCGCCGATAAACAAAAAAATGGCATTAAAGCGAATT<br>TTAAAATTCGCCACAACGTGGAGGATGGCAGCGTGCAGCTGGCTGATCACTACCAGCAAAACACTCCAATCGGTGATGGT<br>CCTGTTCTGCTGCCAGACAATCACTATCTGAGCACGCAAAGCGTTCTGTCTAAAGATCCGAACGAGAAACGCGATCATAT<br>GGTTCTGCTGGAGTTCGTAACCGCAGCGGGCATCACGCATGGTATGGATGAACTGTACAAATGATGAAGGTGCTGCTGCC<br>ACCGCTGAGCAATAACTAGCATAACCCCTTGGGGCCTCTAAACGGGTCTTGAGGGGTTTTTTGCTGAAAGGAGGAACTAT<br>AGCTTCCAGGATACATAGATTACCACAACTCCGAGCCCTTCCACC
T7, GFP cassette for F^{T}_{12} and P^{T}_{30}<br><br>UNS 1 - PLacO - B0033 - T7 RNAP - T12m - UNS 5 - PT7 - spacer - PhlO (rev. comp.) - RiboJ - UTR1 - sfGFP - T7 Terminator - UNS X	[Identical sequence to PLacO T7 RNAP expression cassette for D gates]GAGCCAACTCCCTTTACAACCTCACTCAAGTCCGTTAGAGGGAGTAATACGACTCACTATAGGGTACTTCCA<br>AGCACCTTAACGATACGGTACGTTTCGTATCATAGCTGTCACCGGATGTGCTTTCCGGTCTGATGAGTCCGTGAGGACGA<br>AACAGCCTCTACAAATAATTTTGTTTAAAATAATTTTGTTTAACTTTAAGAAGGAGATATACAATGCGTAAAGGCGAAGA<br>GCTGTTCACTGGTGTCGTCCCTATTCTGGTGGAACTGGATGGTGATGTCAACGGTCATAAGTTTTCCGTGCGTGGCGAGG<br>GTGAAGGTGACGCAACTAATGGTAAACTGACGCTGAAGTTCATCTGTACTACTGGTAAACTGCCGGTACCTTGGCCGACT<br>CTGGTAACGACGCTGACTTATGGTGTTCAGTGCTTTGCTCGTTATCCGGACCATATGAAGCAGCATGACTTCTTCAAGTC<br>CGCCATGCCGGAAGGCTATGTGCAGGAACGCACGATTTCCTTTAAGGATGACGGCACGTACAAAACGCGTGCGGAAGTGA<br>AATTTGAAGGCGATACCCTGGTAAACCGCATTGAGCTGAAAGGCATTGACTTTAAAGAAGATGGCAATATCCTGGGCCAT<br>AAGCTGGAATACAATTTTAACAGCCACAATGTTTACATCACCGCCGATAAACAAAAAAATGGCATTAAAGCGAATTTTAA<br>AATTCGCCACAACGTGGAGGATGGCAGCGTGCAGCTGGCTGATCACTACCAGCAAAACACTCCAATCGGTGATGGTCCTG<br>TTCTGCTGCCAGACAATCACTATCTGAGCACGCAAAGCGTTCTGTCTAAAGATCCGAACGAGAAACGCGATCATATGGTT<br>CTGCTGGAGTTCGTAACCGCAGCGGGCATCACGCATGGTATGGATGAACTGTACAAATGATGAAGGTGCTGCTGCCACCG<br>CTGAGCAATAACTAGCATAACCCCTTGGGGCCTCTAAACGGGTCTTGAGGGGTTTTTTGCTGAAAGGAGGAACTATAGCT<br>TCCAGGATACATAGATTACCACAACTCCGAGCCCTTCCACC
T7, GFP cassette for F^{C}_{36} and P^{C}_{18}<br><br>UNS 1 - PLacO - B0033 - T7 RNAP - T12m - UNS 5 - PT7 - PhlO - RiboJ - UTR1 - sfGFP - T7 Terminator - UNS X	[Identical sequence to PLacO T7 RNAP expression cassette for D gates]GAGCCAACTCCCTTTACAACCTCACTCAAGTCCGTTAGAGGGAGTAATACGACTCACTATAGGGATGATACG<br>AAACGTACCGTATCGTTAAGGTTACTAGCTGTCACCGGATGTGCTTTCCGGTCTGATGAGTCCGTGAGGACGAAACAGCC<br>TCTACAAATAATTTTGTTTAAAATAATTTTGTTTAACTTTAAGAAGGAGATATACAATGCGTAAAGGCGAAGAGCTGTTC<br>ACTGGTGTCGTCCCTATTCTGGTGGAACTGGATGGTGATGTCAACGGTCATAAGTTTTCCGTGCGTGGCGAGGGTGAAGG<br>TGACGCAACTAATGGTAAACTGACGCTGAAGTTCATCTGTACTACTGGTAAACTGCCGGTACCTTGGCCGACTCTGGTAA<br>CGACGCTGACTTATGGTGTTCAGTGCTTTGCTCGTTATCCGGACCATATGAAGCAGCATGACTTCTTCAAGTCCGCCATG<br>CCGGAAGGCTATGTGCAGGAACGCACGATTTCCTTTAAGGATGACGGCACGTACAAAACGCGTGCGGAAGTGAAATTTGA<br>AGGCGATACCCTGGTAAACCGCATTGAGCTGAAAGGCATTGACTTTAAAGAAGATGGCAATATCCTGGGCCATAAGCTGG<br>AATACAATTTTAACAGCCACAATGTTTACATCACCGCCGATAAACAAAAAAATGGCATTAAAGCGAATTTTAAAATTCGC<br>CACAACGTGGAGGATGGCAGCGTGCAGCTGGCTGATCACTACCAGCAAAACACTCCAATCGGTGATGGTCCTGTTCTGCT<br>GCCAGACAATCACTATCTGAGCACGCAAAGCGTTCTGTCTAAAGATCCGAACGAGAAACGCGATCATATGGTTCTGCTGG<br>AGTTCGTAACCGCAGCGGGCATCACGCATGGTATGGATGAACTGTACAAATGATGAAGGTGCTGCTGCCACCGCTGAGCA<br>ATAACTAGCATAACCCCTTGGGGCCTCTAAACGGGTCTTGAGGGGTTTTTTGCTGAAAGGAGGAACTATAGCTTCCAGGA

	TACATAGATTACCACAACTCCGAGCCCTTCCACC
T7, GFP cassette for F^{T}_{94} and P^{T}_{112}<br><br>UNS 1 - PLacO - B0033 - T7 RNAP - T12m - UNS 5 - PT7 - RiboJ - spacer - PhlO (rev. comp.) - UTR1 - sfGFP - T7 Terminator - UNS X	[Identical sequence to PLacO T7 RNAP expression cassette for D gates]GAGCCAACTCCCTTTACAACCTCACTCAAGTCCGTTAGAGGGAGTAATACGACTCACTATAGGGTACTAGCT<br>GTCACCGGATGTGCTTTCCGGTCTGATGAGTCCGTGAGGACGAAACAGCCTCTACAAATAATTTTGTTTAAGGCAGAATC<br>CAAGCACCTTAACGATACGGTACGTTTCGTATCATAATAATTTTGTTTAACTTTAAGAAGGAGATATACAATGCGTAAAG<br>GCGAAGAGCTGTTCACTGGTGTCGTCCCTATTCTGGTGGAACTGGATGGTGATGTCAACGGTCATAAGTTTTCCGTGCGT<br>GGCGAGGGTGAAGGTGACGCAACTAATGGTAAACTGACGCTGAAGTTCATCTGTACTACTGGTAAACTGCCGGTACCTTG<br>GCCGACTCTGGTAACGACGCTGACTTATGGTGTTCAGTGCTTTGCTCGTTATCCGGACCATATGAAGCAGCATGACTTCT<br>TCAAGTCCGCCATGCCGGAAGGCTATGTGCAGGAACGCACGATTTCCTTTAAGGATGACGGCACGTACAAAACGCGTGCG<br>GAAGTGAAATTTGAAGGCGATACCCTGGTAAACCGCATTGAGCTGAAAGGCATTGACTTTAAAGAAGATGGCAATATCCT<br>GGGCCATAAGCTGGAATACAATTTTAACAGCCACAATGTTTACATCACCGCCGATAAACAAAAAAATGGCATTAAAGCGA<br>ATTTTAAAATTCGCCACAACGTGGAGGATGGCAGCGTGCAGCTGGCTGATCACTACCAGCAAAACACTCCAATCGGTGAT<br>GGTCCTGTTCTGCTGCCAGACAATCACTATCTGAGCACGCAAAGCGTTCTGTCTAAAGATCCGAACGAGAAACGCGATCA<br>TATGGTTCTGCTGGAGTTCGTAACCGCAGCGGGCATCACGCATGGTATGGATGAACTGTACAAATGATGAAGGTGCTGCT<br>GCCACCGCTGAGCAATAACTAGCATAACCCCTTGGGGCCTCTAAACGGGTCTTGAGGGGTTTTTTGCTGAAAGGAGGAAC<br>TATAGCTTCCAGGATACATAGATTACCACAACTCCGAGCCCTTCCACC
T7, GFP cassette for F^{C}_{115} and P^{C}_{97}<br><br>UNS 1 - PLacO - B0033 - T7 RNAP - T12m - UNS 5 - PT7 - RiboJ - PhlO - spacer - UTR1 - sfGFP - T7 Terminator - UNS X	[Identical sequence to PLacO T7 RNAP expression cassette for D gates]GAGCCAACTCCCTTTACAACCTCACTCAAGTCCGTTAGAGGGAGTAATACGACTCACTATAGGGTACTAGCT<br>GTCACCGGATGTGCTTTCCGGTCTGATGAGTCCGTGAGGACGAAACAGCCTCTACAAATAATTTTGTTTAAATGATACGA<br>AACGTACCGTATCGTTAAGGTCAGAATCCAAGCCCGAATAATTTTGTTTAACTTTAAGAAGGAGATATACAATGCGTAAA<br>GGCGAAGAGCTGTTCACTGGTGTCGTCCCTATTCTGGTGGAACTGGATGGTGATGTCAACGGTCATAAGTTTTCCGTGCG<br>TGGCGAGGGTGAAGGTGACGCAACTAATGGTAAACTGACGCTGAAGTTCATCTGTACTACTGGTAAACTGCCGGTACCTT<br>GGCCGACTCTGGTAACGACGCTGACTTATGGTGTTCAGTGCTTTGCTCGTTATCCGGACCATATGAAGCAGCATGACTTC<br>TTCAAGTCCGCCATGCCGGAAGGCTATGTGCAGGAACGCACGATTTCCTTTAAGGATGACGGCACGTACAAAACGCGTGC<br>GGAAGTGAAATTTGAAGGCGATACCCTGGTAAACCGCATTGAGCTGAAAGGCATTGACTTTAAAGAAGATGGCAATATCC<br>TGGGCCATAAGCTGGAATACAATTTTAACAGCCACAATGTTTACATCACCGCCGATAAACAAAAAAATGGCATTAAAGCG<br>AATTTTAAAATTCGCCACAACGTGGAGGATGGCAGCGTGCAGCTGGCTGATCACTACCAGCAAAACACTCCAATCGGTGA<br>TGGTCCTGTTCTGCTGCCAGACAATCACTATCTGAGCACGCAAAGCGTTCTGTCTAAAGATCCGAACGAGAAACGCGATC<br>ATATGGTTCTGCTGGAGTTCGTAACCGCAGCGGGCATCACGCATGGTATGGATGAACTGTACAAATGATGAAGGTGCTGC<br>TGCCACCGCTGAGCAATAACTAGCATAACCCCTTGGGGCCTCTAAACGGGTCTTGAGGGGTTTTTTGCTGAAAGGAGGAA<br>CTATAGCTTCCAGGATACATAGATTACCACAACTCCGAGCCCTTCCACC
dCas9*:PhlF, gRNA cassette for all F gates<br><br>UNS 1 - PTet - B0034 - dCas9*:linker:PhlF:GGGSx2 Linker:mScarlet3 - T13m - UNS 6 - POR1OR2 - Hammerhead Ribozyme - gRNA (with variable sequence) - T14m - UNS X	CATTACTCGCATCCATTCTCAGGCTGTCTCGTCTCGTCTCGGAGTCCCTATCAGTGATAGAGATTGACATCCCTATCAGT<br>GATAGAGATACTGAGCACTACTAGAGAAAGAGGAGAAATACTAAATGGATAAGAAATACTCAATAGGCTTAGCTATCGGC<br>ACAAATAGCGTCGGATGGGCGGTGATCACTGATGAATATAAGGTTCCGTCTAAAAAGTTCAAGGTTCTGGGAAATACAGA<br>CCGCCACAGTATCAAAAAAAATCTTATAGGGGCTCTTTTATTTGACAGTGGAGAGACAGCGGAAGCGACTCGTCTCAAAC<br>GGACAGCTCGTAGAAGGTATACACGTCGGAAGAATCGTATTTGTTATCTACAGGAGATTTTTTCAAATGAGATGGCGAAA<br>GTAGATGATAGTTTCTTTCATCGACTTGAAGAGTCTTTTTTGGTGGAAGAAGACAAGAAGCATGAACGTCATCCTATTTT<br>TGGAAATATAGTAGATGAAGTTGCTTATCATGAGAAATATCCAACTATCTATCATCTGCGAAAAAAATTGGTAGATTCTA<br>CTGATAAAGCGGATTTGCGCTTAATCTATTTGGCCTTAGCGCATATGATTAAGTTTCGTGGTCATTTTTTGATTGAGGGA<br>GATTTAAATCCTGATAATAGTGATGTGGACAAACTATTTATCCAGTTGGTACAAACCTACAATCAATTATTTGAAGAAAA<br>CCCTATTAACGCAAGTGGAGTAGATGCTAAAGCGATTCTTTCTGCACGATTGAGTAAATCAAGACGATTAGAAAATCTCA<br>TTGCTCAGCTCCCCGGTGAGAAGAAAAATGGCTTATTTGGGAATCTCATTGCTTTGTCATTGGGTTTGACCCCTAATTTT<br>AAATCAAATTTTGATTTGGCAGAAGATGCTAAATTACAGCTTTCAAAAGATACTTACGATGATGATTTAGATAATTTATT<br>GGCGCAAATTGGAGATCAATATGCTGATTTGTTTTTGGCAGCTAAGAATTTATCAGATGCTATTTTACTTTCAGATATCC<br>TAAGAGTAAATACTGAAATAACTAAGGCTCCCCTATCAGCTTCAATGATTAAACGCTACGATGAACATCATCAAGACTTG<br>ACTCTTTTAAAAGCTTTAGTTCGACAACAACTTCCAGAAAAGTATAAAGAAATCTTTTTTGATCAATCAAAAAACGGATA<br>TGCAGGTTATATTGATGGGGGAGCTAGCCAAGAAGAATTTTATAAATTTATCAAACCAATTTTAGAAAAAATGGATGGTA<br>CTGAGGAATTATTGGTGAAACTAAATCGTGAAGATTTGCTGCGCAAGCAACGGACCTTTGACAACGGCTCTATTCCCCAT<br>CAAATTCACTTGGGTGAGCTGCATGCTATTTTGAGAAGACAAGAAGACTTTTATCCATTTTTAAAAGACAATCGTGAGAA<br>GATTGAAAAAATCTTGACTTTTCGAATTCCTTATTATGTTGGTCCATTGGCGCGTGGCAATAGTCGTTTTGCATGGATGA<br>CTCGGAAGTCTGAAGAAACAATTACCCCATGGAATTTTGAAGAAGTTGTCGATAAAGGTGCTTCAGCTCAATCATTTATT<br>GAACGCATGACAAACTTTGATAAAAATCTTCCAAATGAAAAAGTACTACCAAAACATAGTTTGCTTTATGAGTATTTTAC<br>GGTTTATAACGAATTGACAAAGGTCAAATATGTTACTGAAGGAATGCGAAAACCAGCATTTCTTTCAGGTGAACAGAAGA<br>AAGCCATTGTTGATTTACTCTTCAAAACAAATCGAAAAGTAACCGTTAAGCAATTAAAAGAAGATTATTTCAAAAAAATA<br>GAATGTTTTGATAGTGTTGAAATTTCAGGAGTTGAAGATAGATTTAATGCTTCATTAGGTACCTACCATGATTTGCTAAA<br>AATTATTAAAGATAAAGATTTTTTGGATAATGAAGAAAATGAAGATATCTTAGAGGATATTGTTTTAACATTGACCTTAT<br>TTGAAGATAGGGAGATGATTGAGGAAAGACTTAAAACATATGCTCACCTCTTTGATGATAAGGTGATGAAACAGCTTAAA<br>CGTCGCCGTTATACTGGTTGGGGACGTTTGTCTCGAAAATTGATTAATGGTATTAGGGATAAGCAATCTGGCAAAACAAT<br>ATTAGATTTTTTGAAATCAGATGGTTTTGCCAATCGCAATTTTATGCAGCTGATCCATGATGATAGTTTGACATTTAAAG<br>AAGACATTCAAAAAGCACAAGTGTCTGGACAAGGCGATAGTTTACATGAACATATTGCAAATTTAGCTGGTAGCCCTGCT<br>ATTAAAAAAGGTATTTTACAGACTGTAAAAGTTGTTGATGAATTGGTCAAAGTAATGGGGCGGCATAAGCCAGAAAATAT<br>CGTTATTGAAATGGCACGTGAAAATCAGACAACTCAAAAGGGCCAGAAAAATTCGCGAGAGCGTATGAAACGAATCGAAG<br>AAGGTATCAAAGAATTAGGAAGTCAGATTCTTAAAGAGCATCCTGTTGAAAATACTCAATTGCAAAATGAAAAGCTCTAT<br>CTCTATTATCTCCAAAATGGAAGAGACATGTATGTGGACCAAGAATTAGATATTAATCGTTTAAGTGATTATGATGTCGA<br>TGCCATTGTTCCACAAAGTTTCCTTAAAGACGATTCAATAGACAATAAGGTCTTAACGCGTTCTGATAAAAATCGTGGTA<br>AATCGGATAACGTTCCAAGTGAAGAAGTAGTCAAAAAGATGAAAAACTATTGGAGACAACTTCTAAACGCCAAGTTAATC<br>ACTCAACGTAAGTTTGATAATTTAACGAAAGCTGAACGTGGAGGTTTGAGTGAACTTGATAAAGCTGGTTTTATCAAACG<br>CCAATTGGTTGAAACTCGCCAAATCACTAAGCATGTGGCACAAATTTTGGATAGTCGCATGAATACTAAATACGATGAAA<br>ATGATAAACTTATTCGAGAGGTTAAAGTGATTACCTTAAAATCTAAATTAGTTTCTGACTTCCGAAAAGATTTCCAATTC<br>TATAAAGTACGTGAGATTAACAATTACCATCATGCCCATGATGCGTATCTAAATGCCGTCGTTGGAACTGCTTTGATTAA<br>GAAATATCCAAAACTTGAATCGGAGTTTGTCTATGGTGATTATAAAGTTTATGATGTTCGTAAAATGATTGCTAAGTCTG

	AGCAAGAAATAGGCAAAGCAACCGCAAAATATTTCTTTTACTCTAATATCATGAACTTCTTCAAAACAGAAATTACACTT<br>GCAAATGGAGAGATTCGCAAACGCCCTCTAATCGAAACTAATGGGGAAACTGGAGAAATTGTCTGGGATAAAGGGCGAGA<br>TTTTGCCACAGTGCGCAAAGTATTGTCCATGCCCCAAGTCAATATTGTCAAGAAAACAGAAGTACAGACAGGCGGATTCT<br>CCAAGGAGTCAATTTTACCAAAAAGAAATTCGGACAAGCTTATTGCTCGTAAAAAAGACTGGGATCCAAAAAAATATGGT<br>GGTTTTGATAGTCCAACGGTAGCTTATTCAGTCCTAGTGGTTGCTAAGGTGGAAAAAGGGAAATCGAAGAAGTTAAAATC<br>CGTTAAAGAGTTACTAGGGATCACAATTATGGAAAGAAGTTCCTTTGAAAAAAATCCGATTGACTTTTTAGAAGCTAAAG<br>GATATAAGGAAGTTAAAAAAGACTTAATCATTAAACTACCTAAATATAGTCTTTTTGAGTTAGAAAACGGTCGTAAACGG<br>ATGCTGGCTAGTGCCGGAGAATTACAAAAAGGAAATGAGCTGGCTCTGCCAAGCAAATATGTGAATTTTTTATATTTAGC<br>TAGTCATTATGAAAAGTTGAAGGGTAGTCCAGAAGATAACGAACAAAAACAATTGTTTGTGGAGCAGCATAAGCATTATT<br>TAGATGAGATTATTGAGCAAATCAGTGAATTTTCTAAGCGTGTTATTTTAGCAGATGCCAATTTAGATAAAGTTCTTAGT<br>GCATATAACAAACATAGAGACAAACCAATACGTGAACAAGCAGAAAATATTATTCATTTATTTACGTTGACGAATCTTGG<br>AGCTCCCGCTGCTTTTAAATATTTTGATACAACAATTGATCGTAAAAAGTATACGTCTACAAAAGAAGTTTTAGATGCCA<br>CTCTTATCCATCAATCCATCACTGGTCTTTATGAAACACGCATTGATTTGAGTCAGCTAGGAGGTGACGGCACCGGCGGG<br>CCCAAGAAGAAGAGGAAGGTATACCCATACGATGTTCCTGACTATGCGGGCTATCCCTATGACGTCCCGGACTATGCAGG<br>ATCGTATCCTTATGACGTTCCAGATTACGCTGGATCCGCCGCTCCGGCAGCTAAGAAAAAGAAACTGGATTACCCGTATG<br>ACGTACCTGATTACGCTGGTTATCCCTATGATGTCCCGGACTACGCTGGCTCGTACCCTTATGATGTACCTGACTACGCT<br>TTCGAATCCGGAGCACGTACCCCGAGCCGTAGCAGCATTGGTAGCCTGCGTAGTCCGCATACCCATAAAGCAATTCTGAC<br>CAGCACCATTGAAATCCTGAAAGAATGTGGTTATAGCGGTCTGAGCATTGAAAGCGTTGCACGTCGTGCCGGTGCAAGCA<br>AACCGACCATTTATCGTTGGTGGACCAATAAAGCAGCACTGATTGCCGAAGTGTATGAAAATGAAAGCGAACAGGTGCGT<br>AAATTTCCGGATCTGGGTAGCTTTAAAGCCGATCTGGATTTTCTGCTGCGTAATCTGTGGAAAGTTTGGCGTGAAACCAT<br>TTGTGGTGAAGCATTTCGTTGTGTTATTGCAGAAGCACAGCTGGACCCTGCAACCCTGACCCAGCTGAAAGATCAGTTTA<br>TGGAACGTCGTCGTGAGATGCCGAAAAAACTGGTTGAAAATGCCATTAGCAATGGTGAACTGCCGAAAGATACCAATCGT<br>GAACTGCTGCTGGATATGATTTTTGGTTTTTGTTGGTATCGCCTGCTGACCGAACAGCTGACCGTTGAACAGGATATTGA<br>AGAATTTACCTTCCTGCTGATTAATGGTGTTTGTCCGGGTACACAGCGTGGTGGTGGTAGCGGTGGTGGTTCAGACTCAA<br>CCGAAGCGGTAATCAAGGAGTTTATGCGCTTCAAGGTGCACATGGAGGGGTCAATGAATGGGCACGAGTTCGAGATCGAA<br>GGAGAAGGTGAGGGTCGTCCATACGAAGGCACTCAGACCGCTAAGTTACGTGTCACCAAAGGAGGCCCTTTGCCCTTCTC<br>ATGGGATATTTTATCACCACAATTTATGTACGGATCACGTGCATTTACAAAGCATCCGGCTGACATTCCTGATTATTGGA<br>AGCAATCGTTCCCCGAGGGTTTTAAATGGGAGCGTGTGATGAACTTTGAAGATGGGGGCGCCGTCTCAGTTGCTCAGGAC<br>ACCTCTCTGGAGGATGGTACCCTTATTTATAAGGTAAAGCTGCGTGGAACAAACTTCCCTCCTGACGGCCCTGTCATGCA<br>AAAAAAAACCATGGGGTGGGAAGCGAGTACAGAGCGTTTATATCCCGAGGATGTTGTACTGAAAGGGGACATCAAAATGG<br>CTTTACGTTTGAAGGATGGAGGTCGCTACCTGGCGGACTTTAAGACAACATACCGCGCGAAAAAACCTGTCCAAATGCCC<br>GGCGCTTTTAACATTGACCGTAAGTTGGACATCACAAGTCATAATGAAGATTACACGGTCGTTGAGCAGTACGAACGTAG<br>CGTCGCTCGCCATTCCACCGGCGGAAGTGGGGGTAGTTAAAGGTGGAAACACAGAAAAAAGCCCGCACCTGACAGTGCGG<br>GCTTTTTTTTTCGACCAAAGGGCTTCTCGTTCGCTGCCACCTAAGAATACTCTACGGTCACATACGGAGTGCTGTTCCGC<br>TGGGCATGCTGAGCTAACACCGTGCGTGTTGACAATTTTACCTCTGGCGGTGATAATGGTTGCAGCTACTAGTAGCCTGA<br>TGAGTCCGTGAGGACGAAACGAGTAAGCTCGTCGCTACTNNNNNNNNNNNNNNNNNNNNGTTTTAGAGCTAGAAATAGCA<br>AGTTAAAATAAGGCTAGTCCGTTATCAACTTGAAAAAGTGGCACCGAGTCGGTGCTTTTTTTAGGTGACGAACAATAAGG<br>CCTCCCTAACGGGGGGCCTTTTTTATTGATAACAAAAGCTTCCAGGATACATAGATTACCACAACTCCGAGCCCTTCCAC<br>C
PhlF, gRNA cassette for all P gates<br><br>UNS 1 - PTet - B0034 - PhlF:GGGSx2 Linker:mScarlet3 - T13m - UNS 6 - POR1OR2 - Hammerhead Ribozyme - gRNA (with variable sequence) - T14m - UNS X	CATTACTCGCATCCATTCTCAGGCTGTCTCGTCTCGTCTCGGAGTCCCTATCAGTGATAGAGATTGACATCCCTATCAGT<br>GATAGAGATACTGAGCACTACTAGAGAAAGAGGAGAAATACTAAGCACGTACCCCGAGCCGTAGCAGCATTGGTAGCCTG<br>CGTAGTCCGCATACCCATAAAGCAATTCTGACCAGCACCATTGAAATCCTGAAAGAATGTGGTTATAGCGGTCTGAGCAT<br>TGAAAGCGTTGCACGTCGTGCCGGTGCAAGCAAACCGACCATTTATCGTTGGTGGACCAATAAAGCAGCACTGATTGCCG<br>AAGTGTATGAAAATGAAAGCGAACAGGTGCGTAAATTTCCGGATCTGGGTAGCTTTAAAGCCGATCTGGATTTTCTGCTG<br>CGTAATCTGTGGAAAGTTTGGCGTGAAACCATTTGTGGTGAAGCATTTCGTTGTGTTATTGCAGAAGCACAGCTGGACCC<br>TGCAACCCTGACCCAGCTGAAAGATCAGTTTATGGAACGTCGTCGTGAGATGCCGAAAAAACTGGTTGAAAATGCCATTA<br>GCAATGGTGAACTGCCGAAAGATACCAATCGTGAACTGCTGCTGGATATGATTTTTGGTTTTTGTTGGTATCGCCTGCTG<br>ACCGAACAGCTGACCGTTGAACAGGATATTGAAGAATTTACCTTCCTGCTGATTAATGGTGTTTGTCCGGGTACACAGCG<br>TGGTGGTGGTAGCGGTGGTGGTTCAGACTCAACCGAAGCGGTAATCAAGGAGTTTATGCGCTTCAAGGTGCACATGGAGG<br>GGTCAATGAATGGGCACGAGTTCGAGATCGAAGGAGAAGGTGAGGGTCGTCCATACGAAGGCACTCAGACCGCTAAGTTA<br>CGTGTCACCAAAGGAGGCCCTTTGCCCTTCTCATGGGATATTTTATCACCACAATTTATGTACGGATCACGTGCATTTAC<br>AAAGCATCCGGCTGACATTCCTGATTATTGGAAGCAATCGTTCCCCGAGGGTTTTAAATGGGAGCGTGTGATGAACTTTG<br>AAGATGGGGGCGCCGTCTCAGTTGCTCAGGACACCTCTCTGGAGGATGGTACCCTTATTTATAAGGTAAAGCTGCGTGGA<br>ACAAACTTCCCTCCTGACGGCCCTGTCATGCAAAAAAAAACCATGGGGTGGGAAGCGAGTACAGAGCGTTTATATCCCGA<br>GGATGTTGTACTGAAAGGGGACATCAAAATGGCTTTACGTTTGAAGGATGGAGGTCGCTACCTGGCGGACTTTAAGACAA<br>CATACCGCGCGAAAAAACCTGTCCAAATGCCCGGCGCTTTTAACATTGACCGTAAGTTGGACATCACAAGTCATAATGAA<br>GATTACACGGTCGTTGAGCAGTACGAACGTAGCGTCGCTCGCCATTCCACCGGCGGAAGTGGGGGTAGTTAAAGGTGGAA<br>ACACAGAAAAAAGCCCGCACCTGACAGTGCGGGCTTTTTTTTTCGACCAAAGGGCTTCTCGTTCGCTGCCACCTAAGAAT<br>ACTCTACGGTCACATACGGAGTGCTGTTCCGCTGGGCATGCTGAGCTAACACCGTGCGTGTTGACAATTTTACCTCTGGC<br>GGTGATAATGGTTGCAGCTACTAGTAGCCTGATGAGTCCGTGAGGACGAAACGAGTAAGCTCGTCGCTACTNNNNNNNNN<br>NNNNNNNNNNNGTTTTAGAGCTAGAAATAGCAAGTTAAAATAAGGCTAGTCCGTTATCAACTTGAAAAAGTGGCACCGAG<br>TCGGTGCTTTTTTTAGGTGACGAACAATAAGGCCTCCCTAACGGGGGGCCTTTTTTATTGATAACAAAAGCTTCCAGGAT<br>ACATAGATTACCACAACTCCGAGCCCTTCCACC

* Note that this gRNA does not bind to any valid sequence in the F^{C}_{-34} gate, but is labeled as such here because the gRNA was still expressed in the cells containing the F^{C}_{-34} and P^{C}_{-52} gates.

Supplemental Table 1: Sequences of all engineered circuit components.
